# Hypericum Genus as a Natural Source for Biologically Active Compounds

**DOI:** 10.3390/plants11192509

**Published:** 2022-09-26

**Authors:** Gonçalo Infante Caldeira, Luís Pleno Gouveia, Rita Serrano, Olga Duarte Silva

**Affiliations:** Research Institute for Medicines (iMed.ULisboa), Faculty of Pharmacy, Universidade de Lisboa, 1649-003 Lisbon, Portugal

**Keywords:** acylphloroglucinols, anticancer, biological activity, *Hypericum*, natural products

## Abstract

*Hypericum* L. genus plants are distributed worldwide, with numerous species identified throughout all continents, except Antarctica. These plant species are currently used in various systems of traditional medicine to treat mild depression, wounds and burns, diarrhea, pain, fevers, and their secondary metabolites previously shown, and the in vitro and/or in vivo cytotoxic, antimicrobial, anti-inflammatory, antioxidant, antihyperglycemic, and hepatoprotective activities, as well as the acetylcholinesterase and monoamine oxidase inhibitory activities. We conducted a systematic bibliographic search according to the Cochrane Collaboration guidelines to answer the question: “What is known about plants of *Hypericum* genus as a source of natural products with potential clinical biological activity?” We documented 414 different natural products with confirmed in vitro/in vivo biological activities, and 58 different *Hypericum* plant species as sources for these natural products. Phloroglucinols, acylphloroglucinols, xanthones, and benzophenones were the main chemical classes identified. The selective cytotoxicity against tumor cells, cell protection, anti-inflammatory, antimicrobial, antidepressant, anti-Alzheimer’s, and adipogenesis-inhibition biological activities are described. Acylphloroglucinols were the most frequent compounds with anticancer and cell-protection mechanisms. To date, no work has been published with a full descriptive list directly relating secondary metabolites to their species of origin, plant parts used, extraction methodologies, mechanisms of action, and biological activities.

## 1. Introduction

*Hypericum* L. genus plants are distributed worldwide, with numerous species identified throughout all continents, except Antarctica, both in geographical areas of temperate climates and in high mountainous regions [1]. They have long been part of the medical therapeutic armamentarium, and some species are even mentioned in the “De Materia Medica” authored by Dioscorides (1st century) [2]. *Hypericum* species are currently used in various systems of traditional medicine to treat mild depression, wounds and burns, diarrhea, pain, fevers, and poisoning from venomous animal bites [3,4].

Traditional Chinese Medicine (or simply Chinese Medicine), which is one of the most well-structured and ancient systems of traditional medicine, utilizes 64 species of plants of the *Hypericum* genus (of which 33 are endemic to China) [5], including, to name just a few examples, *Hypericum attenuatum* Fisch. ex Choisy, *Hypericum erectum* Thunb., and *Hypericum japonicum* Thunb. Whole-plant decoctions are employed to treat hemoptysis, wounds, and burns. *Hypericum japonicum* (whole plant) is also used to treat jaundice, dysentery, and lung abscesses, and *Hypericum scabrum* L. (whole plant) is used for the treatment of hematemesis, hemafecia, and irregular menstruation [3].

In Portugal, 22 species of *Hypericum* genus plants have been identified so far [6]. The most used in local traditional medicine are the *Hypericum perforatum* L. herb, for depressive illnesses, and the *Hypericum androsaemum* L. herb, for hepatic disorders [7]. *Hypericum foliosum* Aiton, which is an endemic species in the Azores archipelago, is used by locals to treat hepatic disorders. This species belongs to the same section as *Hypericum androsaemum*, but until now, no studies have been conducted that focus on its use in Azores traditional medicine [8,9].

Out of all the *Hypericum* species, *Hypericum perforatum,* which is commonly known as St. John’s Wort, is one of the most widely employed medicinal plants by the publics of both more industrialized and less developed countries. It is described in the European, Chinese, and Indian pharmacopoeias, and it is commonly used for the control of mild depressive symptoms. This has led to intense research on its antidepressant activity in recent decades [10,11]. It is also used for the treatment of many health conditions, such as inflammation, biliary disorders, burns and skin diseases, diabetes, pain symptoms, such as migraines or headaches, among many others [3,4,10,12]. In traditional usage, formulations are produced from *Hypericum perfuratum* as aqueous or alcoholic extracts or infusions, as well as oils and tinctures, intended for ingestion, as well as for external treatments. 

The extensive and diverse data collected over time, granted the elaboration of a community herbal monograph on the *Hypericum perforatum* herb (St. John’s Wort, *Hyperici herba*) by the “Committee on Herbal Medicinal Products” of the “European Medicines Agency”, is known for both its well-established medicinal uses and traditional uses. On the well-established medical uses, the monograph states formulations as an herbal medicinal product for the treatment of mild-to-moderate depressive episodes. As a traditional herbal medicinal product, formulations are detailed for the relief of temporary mental exhaustion, for the symptomatic treatment of minor inflammations of the skin (such as sunburns), and as an aid in the healing of minor wounds, as well as for the symptomatic relief of mild gastrointestinal discomfort [13]. 

The plants of the *Hypericum* genus and their compounds exert in vitro and/or in vivo cytotoxic, antimicrobial, anti-inflammatory, antioxidant, antifungal, astringent, antihyperglycemic, and hepatoprotective activities, as well as acetylcholinesterase and monoamine oxidase inhibitory activities [3,4,12,14].

Hypericin and hyperforin are the most well-known compounds, and they originated from the *Hypericum* species as a source of natural products. Hyperforin regulates the expressions of genes related to depressive states, while hypericin actively reduces stress-induced behaviors and increases the extracellular brain concentrations of glutamate and acetylcholine [15,16,17]. Hyperforin also has antimicrobial activity against microorganisms, such as *Staphylococcus aureus*, *Streptococcus pyogenes, Enterococcus faecalis, Escherichia coli,* and *Pseudomonas aeruginosa* [18]. Both compounds are known to exert anticancer activity by increasing apoptosis and decreasing the cell viability in tumor-cell lines [19,20,21,22,23,24,25]. 

The concept of extracting and isolating natural products to use as single medicinal agents, or to serve as a base to develop synthetically derived compounds, is not new, and numerous compounds have been uncovered from *Hypericum* plants, showing distinct biological properties. These new compounds may eventually be employed in the treatment of diseases after appropriate studies on the effectiveness and safety, or they may serve as a model to synthesize new drugs. If proven viable, then they would enrich the therapeutic options available to modern allopathic medicine [12,14,26,27].

Significant advances in analytical technologies have expanded our capacities to evaluate plant extracts by establishing phytochemical profiles, and to identify their marker natural products, thus not only contributing to a better understanding of medicinal plants, but also for the development of new synthetic compounds inspired in molecular structures of natural origin [28,29]. 

Under this framework, we conducted a systematic bibliographic search to answer the question: “What is known about plants of *Hypericum* genus as a source of natural products with potential clinical biological activity?”, focusing exclusively on studies that have confirmed the biological activities of isolated compounds, and describing the exact physiological parameter changes and how these changes were exerted. 

Previous works recently published on the *Hypericum* genus have shown that the plants from this genus have a wide variety of secondary metabolites with diverse biological activities [3,4,30,31]. However, to date, no work has been published with a full descriptive list directly relating secondary metabolites to their species of origin, plant parts used, extraction methodologies, mechanisms of action, and biological-activity outcomes. The aim of our work is to contribute useful information to fill in this gap.

## 2. Results

The information gathered with our bibliographic search is exhaustively presented in Table S1. The rows list the *Hypericum* species that are sources of natural products with confirmed biological activities. The columns indicate, from left to right, the compound class, followed by the compound found, method of measurement, in vitro or in vivo model used, outcome results of the measurements, comparative results within each study, possible therapeutic application, plant species, parts of the plant used, and type of extract. In the rightmost column, the reference citations of the study are described.

## 3. Materials and Methods

**Figure 1:**
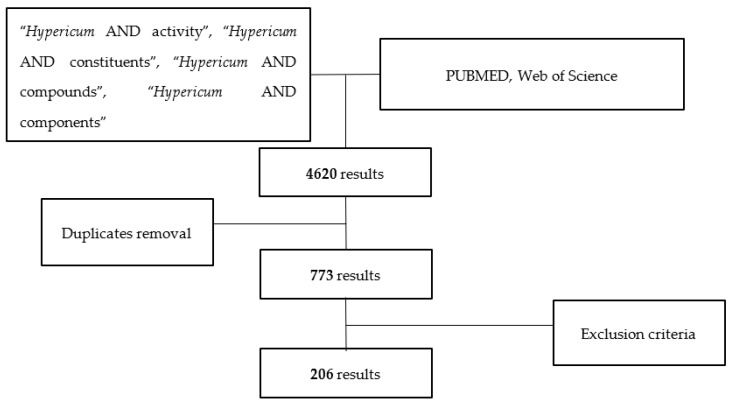
Data-screening methodology.

Our systematic review was conducted according to the Cochrane Collaboration guidelines on the subject. The data-screening methodology used is summarized in Figure 1. Scientific articles were retrieved by bibliographic searches of the Web of Science^TM^ and Pubmed^TM^ databases, applying several terms and the Boolean connector “AND”: “*Hypericum* AND activity”, “*Hypericum* AND constituents”, “*Hypericum* AND compounds”, and “*Hypericum* AND components, in a time window between January 2010 and April 2022. After the search, duplicates were removed, and specific evaluation parameters were applied to check whether the selected articles fulfilled the established criteria. Abstracts were assessed to understand the pertinence of the information. Research articles focusing on culture studies, the distributions of compounds in different plant tissues, ethnobotanical inquiries, compound synthesis, the impact of external factors on the synthesis of the plant secondary metabolites, extraction methods and yields, market studies, insecticidal potential, genomics, and works not related to the biological activities of isolated compounds were excluded. Articles centered on medicinal plants not belonging to the *Hypericum* genus, or medicinal plant mixtures, were also excluded. Finally, the scientific articles that confirmed the biological activities of secondary metabolites originating in species from the *Hypericum* genus were selected, regardless of known or newly discovered compounds. From this final pool of scientific articles, only those in which the biological activities of secondary metabolites were considered relevant to the authors, in comparison with the substances used as controls, were included. All the plant species’ names were confirmed on the website www.theplantlist.org (last accessed on 30 April 2022) [32].

The first step of the search found 4620 articles; after the removal of duplicates, 773 remained. Out of these, 206 fulfilled the criteria to be used for our study.

## 4. Hypericum Genus Plants Isolated Compounds with In Vivo/In Vitro Activities

We documented 414 different natural products with confirmed in vitro/in vivo biological activities, and 58 different plant species as sources for these natural products.

Several compounds with confirmed in vitro/in vivo activities were not isolated from a specific plant species but are very well known to belong to the plants from the *Hypericum* genus (namely, hypericin and hyperforin). Regarding specific plant species as sources of biologically active natural products, *Hypericum sampsonii* Hance was the species from which more compounds were isolated and tested (39 compounds), followed by *Hypericum perforatum* (33 compounds), *Hypericum scabrum* L. (30 compounds), and *Hypericum japonicum* Thunb. (22 compounds). 

The compounds isolated from *Hypericum sampsonii* showed selective in vitro cytotoxicity against tumor-cell lines and in vitro anti-inflammatory activity. The cell-protection activity in vitro was also observed for some of these compounds, resulting in increased cell viability. The secondary metabolites from *H. perforatum* showed important in vitro activity in Alzheimer-related mechanisms, such as acetylcholinesterase inhibition, in vitro antimicrobial activity, in vitro/in vivo antidepressant activity by gene-expression regulation, and in vitro selective cytotoxicity against tumor-cell lines. The secondary metabolites from *Hypericum scabrum* showed in vitro cell-protection activity through the cell-viability increase. *H. japonicum* is a source of compounds with antimicrobial activity in vitro, and in vitro cell-protection activity by increasing the cell viability and decreasing the oxidative stress and reactive oxygen species formation.

The compounds isolated from *H. attenuatum* exhibited in vitro selective cytotoxicity and increased apoptosis against tumor-cell lines. The compounds from *H. uralum* showed in vitro antidepressant activity, in vitro cell-protection activity, and in vitro acetylcholinesterase inhibitory activity. 

### 4.1. Class Compounds of Isolated Metabolites

The identified secondary metabolites fit into a wide variety of chemical classes: most belong to the acylphloroglucinols class (224 compounds), followed by xanthones (35 compounds), phloroglucinols (29 compounds), and flavonoids (22 compounds). We categorized the compounds according to their biological activities and based on the mechanisms of action shown. In this way, we had a wider picture of the clinical potential of the natural products originating from plants belonging to the *Hypericum* genus. Compounds with in vitro anticancer biological activity were the most frequent (145 compounds), followed by in vitro cell-protection (142 compounds), in vitro anti-inflammatory (60 compounds), in vitro antimicrobial (40 compounds), in vitro antidiabetic (29 compounds), in vitro antidepressant (22 compounds), in vitro anti-Alzheimer’s (21 compounds), and in vitro adipogenesis-inhibition (20 compounds) biological activities. A total of 13 other biological activities were identified with lesser frequency, of which skin healing and antiviral biological activities should be mentioned.

### 4.2. Class Compounds and Biological Activities

The phloroglucinols showed in vitro cytotoxicity against tumor-cell lines (fourteen compounds), in vitro cell protection (eight compounds), in vitro antidiabetic activity, through the decrease in the PTP1B activity (four compounds), and in vitro anti-inflammatory activity through mechanisms that lead to increased cell viability (two compounds). Similarly, the benzophenone compounds showed in vitro selective cytotoxicity against tumor-cell-line (eight compounds), cell-protection (nine compounds), anti-inflammatory (two compounds), antimalaria (one compound), and skin-healing (one compound) in vitro activities.

A total of 92 acylphloroglucinols, a subclass of phloroglucinols, showed in vitro cell-protection mechanisms, while 77 showed in vitro selective cytotoxicity against tumor-cell lines. The other compounds from this subclass showed a potential application on Alzheimer’s disease through the in vitro inhibition of the acetylcholinesterase activity; acylphloroglucinols also frequently exhibited in vitro antibacterial activity by decreasing the bacterial viability (21 compounds). Compounds with mechanisms related to in vitro antidepressant activity (namely, compounds that act on motor coordination, memory, or depressive behaviors) are also worthy of mention (14 compounds).

Xanthone compounds were frequently associated with in vitro selective cytotoxicity against tumor-cell lines (18 compounds), while others showed in vitro anti-inflammatory (8 compounds) and antimalaria mechanisms (2 compounds), being active against all the tested *Plasmodium falciparum* strains. In vitro antibacterial activity was also observed.

As mentioned before, hypericin and hyperforin are two of the most well-known secondary metabolites isolated from plants from the *Hypericum* genus. In our review, we were able to confirm the importance of these two secondary metabolites, to which several types of biological activities have been attributed. Hypericin predominantly showed in vitro cell-protection and anticancer activities, followed by in vitro antidepressant activity and photodynamic-therapy applications. Hyperforin was most frequently related to in vitro cell-protection, anticancer, antidiabetic, and antidepressant activities.

Our work also showed that hypericin, hyperforin, hyperoside, quercetin, and uliginosin B were the compounds more frequently identified and studied in the scientific articles analyzed, being cited in 23, 18, 17, 11, and 7 papers, respectively.

## 5. Comments

Several plants of the *Hypericum* genus are a source of natural products with clearly proven in vitro/in vivo biological activities.

Most of the studies we evaluated did not follow an ethnopharmacology rationale, which means that they did not focus on the confirmation of a certain biological activity related to the traditional medicinal use of the plant. Rather, they were wide phytochemical screenings intended to identify and characterize new natural products, which were then tested for a certain biological activity. Consequently, several of the assessed studies lacked information about which part of the plant and which solvent were used for the extraction. This information is crucial to determine the exact origin of natural products because their distributions vary within the plant parts.

Some studies have identified previously unknown compounds and have tested them for in vitro biological activities, of which no activity or low activity was observed. These compounds were not included in this review. However, testing such compounds for a wider variety of biological activities may be useful for a better understanding of their potentials.

Our review elicited that the tested natural products are frequently superior to the controls used to evaluate the biological activity in question. Further studies should be conducted utilizing other drugs used in clinical practice as controls to confirm the advantages of some plant compounds in comparison with synthetic drugs. 

The identification of the isolated natural products’ biological activities constitutes a key first step to understanding exactly how these compounds may be useful to a concrete clinical application. 

Concerning the potential clinical uses of *Hypericum* compounds, we must highlight some of the properties verified in the reviewed papers. The in vitro cell-protection and anti-inflammatory mechanisms exhibited by isolated compounds belonging to several compound classes were frequently correlated to an increase in the cell viability in the studied models. This increase in the cell viability was related to the ability of the compounds to decrease the induced oxidative stress, as well as the reactive oxygen species and nitric oxide production, or even modulate the gene expression to downregulate the enzymatic activity and cytokines related to the inflammatory process.

The in vitro anticancer activity was mostly shown by one of two different pathways. *Hypericum* compounds exhibited both the ability to exert direct selective cytotoxicity towards tumor-cell lines, which resulted in a decrease in such cell lines, and the ability to modulate several cellular mechanisms related to tumor progression, such as cytokine expression, apoptosis, gene expression, and cell migration. Most of the compounds exhibiting in vitro anticancer activity belong to the acylphloroglucinol class.

Acylphloroglucinols with anti-Alzheimer’s activity frequently exerted their in vitro activity by inhibiting the acetylcholinesterase activity and regulating the gene expression involved in the production of cytokines related to disease mechanisms, such as *β*-amyloid formation and deposition.

Acylphloroglucinols, as well as other compounds with in vitro antidepressant activity, can act through a wide variety of mechanisms. For instance, they showed the ability to regulate the genes involved in the mechanisms of depression, such as monoamine oxidase expression and receptor activation. Serotonin and noradrenaline reuptakes were also modulated by *Hypericum* compounds.

The microorganisms most frequently tested for susceptibility to the *Hypericum* compounds’ antimicrobial activity were methicillin-susceptible *Staphylococcus aureus,* methicillin-resistant *Staphylococcus aureus*, *Bacillus subtilis,* and *Enterococcus faecalis*.

Acylphloroglucinols, flavonoids, and anthraquinones, involved in adipogenesis inhibition, showed in vitro abilities to decrease the pancreatic lipase activity and intracellular lipid accumulation, thus causing a decrease in adipose-tissue formation.

To this day, very few compounds have been tested for their antiviral activity. Our search showed that only nine compounds exhibited in vitro antiviral activity, and mostly due to the decreased viral RNAse H and RNA-dependent DNA polymerase activities that lead to a decrease in viral replication. More studies assessing the potential antiviral activity of *Hypericum* compounds would be of extreme pertinence, namely, to find potential candidates to fight SARS-CoV2 infection.

Additional studies are required to assess the human toxicity, potential to interact with other drugs, dosage, and effectiveness, amongst other pharmacological and physiological parameters. 

Due to the current difficulty of developing new drugs through high-throughput synthesis and combinatorial chemistry-based drug development methods, screening natural products for their molecular targets and mechanisms of action can be an effective way to obtain useful information for synthesizing new derived compounds with greater bioavailability and effectiveness, or lower toxicity, than the original natural products. This, in turn, would spare copious amounts of time and financial investment when compared with creating new pharmaceutical agents from scratch.

## 6. Conclusions

As a conclusion of our exhaustive review, we can state that plants from the genus *Hypericum* are good sources of natural products with potential clinical biological activities. Besides hypericin and hyperforin, phloroglucinol, acylphloroglucinol, xanthone, and benzophenone compounds were the products obtained from *Hypericum* genus plants, and their biological activities were precisely evaluated.

The biological activities observed for natural products isolated from *Hypericum* species only partially justify their use in traditional medicine. In some instances, the natural products exhibited biological activities not related to the medicinal use of the plant, and further research is needed to widen their therapeutic usage.

## Data Availability

Not applicable.

## Supplementary Materials

The following supporting information can be downloaded at: https://www.mdpi.com/article/10.3390/plants11192509/s1, Table S1: Biological activity of secondary metabolites identified in *Hypericum* genus plants [33,34,35,36,37,38,39,40,41,42,43,44,45,46,47,48,49,50,51,52,53,54,55,56,57,58,59,60,61,62,63,64,65,66,67,68,69,70,71,72,73,74,75,76,77,78,79,80,81,82,83,84,85,86,87,88,89,90,91,92,93,94,95,96,97,98,99,100,101,102,103,104,105,106,107,108,109,110,111,112,113,114,115,116,117,118,119,120,121,122,123,124,125,126,127,128,129,130,131,132,133,134,135,136,137,138,139,140,141,142,143,144,145,146,147,148,149,150,151,152,153,154,155,156,157,158,159,160,161,162,163,164,165,166,167,168,169,170,171,172,173,174,175,176,177,178,179,180,181,182,183,184,185,186,187,188,189,190,191,192,193,194,195,196,197,198,199,200,201,202,203,204,205,206,207,208,209,210,211,212,213,214,215,216,217,218,219,220,221,222,223,224,225,226,227].
