# Supplementary material for: Hypericum Genus as a Natural Source for Biologically Active Compounds
Source: Plants (Basel). 2022 Sep 26;11(19):2509. doi: 10.3390/plants11192509 (PMC9573133; doi:10.3390/plants11192509)
Supplement: Supplementary file 1 [file plants-11-02509-s001.zip › plants-1917752-supplementary.pdf]

Table S1. Biological activity of secondary metabolites identified in *Hypericum* genus plants

Compound Class	Compound	Measurement	Method	Outcome		Therapeutic Application	Species, Plant Part and Extract	Ref #
**Acylphloroglucinol**	Hyperacmosin A	Cell viability	*In vitro* SK-N-SH cells	**Increased** cell viability	Survival rate: 68.20% vs 62.10 donepezil control vs 63.20 % PHPB control	CP	*Hypericum acmosepalum* N.Robson, AP, EtOH	[33]
**Acylphloroglucinol**	Sampsonione J	Cell viability	*In vitro* HepG2 cell lines	**Increased** cell viability **Decreased** paracetamol induced damage	64.37% *vs* 60.12% bicyclol control	CP	*Hypericum acmosepalum* N.Robson, AP, EtOH	[34]
**Acylphloroglucinol**	Sampsonione C	Cell viability	*In vitro* HepG2 cell lines	**Increased** cell viability **Decreased** paracetamol induced damage	61.62% *vs* 60.12% bicyclol control	CP	*Hypericum acmosepalum* N.Robson, AP, EtOH	[34]
**Acylphloroglucinol**	Hyperacmosin H	Cell viability	*In vitro* HepG2 cell lines	**Increased** cell viability **Decreased** paracetamol induced damage	60.38% *vs* 60.12% bicyclol control	CP	*Hypericum acmosepalum* N.Robson, AP, EtOH	[35]
**Acylphloroglucinol**	Hyperascyrin B	Cell viability	*In vitro* HepG2 cell lines	**Increased** cell viability **Decreased** paracetamol induced damage	61.56% *vs* 60.12% bicyclol control	CP	*Hypericum acmosepalum* N.Robson, AP, EtOH	[35]

Compound Class	Compound	Measurement	Method	Outcome		Therapeutic Application	Species, Plant Part and Extract	Ref #
**Acylphloroglucinol**	Hyperacmosin C	Cell viability	*In vitro* HepG2 paracetamol induced cell damage	**Increased** cell viability	**Decreased** paracetamol induced cell damage	CP	*Hypericum acmosepalum* N.Robson, AP, EtOH	[36]
**Acylphloroglucinol**	Hyperannulatin A	Cell viability	*In vitro*<br>HL-60, HL-60/DOX, MDA-MB, SKW-3, K-562 cells	**Decreased** cell viability (Selective activity)	IC$_{50}$ = 5.87 - 3.42 µM vs<br>IC$_{50}$ = 42.34 - 1.27 µM Etoposide control<br>IC$_{50}$ = 0.64 - 0.11 µM Podophyllotoxin control	AC	*Hypericum afromontanum* Bullock, AP, n-Hexane	[37]
**Acylphloroglucinol**	Hyperannulatin B	Cell viability	*In vitro*<br>HL-60, HL-60/DOX, MDA-MB, SKW-3, K-562 cells	**Decreased** cell viability (Selective activity)	IC$_{50}$ = 31.09 - 1.48 µM vs<br>IC$_{50}$ = 42.34 - 1.27 µM Etoposide control<br>IC$_{50}$ = 0.64 - 0.11 µM Podophyllotoxin control	AC	*Hypericum afromontanum* Bullock, AP, n-Hexane	[37]
**Acylphloroglucinol**	Hyperannulatin C	Cell viability	*In vitro*<br>HL-60, HL-60/DOX, MDA-MB, SKW-3, K-562 cells	**Decreased** cell viability (Selective activity)	IC$_{50}$ = 81.29 - 4.67 µM vs<br>IC$_{50}$ = 42.34 - 1.27 µM Etoposide control<br>IC$_{50}$ = 0.64 - 0.11 µM Podophyllotoxin control	AC	*Hypericum afromontanum* Bullock, AP, n-Hexane	[37]
**Acylphloroglucinol**	Hyperannulatin D	Cell viability	*In vitro*<br>HL-60, HL-60/DOX, MDA-MB, SKW-3, K-562 cells	**Decreased** cell viability (Selective activity)	IC$_{50}$ = 36.35 - 14.36 µM vs<br>IC$_{50}$ = 42.34 - 1.27 µM Etoposide control<br>IC$_{50}$ = 0.64 - 0.11 µM Podophyllotoxin control	AC	*Hypericum afromontanum* Bullock, AP, n-Hexane	[37]

Compound Class	Compound	Measurement	Method	Outcome		Therapeutic Application	Species, Plant Part and Extract	Ref #
**Acylphloroglucinol**	Hyperannulatin E	Cell viability	*In vitro*<br>HL-60, HL-60/DOX, MDA-MB, SKW-3, K-562 cells	**Decreased** cell viability (Selective activity)	IC_{50} = 13.44 - 2.85 µM<br>vs<br>IC_{50} = 42.34 - 1.27 µM<br>Etoposide control<br>IC_{50} = 0.64 - 0.11 µM<br>Podophyllotoxin control	AC	*Hypericum afromontanum* Bullock, AP, n-Hexane	[37]
**Acylphloroglucinol**	Andinin A	Stress-induced depressive behaviours	*In vivo mouse model*	**Decreased** immobility time in FST		AD	*Hypericum andinum* Gleason, R, n-Hexane	[38]
**Acylphloroglucinol**	Hyperascyrin L	Cell viability	*In vitro*<br>SK-N-SH, HepG2 cells	Neuroprotective<br>**Increased**: SK-N-SH cell viability<br>Hepatoprotective<br>**Increased:** HepG2 cell viability	**Neuroprotective:** significant neuroprotection *vs* resveratrol positive control<br>**Hepatoprotective:** significant hepatoprotection *vs* bicyclol positive control	CP	*Hypericum ascyron* L., AP, EtOH	[39]
**Acylphloroglucinol**	Hyperascyrin M	Cell viability	*In vitro*<br>SK-N-SH and HepG2 cells	Neuroprotective<br>**Increased**: SK-N-SH cell viability<br>Hepatoprotective<br>**Increased:** HepG2 cell viability	**Neuroprotective:** significant neuroprotection *vs* resveratrol positive control<br>**Hepatoprotective:** significant hepatoprotection *vs* bicyclol positive control	CP	*Hypericum ascyron* L., AP, EtOH	[39]

Compound Class	Compound	Measurement	Method	Outcome		Therapeutic Application	Species, Plant Part and Extract	Ref #
**Acylphloroglucinol**	Hyperascyrin N	Cell viability	*In vitro* SK-N-SH and HepG2 cells	Neuroprotective **Increased**: SK-N-SH cell viability Hepatoprotective **Increased:** HepG2 cell viability	**Neuroprotective:** significant neuroprotection *vs* resveratrol positive control **Hepatoprotective:** significant hepatoprotection *vs* bicyclol positive control	CP	*Hypericum ascyron* L., AP, EtOH	[39]
**Acylphloroglucinol**	Hypascyrin A	Bacterial susceptibility	*In vitro* Methicillin-resistant *Staphylococcus aureus, Bacillus subtilis*	Active against *S. aureus*, *B. subtilis*	MIC= 4.0 µM *S. aureus* MIC= 4.0 µM *B. subtilis*	AM	*Hypericum ascyron* L., R, MeOH	[40]
**Acylphloroglucinol**	Hypascyrin C	Bacterial susceptibility	*In vitro* Methicillin-resistant *Staphylococcus aureus, Bacillus subtilis*	Active against *S. aureus*, *B. subtilis*	MIC= 8.0 µM *S. aureus* MIC= 4.0 µM *B. subtilis*	AM	*Hypericum ascyron* L., R, MeOH	[40]
**Acylphloroglucinol**	Hypascyrin E	Bacterial susceptibility	*In vitro* Methicillin-resistant *Staphylococcus aureus, Bacillus subtilis*	Active against *S. aureus*, *B. subtilis*	MIC= 2.0 µM *S. aureus* MIC= 2.0 µM *B. subtilis*	AM	*Hypericum ascyron* L., R, MeOH	[40]
**Acylphloroglucinol**	Hyphenrone J	Bacterial susceptibility	*In vitro* Methicillin-resistant *Staphylococcus aureus, Bacillus subtilis*	Active against *S. aureus*, *B. subtilis*	MIC= 4.0 µM *S. aureus* MIC= 4.0 µM *B. subtilis*	AM	*Hypericum ascyron* L., R, MeOH	[40]

Compound Class	Compound	Measurement	Method	Outcome		Therapeutic Application	Species, Plant Part and Extract	Ref #
**Acylphloroglucinol**	Hyphenrone K	Bacterial susceptibility	*In vitro* Methicillin-resistant *Staphylococcus aureus, Bacillus subtilis*	Active against *S. aureus, B. subtilis*	MIC= 4.0 µM *S. aureus* MIC= 1.0 µM *B. subtilis*	AM	*Hypericum ascyron* L., R, MeOH	[40]
**Acylphloroglucinol**	Norascyronone A	Cell viability	*In vitro* PANC-1, SK-BR-3 cells	**Decreased** cell viability	IC_{50}= 4.3 µM against SK-BR-3 IC_{50}= 8.4 µM against PANC-1 vs taxinol	AC	*Hypericum ascyron* L., AP, MeOH	[41]
**Acylphloroglucinol**	Norascyronone B	Cell viability	*In vitro* ECA-109, SK-BR-3 cells	**Decreased** cell viability	IC_{50}= 7.8 µM against SK-BR-3 IC_{50}= 12.7 µM against ECA-109 vs taxinol	AC	*Hypericum ascyron* L., AP, MeOH	[41]
**Acylphloroglucinol**	Hyperascyrin A	Cell viability	*In vitro* SK-N-SH cells	**Increased** cell viability	SK-N-SH Cell viability 82.9±8.7 % vs 82.5 ± 1.2 % resveratrol control	CP	*Hypericum ascyron* L., AP, EtOH	[42]
**Acylphloroglucinol**	Hyperascyrin H	Cell viability	*In vitro* SK-N-SH, HeG2 cells	**Increased** cell viability	SK-N-SH Cell viability 78.2±0.5 % vs 82.5 ± 1.2 % resveratrol control HepG2 Cell viability 50.3±1.3 % vs 45.6±0.4 % bicyclol control	CP	*Hypericum ascyron* L., AP, EtOH	[42]
**Acylphloroglucinol**	Hyperascyrin I	Cell viability	*In vitro* HepG2 cells	**Increased** cell viability	HepG2 Cell viability 51.2±1.4 % vs 45.6 ± 0.4 % bicyclol control	CP	*Hypericum ascyron* L., AP, EtOH	[42]

Compound Class	Compound	Measurement	Method	Outcome		Therapeutic Application	Species, Plant Part and Extract	Ref #
**Acylphloroglucinol**	Hyperattenin A	Cell viability	*In vitro* HL-60, SMMC-7721, A-549, MCF-7, SW-480 cells	**Decreased** cell viability (selective activity)	IC_{50} = 28.96 - 9.62µM vs 15.23 - 2.12µM Cysplatin control vs <0.008 - <0.00µM Paclitaxel control	AC	*Hypericum attenuatum* Fisch. ex Choisy, AP, EtOH	[43]
**Acylphloroglucinol**	Hyperattenin B	Cell viability	*In vitro* HL-60, SMMC-7721, A-549, MCF-7 cells	**Decreased** cell viability (selective activity)	IC_{50} = 23.15 - 15.26µM vs 15.23 - 2.12µM Cysplatin control vs <0.008 - <0.00µM Paclitaxel control	AC	*Hypericum attenuatum* Fisch. ex Choisy, AP, EtOH	[43]
**Acylphloroglucinol**	Hyperattenin C	Cell viability	*In vitro* HL-60, SMMC-7721, A-549, MCF-7 cells	**Decreased** cell viability (selective activity)	IC_{50} = 35.34 - 16.20µM vs 15.23 - 2.12µM Cysplatin control vs <0.008 - <0.00µM Paclitaxel control	AC	*Hypericum attenuatum* Fisch. ex Choisy, AP, EtOH	[43]
**Acylphloroglucinol**	Hyperattenin D	Cell viability	*In vitro* SMMC-7721, A-549, MCF-7 cells	**Decreased** cell viability (selective activity)	IC_{50} = 30.36 - 19.31µM vs 15.23 - 7.47µM Cysplatin control vs <0.008 - <0.00µM Paclitaxel control	AC	*Hypericum attenuatum* Fisch. ex Choisy, AP, EtOH	[43]
**Acylphloroglucinol**	Hyperattenin E	Cell viability	*In vitro* HL-60, A-549, MCF-7 cells	**Decreased** cell viability (selective activity)	IC_{50} = 31.6 - 30.89µM vs 15.23 - 2.12µM Cysplatin control vs <0.00µM Paclitaxel control	AC	*Hypericum attenuatum* Fisch. ex Choisy, AP, EtOH	[43]
**Acylphloroglucinol**	Hyperattenin H	Cell viability	*In vitro* SMMC-7721 cells	**Decreased** cell viability (selective activity)	IC_{50}= 20.51µM vs 7.47µM Cysplatin control vs <0.008µM Paclitaxel control	AC	*Hypericum attenuatum* Fisch. ex Choisy, AP, EtOH	[43]

Compound Class	Compound	Measurement	Method	Outcome		Therapeutic Application	Species, Plant Part and Extract	Ref #
**Acylphloroglucinol**	Hyperattenin I	Cell viability	*In vitro*<br>HL-60, SMMC-7721, A-549, MCF-7, SW-480 cells	**Decreased** cell viability (selective activity)	IC_{50} = 15.88 - 2.04μM vs 15.23 - 2.12μM Cysplatin control vs <0.008 - <0.00μM Paclitaxel control	AC	*Hypericum attenuatum* Fisch. ex Choisy, AP, EtOH	[43]
**Acylphloroglucinol**	Hyperberin A	Cell viability, Oxidative stress	*In vitro*<br>HCT116, RAW 264.7 cells	**Decreased** HCT 116 cell viability<br>Anti-inflammatory activity on LPS induced RAW264.7 cells	**Decreased**: NO production in RAW264.7 cells (IC_{50}= 7.36 ± 0.97 µM vs IC_{50}= 39.97 ± 0.1.32 µM NMMA positive control)<br>**Increased**: RAW264.7 cell viability	AC, AI	*Hypericum beanii* N. Robson, R, EtOH	[44]
**Acylphloroglucinol**	Hyperberin B	Cell viability, Oxidative stress	*In vitro*<br>HCT116, RAW 264.7 cells	**Decreased** HCT 116 cell viability<br>Anti-inflammatory activity on LPS induced RAW264.7 cells	**Decreased**: NO production in RAW264.7 cells (IC_{50}= 14.00 ± 0.14 µM vs IC_{50}= 39.97 ± 0.1.32 µM NMMA positive control)<br>**Increased**: RAW264.7 cell viability	AC, AI	*Hypericum beanii* N. Robson, R, EtOH	[44]
**Acylphloroglucinol**	Hyperbeanol B	Cell viability	*In vitro*<br>K562 cells	**Decreased** cell viability (modest)	IC_{50}=16.9 µM	AC	*Hypericum beanii* N. Robson, AP, MeOH	[45]
**Acylphloroglucinol**	Hyperbeanol D	Cell viability	*In vitro*<br>K562 cells	**Decreased** cell viability (modest)	IC_{50}=20.7 µM	AC	*Hypericum beanii* N. Robson, AP, MeOH	[45]

Compound Class	Compound	Measurement	Method	Outcome	Therapeutic Application	Species, Plant Part and Extract	Ref #
**Acylphloroglucinol**	Bellumone Q	Oxidative stress, Adipogenesis	*In vitro* RAW264.7 cell lines, L02 cell lines	**Decreased** LPS-induced NO production **Decreased** intracellular lipid acumulation	CP, Ad.In	*Hypericum bellum* H.L.Li, Fl, EtOH	[46]
**Acylphloroglucinol**	Bellumone J	Radical scavenging	*In vitro* RAW264.7 cell lines, Radical scavenging capacity model	**Decreased** ROS titers	CP	*Hypericum bellum* H.L.Li, Fl, EtOH	[46]
**Acylphloroglucinol**	Bellumone N	Radical scavenging	*In vitro* RAW264.7 cell lines, Radical scavenging capacity model	**Decreased** ROS titers	CP	*Hypericum bellum* H.L.Li, Fl, EtOH	[46]
**Acylphloroglucinol**	Bellumone O	Radical scavenging	*In vitro* RAW264.7 cell lines, Radical scavenging capacity model	**Decreased** ROS titers	CP	*Hypericum bellum* H.L.Li, Fl, EtOH	[46]

Compound Class	Compound	Measurement	Method	Outcome		Therapeutic Application	Species, Plant Part and Extract	Ref #
**Acylphloroglucinol**	Chinesin I	Radical scavenging	*In vitro* RAW264.7 cell lines, Radical scavenging capacity model	**Decreased** ROS titers		CP	*Hypericum bellum* H.L.Li, Fl, EtOH	[46]
**Acylphloroglucinol**	Chinesin II	Radical scavenging	*In vitro* RAW264.7 cell lines, Radical scavenging capacity model	**Decreased** ROS titers		CP	*Hypericum bellum* H.L.Li, Fl, EtOH	[46]
**Acylphloroglucinol**	Bellumone D	Adipogenesis	*In vitro* L02 cell model	**Decreased** intracellular lipid acumulation		Ad.In	*Hypericum bellum* H.L.Li, Fl, EtOH	[46]
**Acylphloroglucinol**	Bellumone K	Adipogenesis	*In vitro* L02 cell model	**Decreased** intracellular lipid acumulation		Ad.In	*Hypericum bellum* H.L.Li, Fl, EtOH	[46]
**Acylphloroglucinol**	Hyperibone J	Adipogenesis	*In vitro* L02 cell model	**Decreased** intracellular lipid acumulation		Ad.In	*Hypericum bellum* H.L.Li, Fl, EtOH	[46]
**Acylphloroglucinol**	Uliginosin B	Cell viability	*In vitro* OVCAR-3, NCI-ADR/RES, UACC-62, MCF-7, 786-0,NCI-H460, PC-3, HT29 cells	**Decreased** cell proliferation (selective activity)	Mean TGI= 3.91μg/mL vs 0.88μg/mL Doxorubicine control	AC	*Hypericum brasiliense* Choisy, AP, n-Hexane	[47]

Compound Class	Compound	Measurement	Method	Outcome		Therapeutic Application	Species, Plant Part and Extract	Ref #
**Acylphloroglucinol**	Otogirinin B	Cell viability	*In vitro*<br>A549 cells	**Decreased** A549 cell viability	-	AC	*Hypericum choisianum* Wall. Ex N.Robson, AP, EtOH	[48]
**Acylphloroglucinol**	Hypercohin B	Cell viability	*In vitro*<br>HL-60, A-549, MCF-7, SW480 cells	**Decreased** cell viability (selective toxicity)	IC_{50} = 15.6 - 5.8µM vs IC_{50} = 18.7 - 1.8µM Cisplatin control IC_{50} = 0.1 - <0.008µM Paclitaxel control	AC	*Hypericum cohaerens* N.Robson, AP, MeOH	[49]
**Acylphloroglucinol**	Hypercohin C	Cell viability	*In vitro*<br>HL-60, SMMC-7721, A-549, SW480 cells	**Decreased** cell viability (selective toxicity)	IC_{50} = 17.9 - 8.2µM vs IC_{50} = 15.6 - 1.8µM Cisplatin control IC_{50} = 0.1 - <0.008µM Paclitaxel control	AC	*Hypericum cohaerens* N.Robson, AP, MeOH	[49]
**Acylphloroglucinol**	Hypercohin D	Cell viability	*In vitro*<br>HL-60, SMMC-7721, A-549, MCF-7, SW480 cells	**Decreased** cell viability (selective toxicity)	IC_{50} = 9.5 - 5.6µM vs IC_{50} = 15.6 - 1.8µM Cisplatin control IC_{50} = 0.1 - <0.008µM Paclitaxel control	AC	*Hypericum cohaerens* N.Robson, AP, MeOH	[49]
**Acylphloroglucinol**	Hyperelodione A	Cell viability	*In vitro* HeLa and MCF-7 cell lines	**Decreased** cell viability (selective cytotoxicity)**,** cell proliferation<br>**Increased** apoptosis	**Decreased** RXRα activation, cell growth	AC	*Hypericum elodeoides* Choisy, WP, EtOH	[50]
**Acylphloroglucinol**	Hyperelodione B	Cell viability	*In vitro* HeLa and MCF-7 cell lines	**Decreased** cell viability (selective cytotoxicity)	**Decreased** RXRα activation, cell growth	AC	*Hypericum elodeoides* Choisy, WP, EtOH	[50]

Compound Class	Compound	Measurement	Method	Outcome		Therapeutic Application	Species, Plant Part and Extract	Ref #
**Acylphloroglucinol**	Hyperelodione C	Cell viability	*In vitro* HeLa and MCF-7 cell lines	**Decreased** cell viability (selective cytotoxicity)	**Decreased** RXRα activation, cell growth	AC	*Hypericum elodeoides* Choisy, WP, EtOH	[50]
**Acylphloroglucinol**	Adotogirin	Bacterial susceptibility	*In vitro* *S. aureus* and *B. subtilis*	Active against all tested strains	MIC = 0.5–4.0 µg/mL vs MIC50=1.0 µg/mL positive control (*S. aureus*)<br>MIC= 2 µg/mL (*B. subtilis*)	AM	*Hypericum erectum* Thunb., R, MeOH	[51]
**Acylphloroglucinol**	Otogirin	Bacterial susceptibility	*In vitro* *S. aureus* and *B. subtilis*	Active against all tested strains	MIC = 0.5–8.0 µg/mL vs MIC50=1.0 µg/mL positive control (*S. aureus*)<br>MIC= 2 µg/mL (*B. subtilis*)	AM	*Hypericum erectum* Thunb., R, MeOH	[51]
**Acylphloroglucinol**	Otogirin	Cell viability	*In vitro* PANC-1 cells	**Decreased** PANC-1 cell viability	IC_{50}= 12.0 µM vs IC_{50}= 3.5 µM Taxol control	AC	*Hypericum faberi* R.Keller, WP, MeOH	[52]
**Acylphloroglucinol**	Uralione E	Adipogenesis, Expression modulation	*In vitro* L02 cell model	**Decreased** intracellular lipid acumulation	**Decreased** CD36 and FASN expression<br>**Increased** PPARα and ACOX1 expression	Ad.In, IM	*Hypericum forrestii* (Chitt.) N.Robson, Fr, EtOH	[53]
**Acylphloroglucinol**	Hypercohin K	Adipogenesis, Expression modulation	*In vitro* L02 cell model	**Decreased** intracellular lipid acumulation	**Decreased** CD36 and FASN expression<br>**Increased** PPARα and ACOX1 expression	Ad.In, IM	*Hypericum forrestii* (Chitt.) N.Robson, Fr, EtOH	[53]

Compound Class	Compound	Measurement	Method	Outcome		Therapeutic Application	Species, Plant Part and Extract	Ref #
**Acylphloroglucinol**	Hyperscabin D	Noradrenaline reuptake	*In vitro* synaptosome model	**Decreased** noradrenaline reuptake		AD	*Hypericum forrestii* (Chitt.) N.Robson, Fr, EtOH	[54]
**Acylphloroglucinol**	Hyperscabin F	Cell viability, Noradrenaline reuptake	*In vitro* synaptosome model, *In vitro* WB-F344 induced cell damage model	**Decreased** noradrenaline reuptake **Increased** cell viability	Cell survival rate:78%	AD, CP	*Hypericum forrestii* (Chitt.) N.Robson, Fr, EtOH	[54]
**Acylphloroglucinol**	Hyperscabin J	Noradrenaline reuptake	*In vitro* synaptosome model	**Decreased** noradrenaline reuptake		AD	*Hypericum forrestii* (Chitt.) N.Robson, Fr, EtOH	[54]
**Acylphloroglucinol**	Hyperscabin K	Cell viability, Noradrenaline reuptake	*In vitro* synaptosome model, *In vitro* WB-F344 induced cell damage model	**Decreased** noradrenaline reuptake **Increased** cell viability	Cell survival rate:77%	AD, CP	*Hypericum forrestii* (Chitt.) N.Robson, Fr, EtOH	[54]
**Acylphloroglucinol**	Hyperscabin L	Noradrenaline reuptake	*In vitro* synaptosome model	**Decreased** noradrenaline reuptake		AD	*Hypericum forrestii* (Chitt.) N.Robson, Fr, EtOH	[54]
**Acylphloroglucinol**	Hyphenrone A	Cell viability, Noradrenaline reuptake	*In vitro* synaptosome model, *In vitro* WB-F344 induced cell damage model	**Decreased** noradrenaline reuptake **Increased** cell viability	Cell survival rate:73%	AD, CP	*Hypericum forrestii* (Chitt.) N.Robson, Fr, EtOH	[54]

Compound Class	Compound	Measurement	Method	Outcome	Therapeutic Application	Species, Plant Part and Extract	Ref #
**Acylphloroglucinol**	Hyperuralone C	Noradrenaline reuptake	*In vitro* synaptosome model	**Decreased** noradrenaline reuptake	AD, CP	*Hypericum forrestii* (Chitt.) N.Robson, Fr, EtOH	[54]
**Acylphloroglucinol**	Hyphenrone T	Noradrenaline reuptake	*In vitro* synaptosome model	**Decreased** noradrenaline reuptake	AD	*Hypericum forrestii* (Chitt.) N.Robson, Fr, EtOH	[54]
**Acylphloroglucinol**	Hyperforin A	PTP1B Activity	*In vitro* PTP1B model	**Decreased** PTP1B activity	ADb	*Hypericum forrestii* (Chitt.) N.Robson, AP, EtOH	[55]
**Acylphloroglucinol**	Hyperforin B	PTP1B Activity	*In vitro* PTP1B model	**Decreased** PTP1B activity	ADb	*Hypericum forrestii* (Chitt.) N.Robson, AP, EtOH	[55]
**Acylphloroglucinol**	Hyperichoisin A	PTP1B Activity	*In vitro* PTP1B model	**Decreased** PTP1B activity	ADb	*Hypericum forrestii* (Chitt.) N.Robson, AP, EtOH	[55]
**Acylphloroglucinol**	Hypercohin G	PTP1B Activity	*In vitro* PTP1B model	**Decreased** PTP1B activity	ADb	*Hypericum forrestii* (Chitt.) N.Robson, AP, EtOH	[55]
**Acylphloroglucinol**	Sampsonione J	PTP1B Activity	*In vitro* PTP1B model	**Decreased** PTP1B activity	ADb	*Hypericum forrestii* (Chitt.) N.Robson, AP, EtOH	[55]

Compound Class	Compound	Measurement	Method	Outcome		Therapeutic Application	Species, Plant Part and Extract	Ref #
**Acylphloroglucinol**	Otogirinin A	PTP1B Activity	*In vitro* PTP1B model	**Decreased** PTP1B activity		ADb	*Hypericum forrestii* (Chitt.) N.Robson, AP, EtOH	[55]
**Acylphloroglucinol**	Sampsonione H	PTP1B Activity	*In vitro* PTP1B model	**Decreased** PTP1B activity		ADb	*Hypericum forrestii* (Chitt.) N.Robson, AP, EtOH	[55]
**Acylphloroglucinol**	13 - UNIDENTIFIED COMPOUND	AChE activity	*In vitro*	**Decreased** AChE activity	IC_{50}=37.2µM	Alz	*Hypericum henryi* H. Lév.&Vaniot, AP, MeOH	[56]
**Acylphloroglucinol**	Hyphenrone J	AChE activity, cell viability	*In vitro* HL-60, A-549, SMMC-7721, MCF-7, and SW-480 cells	**Decreased** AChE activity Selective cytotoxicity against all tested cells	AChE inhibition: IC_{50}=25.4µM Citotoxicity: IC_{50} = 7.0-1.7 µM vs IC_{50} <0.008 µM Taxol control	Alz, AC	*Hypericum henryi* H. Lév.&Vaniot, AP, MeOH	[56]
**Acylphloroglucinol**	Hypercalin C	Cell viability	*In vitro* HL-60, A-549, SMMC-7721, MCF-7, and SW-480 cells	Selective cytotoxicity against all tested cells	IC_{50} = 8.9-2.3 µM vs IC_{50} <0.008 µM Taxol control	AC	*Hypericum henryi* H. Lév.&Vaniot, AP, MeOH	[56]
**Acylphloroglucinol**	26 - UNIDENTIFIED COMPOUND	AChE activity	*In vitro*	**Decreased** AChE activity	IC_{50}=26.4µM	Alz	*Hypericum henryi* H. Lév.&Vaniot, AP, MeOH	[56]

Compound Class	Compound	Measurement	Method	Outcome		Therapeutic Application	Species, Plant Part and Extract	Ref #
**Acylphloroglucinol**	40 - UNIDENTIFIED COMPOUND	AChE activity	*In vitro*	**Decreased** AChE activity	IC_{50}=9.8µM	Alz	*Hypericum henryi* H. Lév.&Vaniot, AP, MeOH	[56]
**Acylphloroglucinol**	Hyperenol	Cell viability	*In vitro* HeLa, GFP-LC3 HeLa, YFP-Parkin HeLa and A549 cell lines	**Decreased** cell viability (selective cytotoxicity)	IC_{50}= 4.18±0.43 - 0.88±0.042 µM vs IC_{50}=31.31±0.76 - 2.98±108µM Etoposide control	AC	*Hypericum henryi* H. Lév.&Vaniot, WP, MeOH	[57]
**Acylphloroglucinol**	Hyphenrone J	Cell viability, cell proliferation	*In vitro* HeLa, GFP-LC3 HeLa, YFP-Parkin HeLa and A549 cell lines	**Decreased** cell viability (selective cytotoxicity)	**Increased** apoptosis, autophagy, PINK1/Parkin mediated mitphagy **Decreased** A549 cells metastasis *in vitro* IC_{50}= 1.85±0.18 - 0.07±0.04 µM vs IC_{50}=31.31±0.76 - 2.98±108µM Etoposide control	AC	*Hypericum henryi* H. Lév.&Vaniot, WP, MeOH	[57]
**Acylphloroglucinol**	Hyphenrone K	Cell viability, cell proliferation	*In vitro* HeLa, GFP-LC3 HeLa, YFP-Parkin HeLa and A549 cell lines	**Decreased** cell viability (selective cytotoxicity)	**Increased** apoptosis, autophagy, PINK1/Parkin mediated mitphagy **Decreased** A549 cells metastasis *in vitro* IC_{50}= 3.10±0.11 - 0.09±0.099 µM vs IC_{50}=31.31±0.76 - 2.98±108µM Etoposide control	AC	*Hypericum henryi* H. Lév.&Vaniot, WP, MeOH	[57]

Compound Class	Compound	Measurement	Method	Outcome		Therapeutic Application	Species, Plant Part and Extract	Ref #
**Acylphloroglucinol**	Hyphenrone E	Cell viability	*In vitro* HeLa, GFP-LC3 HeLa, YFP-Parkin HeLa and A549 cell lines	**Decreased** cell viability (selective cytotoxicity)	IC_{50}= 22.16±0.83 - 0.89±0.41 μM vs IC_{50}=31.31±0.76 - 2.98±108μM Etoposide control	AC	*Hypericum henryi* H. Lév.&Vaniot, WP, MeOH	[57]
**Acylphloroglucinol**	Uralodin C	Cell viability	*In vitro* HepG2, SGC7901, HL-60, and K562 cells	**Decreased** cell viability (selective activity)	IC_{50} = 32.1±2.1 - 14.3±1.3μM vs IC_{50} = 17.4±1.3 - 1.9±0.3μM Cisplatin control	AC	*Hypericum henryi* subsp. uraloides (Rehder) N.Robson, AP, MeOH	[58]
**Acylphloroglucinol**	Uralodin A	Cell viability	*In vitro* HepG2, SGC7901, HL-60, and K562 cells	**Decreased** cell viability (selective activity)	IC_{50} = 59.7±3.3 - 16.0±0.9μM vs IC_{50} = 17.4±1.3 - 1.9±0.3μM Cisplatin control	AC	*Hypericum henryi* subsp. uraloides (Rehder) N.Robson, AP, MeOH	[58]
**Acylphloroglucinol**	Furohyperforin	Cell viability	*In vitro* HepG2, SGC7901, HL-60, and K562 cells	**Decreased** cell viability (selective activity)	IC_{50} = 46.2±3.4 - 18.5±1.9μM vs IC_{50} = 17.4±1.3 - 1.9±0.3μM Cisplatin control	AC	*Hypericum henryi* subsp. uraloides (Rehder) N.Robson, AP, MeOH	[58]
**Acylphloroglucinol**	Hookerione K	Cell viability	*In vitro* 7402, BIU-87, ECA-109, HeLa-S3, PANC-1 cells	**Decreased** cell viability (selective activity)	IC_{50}=17.24μM against ECA-109	AC	*Hypericum hookerianum* Wight & Arn., AP, MeOH	[59]
**Acylphloroglucinol**	Hookerione L	Cell viability	*In vitro* 7402, BIU-87, ECA-109, HeLa-S3, PANC-1 cells	**Decreased** cell viability (selective activity)	IC_{50}=10.18μM against ECA-109	AC	*Hypericum hookerianum* Wight & Arn., AP, MeOH	[59]

Compound Class	Compound	Measurement	Method	Outcome		Therapeutic Application	Species, Plant Part and Extract	Ref #
**Acylphloroglucinol**	Hookerione N	Cell viability	*In vitro* 7402, BIU-87, ECA-109, HeLa-S3, PANC-1 cells	**Decreased** cell viability (selective activity)	IC_{50}=9.88µM against ECA-109	AC	*Hypericum hookerianum* Wight & Arn., AP, MeOH	[59]
**Acylphloroglucinol**	Hookerione O	Cell viability	*In vitro* 7402, BIU-87, ECA-109, HeLa-S3, PANC-1 cells	**Decreased** cell viability (selective activity)	IC_{50}=12.5µM against ECA-109; IC_{50}=13.37µM against Hela-S3	AC	*Hypericum hookerianum* Wight & Arn., AP, MeOH	[59]
**Acylphloroglucinol**	Hookerione Q	Cell viability	*In vitro* 7402, BIU-87, ECA-109, HeLa-S3, PANC-1 cells	**Decreased** cell viability (selective activity)	IC_{50}=8.27µM against ECA-109	AC	*Hypericum hookerianum* Wight & Arn., AP, MeOH	[59]
**Acylphloroglucinol**	Hookerianone A	USP Activity	*In vitro* USP7 model	**Decreased** USP activity	87% USP inhibition	AC	*Hypericum hookerianum* Wight & Arn., AP, EtOH	[60]
**Acylphloroglucinol**	Hookerianone E	USP Activity	*In vitro* USP7 model	**Decreased** USP activity	87% USP inhibition	AC	*Hypericum hookerianum* Wight & Arn., AP, EtOH	[60]
**Acylphloroglucinol**	Hypercalin C	USP Activity	*In vitro* USP7 model	**Decreased** USP activity	91% USP inhibition	AC	*Hypericum hookerianum* Wight & Arn., AP, EtOH	[60]
**Acylphloroglucinol**	Tomoeone A	USP Activity	*In vitro* USP7 model	**Decreased** USP activity	86% USP inhibition	AC	*Hypericum hookerianum* Wight & Arn., AP, EtOH	[60]

Compound Class	Compound	Measurement	Method	Outcome		Therapeutic Application	Species, Plant Part and Extract	Ref #
**Acylphloroglucinol**	Furohyperforin	USP Activity	*In vitro* USP7 model	**Decreased** USP activity	95% USP inhibition	AC	*Hypericum hookerianum* Wight & Arn., AP, EtOH	[60]
**Acylphloroglucinol**	Hyphenrone T	USP Activity	*In vitro* USP7 model	**Decreased** USP activity	79% USP inhibition	AC	*Hypericum hookerianum* Wight & Arn., AP, EtOH	[60]
**Acylphloroglucinol**	Oxepahyperforin	USP Activity	*In vitro* USP7 model	**Decreased** USP activity	83% USP inhibition	AC	*Hypericum hookerianum* Wight & Arn., AP, EtOH	[60]
**Acylphloroglucinol**	Hyperjaponicols A	Bacterial susceptibility	*In vitro Escherichia coli, Staphylococcus aureus, Salmonella typhimurium, Enterococcus faecalis*	Selective antimicrobial activity against bacterial species	MICs= 1.8µM (*E. coli, S. aureus, E. faecalis*), 0.9µM (*S. typhimurium*) vs Cefotaxime control MICs= 0.4µM (*E. coli, E. faecalis*), 3.3µM (*S. aureus, S. typhimurium*)	AM	*Hypericum japonicum* Thunb., WP, MeOH	[61]
**Acylphloroglucinol**	Hyperjaponicols B	Bacterial susceptibility	*In vitro Escherichia coli, Staphylococcus aureus, Salmonella typhimurium, Enterococcus faecalis*	Moderate lipase inhibitory activity Selective antimicrobial activity against bacterial species	MICs= 0.9µM (*E.coli*), 3.4µM (*S. aureus*), 1.7µM (*S. thyphimurium, E. faecalis*) vs Cefotaxime control MICs= 0.4µM (*E. coli, E. faecalis*), 3.3µM (*S. aureus, S. typhimurium*)	AM	*Hypericum japonicum* Thunb., WP, MeOH	[61]

Compound Class	Compound	Measurement	Method	Outcome		Therapeutic Application	Species, Plant Part and Extract	Ref #
**Acylphloroglucinol**	Hyperjaponicols C	Bacterial susceptibility	*In vitro Escherichia coli, Staphylococcus aureus, Salmonella typhimurium, Enterococcus faecalis*	Moderate lipase inhibitory activity Selective antimicrobial activity against bacterial species	MICs= 0.8µM (*E. coli, S. typhimurium, E. faecalis*). 3.3µM (*S. aureus*) vs Cefotaxime control MICs= 0.4µM (*E. coli. E. faecalis*), 3.3µM (*S. aureus, S. typhimurium*)	AM	*Hypericum japonicum* Thunb., WP, MeOH	[61]
**Acylphloroglucinol**	Hyperjaponicols D	Bacterial susceptibility	*In vitro Escherichia coli, Staphylococcus aureus, Salmonella typhimurium, Enterococcus faecalis*	Selective antimicrobial activity against bacterial species	MICs= 0.9µM (*E. coli, S. typhimurium, E. faecalis*), 1.7µM (*S. aureus*) vs Cefotaxime control MICs= 0.4µM (*E. coli, E. faecalis*), 3.3µM (*S. aureus, S. typhimurium*)	AM	*Hypericum japonicum* Thunb., WP, MeOH	[61]
**Acylphloroglucinol**	Hyperjapone A	Cell viability	*In vitro* HT22 cells; DPPH free radical scavenging activity model	**Decreased** induced ferroptosis		CP	*Hypericum japonicum* Thunb., WP, EtOH	[62]
**Acylphloroglucinol**	Hyperjovinol A	Radical scavenging	*In vitro*	Antioxidant activity comparable to that of vitamin C and vitamin E		CP	*Hypericum jovis* Greuter, -, -	[63]

Compound Class	Compound	Measurement	Method	Outcome		Therapeutic Application	Species, Plant Part and Extract	Ref #
**Acylphloroglucinol**	Petiolin J	Bacterial susceptibility	*In vitro Micrococcs luteus, Cryptococcus neoformans, Trichophyton mentagrophytes*	Active against all tested strains (selective activity)	MIC= 8 µg/mL (*Micrococcus luteus*) MIC=16 µg/mL (*Cryptococcus neoformans*) MIC=16 µg/mL (*Trichophyton mentagrophytes*)	AM	*Hypericum kiusianum* Koidz., AP, MeOH	[64]
**Acylphloroglucinol**	Longisglucinol A	Oxidative stress	*In vitro* RAW264.7 cell lines	**Decreased** LPS-induced NO production	IC_{50}= 9.46±1.21µM vs IC_{50}=6.70±0.58µM Dexamethasone control	CP	*Hypericum longistylum* Oliv., AP, EtOH	[65]
**Acylphloroglucinol**	Uliginosin C	Fungal susceptibility	*In vitro Candida albicans, C. parapsilosis, C. glabrata, C. lusitaniae, C. pararugosa* strains	**Decreased** fungal growth	MIC_{50}= >32 - 6±0.2 µM vs MIC_{50}=>208 - 0.13±0.0µM Fluconazole control	AF	*Hypericum mexicanum* L., R, L, S, MeOH	[66]
**Acylphloroglucinol**	3' Prenyl Uliginosin B	Fungal susceptibility	*In vitro Candida albicans, C. parapsilosis, C. glabrata, C. lusitaniae, C. pararugosa* strains	**Decreased** fungal growth	MIC_{50}= >30 - 3±0.2 µM vs MIC_{50}=>208 - 0.13±0.0µM Fluconazole control	AF	*Hypericum mexicanum* L., R, L, S, MeOH	[66]
**Acylphloroglucinol**	Hypermonin A	Cell viability	*In vitro* PC12 cells	**Decreased** corticosterone induced cell damage **Increased** cell viability	IC_{50}=20 µM	CP	*Hypericum monogynum* L., L, Tws, MeOH	[67]

Compound Class	Compound	Measurement	Method	Outcome		Therapeutic Application	Species, Plant Part and Extract	Ref #
**Acylphloroglucinol**	Hypermoin E	Expression modulation	*In vitro* HepG2/ADR and MCF-7/ADR cancer cell lines	**Decreased** multidrug resistance activity		IM, AC	*Hypericum monogynum* L., Fl, MeOH	[68]
**Acylphloroglucinol**	Hyperielliptone HA	Expression modulation	*In vitro* HepG2/ADR and MCF-7/ADR cancer cell lines	**Decreased** multidrug resistance activity		IM, AC	*Hypericum monogynum* L., Fl, MeOH	[68]
**Acylphloroglucinol**	Hypermonin C	Cell viability	*In vitro* SH-SY5Y and PC12 cell lines	**Increased** cell viability	**Decreased** induced cell damage	CP	*Hypericum monogynum* L., AP, MeOH	[69]
**Acylphloroglucinol**	Furoadhyperforin	Cell viability	*In vitro* SH-SY5Y and PC12 cell lines	**Increased** cell viability	**Decreased** induced cell damage	CP	*Hypericum monogynum* L., AP, MeOH	[69]
**Acylphloroglucinol**	Furohyperforin	Cell viability	*In vitro* SH-SY5Y and PC12 cell lines	**Increased** cell viability	**Decreased** induced cell damage	CP	*Hypericum monogynum* L., AP, MeOH	[69]
**Acylphloroglucinol**	Attenuatumione	Cell viability	*In vitro* SH-SY5Y and PC12 cell lines	**Increased** cell viability	**Decreased** induced cell damage	CP	*Hypericum monogynum* L., AP, MeOH	[69]

Compound Class	Compound	Measurement	Method	Outcome		Therapeutic Application	Species, Plant Part and Extract	Ref #
**Acylphloroglucinol**	Japonicin A	Cell viability	*In vitro* OVCAR-3	**Decreased** cell viability (selective toxicity)	Cell viability%: 91.0±1.4 - 65.0±0.6% vs 58.0±7.9% paclitaxel control	AC	*Hypericum myrianthum* Cham. & Schltdl., AP, n-Hexane	[70]
**Acylphloroglucinol**	Uliginosin B	Cell viability	*In vitro* OVCAR-3	**Decreased** cell viability (selective toxicity)	Cell viability%: 81.0±1.0 - 66.0±0.8% vs 58.0±7.9% paclitaxel control	AC	*Hypericum myrianthum* Cham. & Schltdl., AP, n-Hexane	[70]
**Acylphloroglucinol**	Uliginosin B	Induced nociceptive behaviour	*In vivo* mouse model	**Decreased** painful behaviours<br>Ataxic effect		AD, AN	*Hypericum myrianthum* Cham. & Schltdl., AP, n-Hexane	[71]
**Acylphloroglucinol**	Olympicin A	Bacterial susceptibility	*In vitro* MDR/MR *Staphylococcus aureus*	Active against drug resistant *S. aureus* strains	MIC= 0.0005 - 0.001 µg/mL vs Norfloxacin MIC= 0.0005 - 0.256 µg/mL Vancomycin MIC= 0.00025 - 0.0005 µg/mL	AM	*Hypericum olympicum* L, -, -	[72]
**Acylphloroglucinol**	(S)-1-(2,4-Dihydroxy-6-(octyloxy)phenyl)-2-methylbutan-1-one	Bacterial susceptibility	*In vitro* MDR/MR *Staphylococcus aureus*	Active against drug resistant *S. aureus* strains<br>Olympicin A synthesised derivative	MIC = 0.00025 - 0.0005 µg/mL vs Norfloxacin MIC = 0.0005 - 0.256 µg/mL Vancomycin MIC = 0.00025 - 0.0005 µg/mL	AM	*Hypericum olympicum* L, -, -	[72]
**Acylphloroglucinol**	Olympicin A	Bacterial susceptibility	*In vitro* *S.aureus,* MRSA	Active against all tested strains	MIC = 2.9 - 1.45µM vs MIC = 1276 - 3.1µM controls	AM	*Hypericum olympicum* L. cf. *uniflorum*, AP, n-Hexane	[73]

Compound Class	Compound	Measurement	Method	Outcome		Therapeutic Application	Species, Plant Part and Extract	Ref #
**Acylphloroglucinol**	Hyperpatulol D	Cell migration Vimentin and E-cadherin expression	*In vitro* U2-OS cells	**Decreased** U2-OS migration	**Increased** E-cadherin expression **Decreased** Vimentin expression	AC	*Hypericum patulum* Thunb., Fl, EtOH	[74]
**Acylphloroglucinol**	Norhyperpalum B	Cell viability	*In vitro* Hep3B, HepG2, SMMC-7721 and Huh-7 cell lines	**Decreased** cell viability (selective cytotoxicity)	**Induced** S phase cell cycle arrest, apoptosis	AC	*Hypericum patulum* Thunb., L, EtOH	[75]
**Acylphloroglucinol**	Hypaluton A	Cell proliferation	*In vitro* B lymphocytes proliferation model	**Decreased** LPS induced B lymphocytes proliferation	IC_{50}= 6.86±0.72μM vs IC_{50}<1μM cyspin control	IM	*Hypericum patulum* Thunb., L, EtOH	[76]
**Acylphloroglucinol**	Hyperforatin L	AChE activity	*In vitro* PC12 cells	**Decreased** PC12 cells corticosterone induced cell damage	Acetylcholinesterase inhibition IC_{50}= 11.9 μM vs IC_{50}=0.2 ± 0.02 μM tacrine control	CP	*Hypericum perforatum* L., AP, EtOH	[77]

Compound Class	Compound	Measurement	Method	Outcome		Therapeutic Application	Species, Plant Part and Extract	Ref #
**Acylphloroglucinol**	Hyperforin	Expression modulation	*In vitro* SH-SY5Y cells	**Regulated** gene expression, *vs* citalopram control	**Suppressed** FKBP5 mRNA induced increase expression, CREB induced decrease expression **Increased** CREB expression**,** GRIK4 mRNA expression, VEGF mRNA expression **Decreased** ARRB2 induced decrease expression	AD	*Hypericum perforatum* L., -, -	[15]
**Acylphloroglucinol**	Hyperforatone E	AChE and BACE1 activity	*In vitro*	**Decreased** AChE and BACE1 activity	AChE inhibition IC_{50}= 7.9±0.7 μM vs IC_{50}=0.3±0.006 μM Tacrine control BACE1 inhibition rate 50.3±0.2% vs 40.0±3.8% EGCG control	Alz	*Hypericum perforatum* L., L, S, EtOH	[78]
**Acylphloroglucinol**	Hyperforatone J	AChE and BACE1 activity	*In vitro*	**Decreased** AChE and BACE1 activity	AChE inhibition IC_{50}= 9.2±0.8 μM vs IC_{50}=0.3±0.006 μM Tacrine control BACE1 inhibition rate 34.3±2.6% vs 40.0±3.8% EGCG control	Alz	*Hypericum perforatum* L., L, S, EtOH	[78]
**Acylphloroglucinol**	Hyperforatone H	AChE and BACE1 activity	*In vitro*	**Decreased** AChE and BACE1 activity	AChE inhibition IC_{50}= 7.6±0.3 μM vs IC_{50}=0.3±0.006 μM Tacrine control BACE1 inhibition rate 47.2±3.6% vs 40.0±3.8% EGCG control	Alz	*Hypericum perforatum* L., L, S, EtOH	[78]

Compound Class	Compound	Measurement	Method	Outcome		Therapeutic Application	Species, Plant Part and Extract	Ref #
**Acylphloroglucinol**	Hyperforatone O	AChE and BACE1 activity	*In vitro*	**Decreased** AChE and BACE1 activity	AChE inhibition IC_{50}= 6.9±0.3 μM vs IC_{50}=0.3±0.006 μM Tacrine control BACE1 inhibition rate 34.6±3.2% vs 40.0±3.8% EGCG control	Alz	*Hypericum perforatum* L., L, S, EtOH	[78]
**Acylphloroglucinol**	Hyperforatin B	AChE activity	*In vitro*	**Decreased** AChE activity	IC_{50}= 8.83±0.599 μM vs IC_{50}= 0.27±0.013 μM Tacrine control	Alz, CP	*Hypericum perforatum* L., L, S, EtOH	[79]
**Acylphloroglucinol**	Hyperforatin D	AChE activity	*In vitro*	**Decreased** AChE activity	IC_{50}= 7.17±0.134 μM vs IC_{50}= 0.27±0.013 μM Tacrine control	Alz, CP	*Hypericum perforatum* L., L, S, EtOH	[79]
**Acylphloroglucinol**	15-epi-Hyperforatin D	AChE activity	*In vitro*	**Decreased** AChE activity	IC_{50}= 3.98±0.924 μM vs IC_{50}= 0.27±0.013 μM Tacrine control	Alz, CP	*Hypericum perforatum* L., L, S, EtOH	[79]
**Acylphloroglucinol**	32-epi-Hyperforatin E	AChE activity	*In vitro*	**Decreased** AChE activity	IC_{50}= 9.13±1.022 μM vs IC_{50}= 0.27±0.013 μM Tacrine control	Alz, CP	*Hypericum perforatum* L., L, S, EtOH	[79]

Compound Class	Compound	Measurement	Method	Outcome		Therapeutic Application	Species, Plant Part and Extract	Ref #
**Acylphloroglucinol**	Hyperforatin F	AChE activity, Cell viability	*In vitro* SMMC7721 cells	**Decreased** AChE activity, cell viability	IC_{50}= 8.75±0.521 µM vs IC_{50}= 0.27±0.013 µM Tacrine control (AChE inhibition) IC_{50}= 10.0µM vs IC_{50}=8.98µM cis-platin control vs IC_{50}=<0.008 µM Taxol control (SMMC7721 viability)	Alz,CP, AC	*Hypericum perforatum* L., L, S, EtOH	[79]
**Acylphloroglucinol**	Hyperforatin I	Cell viability	*In vitro* SMMC7721 cells	**Decreased** AChE activity, cell viability	IC_{50}= 9.13µM vs IC_{50}=8.98µM cis-platin control vs IC_{50}=<0.008 µM Taxol control	AC CP	*Hypericum perforatum* L., L, S, EtOH	[79]
**Acylphloroglucinol**	Hyperfol F	AChE activity	*In vitro*	**Decreased** AChE activity	IC_{50}= 20.32±0.68 µM vs IC_{50}=0.7±0.02nM Tacrine control	Alz	*Hypericum perforatum* L., AP, MeOH	[80]
**Acylphloroglucinol**	Uralione K	AChE activity	*In vitro*	**Decreased** AChE activity	IC_{50}= 27.37±1.21 µM vs IC_{50}=0.7±0.02nM Tacrine control	Alz	*Hypericum perforatum* L., AP, MeOH	[80]
**Acylphloroglucinol**	Hyperfol A	Cell viability	*In vitro* HEL and K562 cell lines	**Decreased** cell viability **Increased** apoptosis	IC_{50}= 6.19 - 15.01 µM vs IC_{50}=0.6 - 0.15µM Adriamycin control	AC	*Hypericum perforatum* L., AP, MeOH	[81]
**Acylphloroglucinol**	Hyperuralone E	Cell viability	*In vitro* HEL and K562 cell lines	**Decreased cell viability** **Increased** apoptosis	IC_{50}= 8.69 - 7.38 µM vs IC_{50}=0.6 - 0.15µM Adriamycin control	AC	*Hypericum perforatum* L., AP, MeOH	[81]

Compound Class	Compound	Measurement	Method	Outcome		Therapeutic Application	Species, Plant Part and Extract	Ref #
**Acylphloroglucinol**	Hyperforin	Cell viability, Antimicrobial activity	*In vitro Toxoplasma gondii* infection model, sulforhodamine B Vero cells cytocoxicity assay	**Decreased** *T. gondii* growth, inflammatory response **Increased** cell viability		CP, AP	*Hypericum perforatum* L., WP, MeOH	[82]
**Acylphloroglucinol**	Hyperformitin A	Cell viability	*In vitro* LPS-induced B lymphocyte proliferation model, *In vitro* corticosterone induced PC12 cell injury model	**Decreased** B lymphocyte proliferation **Increased** PC12 cell viability	IC_{50}= 9.7µM *vs* IC_{50}=<1µM cyclosporine A control	CP	*Hypericum perforatum* L., S, L, EtOH	[83]
**Acylphloroglucinol**	Hyperformitin C	Cell viability	*In vitro* LPS-induced B lymphocyte proliferation	**Decreased** B lymphocyte proliferation	IC_{50}= 4.3µM *vs* IC_{50}=<1µM cyclosporine A control	CP	*Hypericum perforatum* L., S, L, EtOH	[83]
**Acylphloroglucinol**	Hyperformitin D	Cell viability	*In vitro* LPS-induced B lymphocyte proliferation	**Decreased** B lymphocyte proliferation	IC_{50}= 9.3µM *vs* IC_{50}=<1µM cyclosporine A control	CP	*Hypericum perforatum* L., S, L, EtOH	[83]
**Acylphloroglucinol**	Hyperformitin E	Cell viability	*In vitro* LPS-induced B lymphocyte proliferation	**Decreased** B lymphocyte proliferation	IC_{50}= 4.1µM *vs* IC_{50}=<1µM cyclosporine A control	CP	*Hypericum perforatum* L., S, L, EtOH	[83]

Compound Class	Compound	Measurement	Method	Outcome		Therapeutic Application	Species, Plant Part and Extract	Ref #
**Acylphloroglucinol**	Hyperformitin G	Cell viability	*In vitro* LPS-induced B lymphocyte proliferation	**Decreased** B lymphocyte proliferation	IC_{50}= 9.2µM *vs* IC_{50}=<1µM cyclosporine A control	CP	*Hypericum perforatum* L., S, L, EtOH	[83]
**Acylphloroglucinol**	Hyperformitin K	Cell viability	*In vitro* LPS-induced B lymphocyte proliferation	**Decreased** B lymphocyte proliferation	IC_{50}= 8.8µM *vs* IC_{50}=<1µM cyclosporine A control	CP	*Hypericum perforatum* L., S, L, EtOH	[83]
**Acylphloroglucinol**	Hyperformitin L	Cell viability	*In vitro* LPS-induced B lymphocyte proliferation model, *In vitro* corticosterone induced PC12 cell injury model	**Increased** PC12 cell viability		CP	*Hypericum perforatum* L., S, L, EtOH	[83]
**Acylphloroglucinol**	Hyperformitin M	Cell viability	*In vitro* LPS-induced B lymphocyte proliferation model, *In vitro* corticosterone induced PC12 cell injury model	**Increased** PC12 cell viability		CP	*Hypericum perforatum* L., S, L, EtOH	[83]
**Acylphloroglucinol**	Hyperforone F	Cell viability	*In vivo* rat model	**Decreased** tau phosphorilation and Aβ production	PP2A and BACE1 gene expression modulation	Alz	*Hypericum perforatum* L., AP, EtOH	[84]

Compound Class	Compound	Measurement	Method	Outcome		Therapeutic Application	Species, Plant Part and Extract	Ref #
**Acylphloroglucinol**	Uliginosin B	Nociceptive behaviours and motor coordination	*In vivo mouse model*	**Improved** antinociceptive and motor coordination scores		AN	*Hypericum polyanthemum* Klotzsch ex Reichardt, AP, n-Hexane	[71]
**Acylphloroglucinol**	Uliginosin B	Protozoal susceptibility	*In vitro Trichomonas vaginalis*	**Decreased** *T. vaginalis* cell viability	IC_{50}=121.96μM	AP	*Hypericum polyanthemum* Klotzsch ex Reichardt, AP, Supercritical CO2	[85]
**Acylphloroglucinol**	Hyperprin A	Cell proliferation	*In vitro* MV-4-11 cell lines	**Decreased** cell proliferation	IC_{50}= 15.35±1.86μM vs IC_{50}=9.68±0.86μM CC-90011 control	AC	*Hypericum przewalskii* Maxim., -, -	[86]
**Acylphloroglucinol**	Uraloidin A	Oxidative stress	*In vitro* murine peritoneal macrophages	**Decreased** LPS-induced NO production		CP	*Hypericum pseudohenryi* N.Robson, AP, EtOH	[87]
**Acylphloroglucinol**	Hyperisampsin A	Cell viability, HIV replication	*In vitro* HL-60, SMMC-7721, A-549, MCF-7, SW-480 cells	**Decreased** cell viability (selective activity) **Decreased** HIV replication	IC_{50} = 28.18 - 10.12μM vs 15.86 - 1.17μM Cysplatin control EC_{50}=2.97μM vs IC_{50}=0.0014 Zidovudine control	AC AV	*Hypericum sampsonii* Hance, AP, EtOH	[88]
**Acylphloroglucinol**	Hyperisampsin B	Cell viability	*In vitro* SMMC-7721, A-549, MCF-7 cells	**Decreased** cell viability (selective activity)	IC_{50} = 39.58 - 27.07μM vs 15.86 - 6.43μM Cysplatin control	AC	*Hypericum sampsonii* Hance, AP, EtOH	[88]

Compound Class	Compound	Measurement	Method	Outcome		Therapeutic Application	Species, Plant Part and Extract	Ref #
**Acylphloroglucinol**	Hyperisampsin C	Cell viability	*In vitro* HL-60, SMMC-7721, A-549, MCF-7, SW-480 cells	**Decreased** cell viability (selective toxicity)	IC_{50} = 24.49 - 9.49µM vs 15.86 - 1.17µM Cysplatin control	AC	*Hypericum sampsonii* Hance, AP, EtOH	[88]
**Acylphloroglucinol**	Hyperisampsin D	Cell viability, HIV replication	*In vitro* HL-60, SMMC-7721, A-549, MCF-7, SW-480 cells	**Decreased** cell viability (selective toxicity) **Decreased** HIV replication	IC_{50} = 15.72 - 5.95µM vs 15.86 - 1.17µM Cysplatin control EC_{50}=0.97µM vs IC_{50}=0.0014 Zidovudine control	AC AV	*Hypericum sampsonii* Hance, AP, EtOH	[88]
**Acylphloroglucinol**	Hyperisampsin E	Cell viability	*In vitro* HL-60, SMMC-7721, A-549, MCF-7, SW-480 cells	**Decreased** cell viability (selective toxicity)	IC_{50} = 34.29 - 10.02µM vs 15.86 - 1.17µM Cysplatin control	AC	*Hypericum sampsonii* Hance, AP, EtOH	[88]
**Acylphloroglucinol**	Hyperisampsin F	Cell viability	*In vitro* HL-60, SMMC-7721, A-549, MCF-7 cells	**Decreased** cell viability (selective toxicity)	IC_{50} = 31.30 - 13.14µM vs 15.86 - 1.17µM Cysplatin control	AC	*Hypericum sampsonii* Hance, AP, EtOH	[88]
**Acylphloroglucinol**	Hyperisampsin G	Cell viability	*In vitro* HL-60, SMMC-7721, A-549, MCF-7, SW-480 cells	**Decreased** cell viability (selective toxicity)	IC_{50} = 26.78 - 11.87µM vs 15.86 - 1.17µM Cysplatin control	AC	*Hypericum sampsonii* Hance, AP, EtOH	[88]
**Acylphloroglucinol**	Hypersampsone A	Expression modulation	*In vitro* HepG2/ADR and MCF-7/ADR cancer cell lines	**Decreased** multidrug resistance activity		IM, AC	*Hypericum sampsonii* Hance, -, -	[89]

Compound Class	Compound	Measurement	Method	Outcome		Therapeutic Application	Species, Plant Part and Extract	Ref #
**Acylphloroglucinol**	Hyperhexanone F	Expression modulation	*In vitro* HepG2/ADR and MCF-7/ADR cancer cell lines	**Decreased** multidrug resistance activity		IM, AC	*Hypericum sampsonii* Hance, -, -	[89]
**Acylphloroglucinol**	Hypermongone C	Fibroblast migration and proliferation Endothelial cells tube formation Cytokine expression	*In vitro* HUVEC, HDF cells	**Increased** fibroblast proliferation and migration, angiogenesis **Decreased** pro-inflammatory cytokines expression	**Decreased** IL-6 and TNFα expression **Increased** VEGF growth factor production	S	*Hypericum scabrum* L., AP, Hexane	[90]
**Acylphloroglucinol**	*Hypericum*oxides A-N	Cell viability Serotonin reuptake	*In vitro* HL-7702 cells	**Decreased** serotonin reuptake **Increased** cell viability	**Increased** cell survival rate 64- 65% (*Hypericum*oxide D, M) vs 74% bicycliol control **Decreased** Serotonin reuptake 30.9-51.0% (*Hypericum*oxide A-E, G-I, M-N) vs 94.7% duloxetine control	AD, CP	*Hypericum scabrum* L., AP, EtOH	[91]
**Acylphloroglucinol**	Hypercohin B	Cell viability	*In vitro* HL-7702 cells	**Increased** cell viability **Decreased** cell damage	**Increased** cell survival rate 65% (*Hypericum*oxide D, M) vs 74% bicycliol control	CP	*Hypericum scabrum* L., AP, EtOH	[91]
**Acylphloroglucinol**	Hyperibrin G	Cell viability	*In vitro* HepG2 cells	**Increased** cell viability	Cell viability (%): 56.53±4.74% vs 54.8±1.99% Bicyclol control	CP	*Hypericum scabrum* L., AP, EtOH	[92]

Compound Class	Compound	Measurement	Method	Outcome		Therapeutic Application	Species, Plant Part and Extract	Ref #
**Acylphloroglucinol**	(2R,4R,6S)-2-benzoyl-3,3-dimethyl-4,6-bis(3-methylbut-2-en-1-yl)cyclohexan-1-one	Cell viability	*In vitro* HepG2 cells	**Increased** cell viability	Cell viability (%): 61.96±1.83% vs 54.8±1.99% Bicyclol control	CP	*Hypericum scabrum* L., AP, EtOH	[92]
**Acylphloroglucinol**	Sampsonione N	Cell viability	*In vitro* HepG2 cells	**Increased** cell viability	Cell viability (%): 59.97±1.07% vs 54.8±1.99% Bicyclol control	CP	*Hypericum scabrum* L., AP, EtOH	[92]
**Acylphloroglucinol**	7-epiclusianone	Cell viability	*In vitro* HepG2 cells	**Increased** cell viability	Cell viability (%): 58.62±3.28% vs 54.8±1.99% Bicyclol control	CP	*Hypericum scabrum* L., AP, EtOH	[92]
**Acylphloroglucinol**	Hyperibrins A	Cell viability	*In vitro* SK-N-SH cells	**Increased** cell viability	SK-N-SH cell viability 71.2±3.5 % vs 72.6±1.5% Resveratrol control	CP	*Hypericum scabrum* L., AP, EtOH	[93]
**Acylphloroglucinol**	Hyperibrins C	Cell viability	*In vitro* SK-N-SH, HepG2 cells	**Increased** cell viability	SK-N-SH cell viability 81.3±4.2 % vs 72.6±1.5% Resveratrol control<br>HepG2 cell viability 36.0±2.1 % vs 43.2±2.4% Bicyclol control	CP	*Hypericum scabrum* L., AP, EtOH	[93]

Compound Class	Compound	Measurement	Method	Outcome		Therapeutic Application	Species, Plant Part and Extract	Ref #
**Acylphloroglucinol**	Hyperibrins D	Cell viability	*In vitro*<br>SK-N-SH, HepG2 cells	**Increased** cell viability	SK-N-SH cell viability 71.3±0.3 % vs 72.6±1.5% Resveratrol control<br>HepG2 cell viability 35.2±3.6 % vs 43.2±2.4% Bicyclol control	CP	*Hypericum scabrum* L., AP, EtOH	[93]
**Acylphloroglucinol**	Hyperscabrone C	Cell viability	*In vitro*<br>HepG2 cells	**Increased** cell viability	HepG2 cell viability 33.7±1.1 % vs 43.2±2.4% Bicyclol control	CP	*Hypericum scabrum* L., AP, EtOH	[94]
**Acylphloroglucinol**	Hyperscabrone D	Cell viability	*In vitro*<br>SK-N-SH, HepG2 cells	**Increased** cell viability	SK-N-SH cell viability 70.8±0.5 % vs 72.6±1.5% Resveratrol control<br>HepG2 cell viability 47.0±5.4 % vs 43.2±2.4% Bicyclol control	CP	*Hypericum scabrum* L., AP, EtOH	[94]
**Acylphloroglucinol**	Hyperscabrone E	Cell viability	*In vitro*<br>SK-N-SH cells	**Increased** cell viability	SK-N-SH cell viability 73.1±3.7 % vs 72.6±1.5% Resveratrol control	CP	*Hypericum scabrum* L., AP, EtOH	[94]
**Acylphloroglucinol**	Hyperscabrone F	Cell viability	*In vitro*<br>SK-N-SH cells	**Increased** cell viability	SK-N-SH cell viability 73.5±2.9 % vs 72.6±1.5% Resveratrol control	CP	*Hypericum scabrum* L., AP, EtOH	[94]

Compound Class	Compound	Measurement	Method	Outcome		Therapeutic Application	Species, Plant Part and Extract	Ref #
**Acylphloroglucinol**	Hyperscabrone G	Cell viability	*In vitro* SK-N-SH, HepG2 cells	**Increased** cell viability	SK-N-SH cell viability 72.1±2.3 % vs 72.6±1.5% Resveratrol control HepG2 cell viability 55.3±2.1 % vs 43.2±2.4% Bicyclol control	CP	*Hypericum scabrum* L., AP, EtOH	[94]
**Acylphloroglucinol**	Hyperscabrone I	Cell viability	*In vitro* HepG2 cells	**Increased** cell viability	HepG2 cell viability 50.9±3.6 % vs 43.2±2.4% Bicyclol control	CP	*Hypericum scabrum* L., AP, EtOH	[94]
**Acylphloroglucinol**	Hyperibone J	Cell viability	*In vitro* HepG2 cells	**Increased** cell viability	HepG2 cell viability 47.6±2.1 % vs 43.2±2.4% Bicyclol control	CP	*Hypericum scabrum* L., AP, EtOH	[94]
**Acylphloroglucinol**	8-hydroxyhyperforin 8,1-hemiacetal	Cell viability	*In vitro* HepG2 cells	**Increased** cell viability	HepG2 cell viability 39.3±3.8 % vs 43.2±2.4% Bicyclol control	CP	*Hypericum scabrum* L., AP, EtOH	[94]
**Acylphloroglucinol**	hypermongone G	Cell viability	*In vitro* SK-N-SH cells	**Increased** cell viability	SK-N-SH cell viability 71.6±4.7 % vs 72.6±1.5% Resveratrol control	CP	*Hypericum scabrum* L., AP, EtOH	[94]
**Acylphloroglucinol**	hypermongone H	Cell viability	*In vitro* HepG2 cells	**Increased** cell viability	HepG2 cell viability 45.8±1.9 % vs 43.2±2.4% Bicyclol control	CP	*Hypericum scabrum* L., AP, EtOH	[94]

Compound Class	Compound	Measurement	Method	Outcome		Therapeutic Application	Species, Plant Part and Extract	Ref #
**Acylphloroglucinol**	hyperibone A	Cell viability	*In vitro*<br>SK-N-SH cells	**Increased** cell viability	SK-N-SH cell viability 83.0±3.6 % vs 72.6±1.5% Resveratrol control	CP	*Hypericum scabrum* L., AP, EtOH	[94]
**Acylphloroglucinol**	hyperibone B	Cell viability	*In vitro*<br>HepG2 cells	**Increased** cell viability	HepG2 cell viability 36.7±2.9 % vs 43.2±2.4% Bicyclol control	CP	*Hypericum scabrum* L., AP, EtOH	[94]
**Acylphloroglucinol**	hypermongone D	Cell viability	*In vitro*<br>SK-N-SH cells	**Increased** cell viability	SK-N-SH cell viability 75.4±4.2 % vs 72.6±1.5% Resveratrol control	CP	*Hypericum scabrum* L., AP, EtOH	[94]
**Acylphloroglucinol**	yezo'otogirin C	Cell viability	*In vitro*<br>HepG2 cells	**Increased** cell viability	HepG2 cell viability 40.6±5.3 % vs 43.2±2.4% Bicyclol control	CP	*Hypericum scabrum* L., AP, EtOH	[94]
**Acylphloroglucinol**	hyperibone K	Cell viability	*In vitro*<br>SK-N-SH, HepG2 cells	**Increased** cell viability	SK-N-SH cell viability 73.1±1.8 % vs 72.6±1.5% Resveratrol control<br>HepG2 cell viability 64.0±8.0 % vs 43.2±2.4% Bicyclol control	CP	*Hypericum scabrum* L., AP, EtOH	[94]

Compound Class	Compound	Measurement	Method	Outcome		Therapeutic Application	Species, Plant Part and Extract	Ref #
**Acylphloroglucinol**	Unnamed compound	Antimicrobial activity, cell viability	*In vitro* *Trypanosoma brucei rhodesiense*, *Plasmodium falciparum*; L6 rat mioblast cells	**Decreased** protozoal activity; L6 cell viability		AM	*Hypericum scabrum* L., AP, n-Hexane	[95]
**Acylphloroglucinol**	Hyperforin	Cell viability, Oxidative stress	*In vitro* PC12, SH-SY5Y cells	**Increased** PC12 cell viability **Decreased** apopotosis, oxidative stress	**Decreased**: LDH, ROS, MDA - vs Aluminum control **Increased**: SOD, GSH-Px - vs Aluminum control **Suppressed**: Aluminum induced MMP reduction, Cytochrome C release, Caspase-3 activation, Bcl-2 downregulation, Bax upregulation	CP	*Hypericum* spp., -, -	[96]
**Acylphloroglucinol**	Japonicin A	Cell proliferation	*In vitro* HaCaT, MRC5 and MSC	**Increased** HaCaT proliferation	5 µM (110% *vs* blank control)	S	*Hypericum* spp., AP, n-Hexane	[97]

Compound Class	Compound	Measurement	Method	Outcome		Therapeutic Application	Species, Plant Part and Extract	Ref #
**Acylphloroglucinol**	Uliginosin B	Cell proliferation	*In vitro* HaCaT, MRC5 and MSC	**Increased** MSC and MRC5 cell viability	5 µM (116% vs blank control) MSC cells<br>10 µM (129% vs blank control) MSC cells<br>1 µM (125% vs blank control) MRC5<br>5 µM (152.5% vs blank control) MRC5<br>10 µM (151.1% vs blank control) MRC5	S	*Hypericum* spp., AP, n-Hexane	[97]
**Acylphloroglucinol**	Hyperforin	Expression modulation Microglial activation Infarct volume	*In vivo* Ischemic Mouse model	**Reduced** infarct volume<br>**Inhibited** IL-17A activation of microglia	**Decreased** mRNA and protein expression, round microglia, CD16, CD11b, CD32, iNOS, TNFα<br>**Increased** IL10, Arg-1, TGF β, CD206, YM1, ramified microglia<br>vs saline solution	CP	*Hypericum* spp., -, -	[98]
**Acylphloroglucinol**	Hyperforin	JAK1 activity	*In vitro*	**Decreased** JAK1 activity		AI	*Hypericum* spp., -, -	[99]
**Acylphloroglucinol**	Hyperforin	STAT-1 and NF-κB activity	*In vitro* INS-1E cells	**Decreased** cytokine induced apoptosis	**Decreased** cytokine induced STAT-1 activation	ADb	*Hypericum* spp., -, -	[100]
**Acylphloroglucinol**	Hyperforin	Cell viability, Genotoxicity	*In vitro* HepG2 cells	**Decreased** HepG2 viability; gene mutations; DNA damage	Antigenotoxicity against zeocin	AC<br>CP	*Hypericum* spp., -, -	[19]

Compound Class	Compound	Measurement	Method	Outcome		Therapeutic Application	Species, Plant Part and Extract	Ref #
**Acylphloroglucinol**	Hyperforin	Al-induced β-amyloid formation and tau hyperphosphorylation	*In vitro* PC12 cells	**Decreased** β-amyloid formation	**Decreased** APP, BACE1, PS1 expression **Increased** sAPPα, ADAM9/10/17 expression, Tau phosphorylation, GSK-3β phosphorylation	Alz	*Hypericum* spp., -, -	[101]
**Acylphloroglucinol**	Hyperforin	Cell viability CYP1A2 and CYP2D6 expression	*In vitro* WRL-68, HepG2	**Increased** CYP1A2 (HepG2, WRL-68), CYP2D6 (HepG2) expression **Decreased** CYP2D6 (HepaRG, WRL-68) expression		Int	*Hypericum* spp., -, -	[102]
**Acylphloroglucinol**	Hyperforin	STAT-1 and NF-κB activity	*In vitro* INS-1E cells	**Decreased** cytokine induced apoptosis, expression of pro-inflammatory genes, insulin release suppression	**Decreased** cytokine induced STAT-1, NF-κB p65 subunit, IKK, MAPK activation **Decreased** cytokine induced CXCL9, CXCL10, MHC II, ICAM1, COX2, BH3, Bak, CHOP, PTPN2 expression **Decreased** Pdx1, Nkx2.2, Nkx6.1, Bcl-2 cytokine induced downregulation	ADb	*Hypericum* spp., -, -	[103]
**Acylphloroglucinol**	Hyperforin	Cell proliferation	*In vivo* mouse model	**Decreased** autoimmune encephalomyelitis severity	**Decreased** T-cell proliferation, demyelination	CP	*Hypericum* spp., -, -	[104]

**Compound Class**	**Compound**	**Measurement**	**Method**	**Outcome**		**Therapeutic Application**	**Species, Plant Part and Extract**	**Ref #**
**Acylphloroglucinol**	Hyperforin	Wound healing	*In vitro* HaCaT keratinocyte cells	**Improved** wound healing	**Increased** intracellular Ca2+, ATP release, TRPC6 expression	S	*Hypericum* spp., -, -	[105]
**Acylphloroglucinol**	Hyperforin	Expression modulation	*In vitro* Human and rat β-cells	**Decreased** cytokine induced insulin release suppression, pro-inflammatory genes expression, nitrites production, apoptosis, STAT-1 and NF-kB activation	**Decreased** iNOS, CXCL9, CXCL10, COX2, NO expression	ADb	*Hypericum* spp., -, -	[106]
**Acylphloroglucinol**	Hyperforin	Expression modulation	*In vitro, Ex vivo*	Tissue specific TRPC6 activation	**Increased** TrkB, p-TrkB, CREB, p-CREB expression	CP	*Hypericum* spp., -, -	[107]
**Acylphloroglucinol**	Hyperforin	Cell Viability	*Ex vivo* MEC-1 cells	**Increased** apoptosis	**Increased** Noxa expression, Mcl-1/Bak complex dissociation, Bak activation, Noxa/Mcl-1 association **Decreased** Proteasome activity	AC	*Hypericum* spp., -, -	[22]
**Acylphloroglucinol**	Hyperforin	Cell maturation	*In vitro/ Ex vivo* Central glia-4 cells	**Increases** oligodendrocytes maturation, mitochondrial function of differentiating CG-4 cells and NS/PCs	**Decreased** mitochondrial toxin induced cytotoxicity, CG-4 rotenone induced ATP depletion	AD	*Hypericum* spp., -, -	[108]

Compound Class	Compound	Measurement	Method	Outcome		Therapeutic Application	Species, Plant Part and Extract	Ref #
**Acylphloroglucinol**	Hyperforin	Cell viability	*In vitro* K562 cells	**Decreased** cell viability **Increased** apoptosis	**Decreased** mitochondrial transmembrane potential **Increased C**ytochrome C, Casp-3, Casp-8, Casp-9 activation, PARP cleavage	AC	*Hypericum* spp., -, -	[24]
**Acylphloroglucinol**	Tetrahydrohyperforin (d)	Aβ neurotoxicity and behavioral impairments	*In vitro* H4 neuroglioma cells *Ex vivo/in vivo* mouse model	**Improved** memory and **decreased** synaptic plasticity (*in vivo*)	**Decreased** tau hyperphosphorylation, astrogliosis, total fibrillar/oligomeric forms of Aβ, long term potentiation, inactive GSK-3β (in vivo) **Decreased** Aβ precursor protein proteolysis, AICDγ levels;	Alz	*Hypericum* spp., -, -	[109]
**Acylphloroglucinol**	Hypersubone A	Cell viability	*In vitro* HepG2, Eca109, HeLa and A549 cells	**Decreased** cell viability (selective activity)	IC_{50} = >50 - 17.74μM vs IC_{50}= 21.02 - 8.04μM etoposide control	AC	*Hypericum subsessile* N.Robson, AP, MeOH	[110]
**Acylphloroglucinol**	Hypersubone B	Cell viability	*In vitro* HepG2, Eca109, HeLa and A549 cells	**Decreased** cell viability (selective activity)	IC_{50} = 7.52 - 0.07μM vs IC_{50}= 21.02 - 8.04μM etoposide control	AC	*Hypericum subsessile* N.Robson, AP, MeOH	[110]
**Acylphloroglucinol**	Hypersubone C	Cell viability	*In vitro* HepG2, Eca109, HeLa and A549 cells	**Decreased** cell viability (selective activity)	IC_{50} = 17.23 - 6.71μM vs IC_{50}= 21.02 - 8.04μM etoposide control	AC	*Hypericum subsessile* N.Robson, AP, MeOH	[110]

Compound Class	Compound	Measurement	Method	Outcome		Therapeutic Application	Species, Plant Part and Extract	Ref #
**Acylphloroglucinol**	Hypersubone D	Adipogenesis	*In vitro* 3T3-L1 cells	**Decreased** lipid acumulation in preadipocytes	Significant effect *vs* LiCl control	Ad.In	*Hypericum subsessile* N.Robson, AP, MeOH	[111]
**Acylphloroglucinol**	Hypersubone E	Adipogenesis	*In vitro* 3T3-L1 cells	**Decreased** lipid acumulation in preadipocytes	Significant effect *vs* LiCl control	Ad.In	*Hypericum subsessile* N.Robson, AP, MeOH	[111]
**Acylphloroglucinol**	Hypersubone H	Adipogenesis	*In vitro* 3T3-L1 cells	**Decreased** lipid acumulation in preadipocytes	Significant effect *vs* LiCl control	Ad.In	*Hypericum subsessile* N.Robson, AP, MeOH	[111]
**Acylphloroglucinol**	Hypersampsone P	Adipogenesis	*In vitro* 3T3-L1 cells	**Decreased** adipocyte differentiation	**Decreased** PPARγ and FABP4 expression	Ad.In	*Hypericum subsessile* N.Robson, -, -	[112]
**Acylphloroglucinol**	Uralione A	Cell viability	*In vitro* PC12 cells	**Increased** cell viability	Cell viability : 61.4±1.07% - 91.5±0.39% vs 59.1±0.12% - 77.7±0.31 % fluoxetine control	CP	*Hypericum uralum* Buch.-Ham. ex D.Don, WP, EtOH	[113]
**Acylphloroglucinol**	Uralione B	Cell viability	*In vitro* PC12 cells	**Increased** cell viability	Cell viability : 65.9±0.68% - 80.8±0.17% vs 59.1±0.12% - 77.7±0.31 % fluoxetine control	CP	*Hypericum uralum* Buch.-Ham. ex D.Don, WP, EtOH	[113]

Compound Class	Compound	Measurement	Method	Outcome		Therapeutic Application	Species, Plant Part and Extract	Ref #
**Acylphloroglucinol**	Uralione C	Cell viability	*In vitro* PC12 cells	**Increased** cell viability	Cell viability : 68.9±0.15% - 86.8±0.20% vs 59.1±0.12% - 77.7±0.31 % fluoxetine control	CP	*Hypericum uralum* Buch.-Ham. ex D.Don, WP, EtOH	[113]
**Acylphloroglucinol**	Uralione D	Cell viability	*In vitro* PC12 cells	**Increased** cell viability	Cell viability : 72.5±0.43% - 80.9±0.32% vs 59.1±0.12% - 77.7±0.31 % fluoxetine control	CP	*Hypericum uralum* Buch.-Ham. ex D.Don, WP, EtOH	[113]
**Acylphloroglucinol**	Uralione E	Cell viability	*In vitro* PC12 cells	**Increased** cell viability	Cell viability : 68.1±0.39% - 86.6±0.36% vs 59.1±0.12% - 77.7±0.31 % fluoxetine control	CP	*Hypericum uralum* Buch.-Ham. ex D.Don, WP, EtOH	[113]
**Acylphloroglucinol**	Uralione F	Cell viability	*In vitro* PC12 cells	**Increased** cell viability	Cell viability : 74.1±0.72% - 81.8±0.40% vs 59.1±0.12% - 77.7±0.31 % fluoxetine control	CP	*Hypericum uralum* Buch.-Ham. ex D.Don, WP, EtOH	[113]
**Acylphloroglucinol**	Uralione G	Cell viability	*In vitro* PC12 cells	**Increased** cell viability	Cell viability : 68.1±0.44% - 78.6±0.29% vs 59.1±0.12% - 77.7±0.31 % fluoxetine control	CP	*Hypericum uralum* Buch.-Ham. ex D.Don, WP, EtOH	[113]
**Acylphloroglucinol**	Uralione H	Cell viability	*In vitro* PC12 cells	**Increased** cell viability	Cell viability : 81.6±0.20% - 84.2±0.24% vs 59.1±0.12% - 77.7±0.31 % fluoxetine control	CP	*Hypericum uralum* Buch.-Ham. ex D.Don, WP, EtOH	[113]

Compound Class	Compound	Measurement	Method	Outcome		Therapeutic Application	Species, Plant Part and Extract	Ref #
**Acylphloroglucinol**	Uralione J	Cell viability	*In vitro* PC12 cells	**Increased** cell viability	Cell viability : 72.9±0.43% - 93.5±0.14% vs 59.1±0.12% - 77.7±0.31 % fluoxetine control	CP	*Hypericum uralum* Buch.-Ham. ex D.Don, WP, EtOH	[113]
**Acylphloroglucinol**	Uralione K	Cell viability	*In vitro* PC12 cells	**Increased** cell viability	Cell viability : 71.5±0.75% - 81.1±0.12% vs 59.1±0.12% - 77.7±0.31 % fluoxetine control	CP	*Hypericum uralum* Buch.-Ham. ex D.Don, WP, EtOH	[113]
**Acylphloroglucinol**	Uralodin A	Stress-induced learning and memory deficits	*In vivo* mouse model	**Decreased** immobility time in FST and TST *vs* fluoxetine control		AD	*Hypericum uralum* Buch.-Ham. ex D.Don, WP, EtOH	[113]
**Acylphloroglucinol**	Hyperuralone C	AChE activity	*In vitro*	AChE inhibition	IC_{50}= 9.6 µM	Alz	*Hypericum uralum* Buch.-Ham. ex D.Don, AP, MeOH	[114]
**Acylphloroglucinol**	Hyperuralone D	AChE activity	*In vitro*	AChE inhibition	IC_{50}= 7.1 µM	Alz	*Hypericum uralum* Buch.-Ham. ex D.Don, AP, MeOH	[114]
**Acylphloroglucinol**	Uralin D	Cell viability	*In vitro* Huvec cell lines	**Decreased** cell viability	IC_{50}= 26.3µM	AC	*Hypericum uralum* Buch.-Ham. ex D.Don, AP, EtOH	[115]

Compound Class	Compound	Measurement	Method	Outcome		Therapeutic Application	Species, Plant Part and Extract	Ref #
**Acylphloroglucinol**	Hyperlagarin C	Cell viability	*In vitro* Huvec cell lines	**Decreased** cell viability	IC_{50}= 31.1µM	AC	*Hypericum uralum* Buch.-Ham. ex D.Don, AP, EtOH	[115]
**Acylphloroglucinol**	Hyperlagarin A	Cell viability	*In vitro* Huvec cell lines	**Decreased** cell viability	IC_{50}= 21.9µM	AC	*Hypericum uralum* Buch.-Ham. ex D.Don, AP, EtOH	[115]
**Acylphloroglucinol**	Furohyperforin	Cell viability,Oxidative stress	*In vitro* Huvec cell lines	**Decreased** glucose induced cell damage **Increased** cell viability	Cell viability increase 57.2% *vs* 33.0% aspirin control	CP	*Hypericum uralum* Buch.-Ham. ex D.Don, AP, EtOH	[115]
**Acylphloroglucinol**	Oxepahyperforin	Cell viability,Oxidative stress	*In vitro* Huvec cell lines	**Decreased** glucose induced cell damage **Increased** cell viability	Cell viability increase 58.0% *vs* 33.0% aspirin control	CP	*Hypericum uralum* Buch.-Ham. ex D.Don, AP, EtOH	[115]
**Acylphloroglucinol**	Hyphenrone T	Cell viability,Oxidative stress	*In vitro* Huvec cell lines	**Decreased** glucose induced cell damage **Increased** cell viability	Cell viability increase 55.3% *vs* 33.0% aspirin control	CP	*Hypericum uralum* Buch.-Ham. ex D.Don, AP, EtOH	[115]
**Acylphloroglucinol**	Hypersonin A	Cell proliferation	*In vitro* mouse splenocytes model	**Decreased** anti-CD3/anti-CD28 monoclonal antibody induced cell proliferation		IM	*Hypericum wilsonii* N. Robson, S, L, EtOH	[116]

Compound Class	Compound	Measurement	Method	Outcome		Therapeutic Application	Species, Plant Part and Extract	Ref #
**Acylphloroglucinol**	Hyperwilsone I	NO production, gene expression	*In vitro* RAW264.7 cell lines	**Decreased** LPS induced NO production **Decreased** NF-κB p65 expression and proinflammatory cytokines production	IC_{50}= 9.12±0.47μM *vs* IC_{50}=5.50±0.36μM dexamethasone control	CP	*Hypericum wilsonii* N. Robson, S, L, EtOH	[117]
**Acylphloroglucinol**	Hyperwilsone J	NO production, gene expression	*In vitro* RAW264.7 cell lines	**Decreased** LPS induced NO production **Decreased** NF-κB p65 expression and proinflammatory cytokines production	IC_{50}= 6.15±0.11μM *vs* IC_{50}=5.50±0.36μM dexamethasone control	CP	*Hypericum wilsonii* N. Robson, S, L, EtOH	[117]
**Acylphloroglucinol**	Hyperwilsone E	Cell viability	*In vitro* SUDHL-4 and HL60 cancer cell lines	**Decreased** tumour cells viability		AC	*Hypericum wilsonii* N. Robson, S, L, EtOH	[117]
**Acylphloroglucinol**	Hyperwilsone K	Cell viability	*In vitro* SUDHL-4 and HL60 cancer cell lines	**Decreased** tumour cells viability		AC	*Hypericum wilsonii* N. Robson, S, L, EtOH	[117]
**Acylphloroglucinol**	Hyphenrone V	Cell viability	*In vitro* SUDHL-4 and HL60 cancer cell lines	**Decreased** tumour cells viability		AC	*Hypericum wilsonii* N. Robson, S, L, EtOH	[117]

Compound Class	Compound	Measurement	Method	Outcome		Therapeutic Application	Species, Plant Part and Extract	Ref #
**Acylphloroglucinol**	Yezo'otogirin E	Bacterial susceptibility	*In vitro*	Active against *Escherichia coli* and *Staphylococcus aureus*	MIC=4.0 µg/mL (*E.coli*) MIC=8.0 µg/mL (*S.aureus*)	AM	*Hypericum yezoense* Maxim., AP, MeOH	[118]
**Acylphloroglucinol**	Yojironin E	Bacterial susceptibility Cell viability	*In vitro Aspergillus niger, Candida albicans, Cryptococcus neoformans, Trichophytum mentagrophytes* P388, KB cells	Active against all tested strains (selective activity) **Decreased** tumour cells viability	IC_{50}=16 µg/mL (*Aspergillus niger*) IC_{50}=4 µg/mL (*Candida albicans*) IC_{50}=4 µg/mL (*Cryptococcus neoformans*) IC_{50}=4 µg/mL (*Trichophyton mentagrophytes*) Cytotoxicity: IC_{50}=3.7µg/mL (P388). IC_{50}=5.0µg/mL (KB)	AM, AC	*Hypericum yojiroanum* Tatew. & Koji Ito, WP, MeOH	[119]

Compound Class	Compound	Measurement	Method	Outcome		Therapeutic Application	Species, Plant Part and Extract	Ref #
**Acylphloroglucinol**	Yojironin A	Bacterial susceptibility Cell viability	*In vitro Aspergillus niger, Candida albicans, Cryptococcus neoformans, Trichophytum mentagrophytes, S. aureus, B. subtilis* L1210, KB tumour cell lines	Active against all tested strains **Decreased** tumour cells viability	IC_{50}=8 µg/mL (*Aspergillus niger*) IC_{50}=2 µg/mL (*Candida albicans*) IC_{50}=4 µg/mL (*Cryptococcus neoformans*) IC_{50}=2 µg/mL (*Trichophyton mentagrophytes*) IC_{50}=8 µg/mL (*S. aureus*) IC_{50}=4 µg/mL (*B. subtilis*) Cytotoxicity: IC_{50}=4.1µg/mL (L1210), IC_{50}=6.8µg/mL (KB)	AM, AC	*Hypericum yojiroanum* Tatew. & Koji Ito, WP, MeOH	[120]
**Acylphloroglucinol**	Hypatone A	Action potential	*In vitro* Cav3.1 low voltage-gated Ca2+ channels	Hypatone A is a Cav3.1 agonist, while biosynthetic analogues acted as antagonists	Activation of Ca v3.1	AEp, Sp.At	*Hypericum patulum* Thunb, AP, MeOH	[121]
**Acylphloroglucinol**	Hyperinoid A	NF-κB pathway; LPS-induced inflammatory response in macrophages	*In vitro*	Downregulation of mRNA levels of IL-1β, IL-6, iNOS; **Inhibition:** NF-κB pathway	IC_{50}=0.75±0.17 µmol/L vs IC_{50}=0.07±0.01 µmol/L Bortezomib control	AI	*Hypericum patulum* Thunb, AP, -	[122]

Compound Class	Compound	Measurement	Method	Outcome		Therapeutic Application	Species, Plant Part and Extract	Ref #
**Acylphloroglucinol**	Hyperinoid B	NF-κB pathway; LPS-induced inflammatory response in macrophages	*In vitro*	Downregulation of mRNA levels of IL-1β, IL-6, iNOS; **Inhibition:** NF-κB pathway	IC_{50}=1.19±0.48 µmol/L vs IC_{50}=0.07±0.01 µmol/L Bortezomib control	AI	*Hypericum patulum* Thunb, AP, -	[122]
**Acylphloroglucinol**	Wilsonxanthone A	Glucose transporter 4 activity	*In vitro* L6 cell model	Increased GLUT4 translocation	vs insulin positive control	ADb	*Hypericum wilsonii* N. Robson, AP, EtOH	**[123]**
**Acylphloroglucinol**	Furohyperforin	Glucose transporter 4 activity	*In vitro* L6 cell model	Increased GLUT4 translocation	vs insulin positive control	ADb	*Hypericum wilsonii* N. Robson, AP, EtOH	**[123]**
**Acylphloroglucinol**	Hyperwilone A	Glucose transporter 4 activity	*In vitro* L6 cell model	Increased GLUT4 translocation	vs insulin positive control	ADb	*Hypericum wilsonii* N. Robson, AP, EtOH	**[123]**
**Acylphloroglucinol**	Hyperwilone B	Glucose transporter 4 activity	*In vitro* L6 cell model	Increased GLUT4 translocation	vs insulin positive control	ADb	*Hypericum wilsonii* N. Robson, AP, EtOH	**[123]**

Compound Class	Compound	Measurement	Method	Outcome		Therapeutic Application	Species, Plant Part and Extract	Ref #
**Acylphloroglucinol**	Hyperwilone C	Glucose transporter 4 activity	*In vitro* L6 cell model	Increased GLUT4 translocation	vs insulin positive control	ADb	*Hypericum wilsonii* N. Robson, AP, EtOH	**[123]**
**Acylphloroglucinol**	Furoadhyperforin	Glucose transporter 4 activity	*In vitro* L6 cell model	Increased GLUT4 translocation	vs insulin positive control	ADb	*Hypericum wilsonii* N. Robson, AP, EtOH	**[123]**
**Acylphloroglucinol**	Pseudohenone	Glucose transporter 4 activity	*In vitro* L6 cell model	Increased GLUT4 translocation	vs insulin positive control	ADb	*Hypericum wilsonii* N. Robson, AP, EtOH	**[123]**
**Acylphloroglucinol**	Hyperscabin A	Cell viability, Serotonin reuptake	*In* vitro oxygen and glucose deprivation model<br>*In vitro* serotonin reuptake model	Increased cell viability<br>Decreased serotonin reuptake		CP, AD	*Hypericum scabrum* L., AP, EtOH	**[124]**
**Acylphloroglucinol**	Hyperscabin B	Serotonin reuptake	*In vitro* serotonin reuptake model	Decreased serotonin reuptake		AD	*Hypericum scabrum* L., AP, EtOH	**[124]**

Compound Class	Compound	Measurement	Method	Outcome	Therapeutic Application	Species, Plant Part and Extract	Ref #
**Acylphloroglucinol**	Norwilsonnol A	Cell proliferation	*Ex vivo* mice splenocytes	Decreased splenocyte proliferation, cytokine production	IM	*Hypericum wilsonii* N. Robson, S,L, EtOH	**[125]**
**Acylphloroglucinol**	Hypermonone E	Multidrug resistance	*In vitro* HepG2 and MCF-7 adriamycin resistant cell lines	Reversed drug resistance	AC	*Hypericum monogynum* L., Fl, MeOH	**[126]**
**Acylphloroglucinol**	Hypermonone F	Multidrug resistance	*In vitro* HepG2 and MCF-7 adriamycin resistant cell lines	Reversed drug resistance	AC	*Hypericum monogynum* L., Fl, MeOH	**[126]**
**Acylphloroglucinol**	Hypermonone I	Multidrug resistance	*In vitro* HepG2 and MCF-7 adriamycin resistant cell lines	Reversed drug resistance	AC	*Hypericum monogynum* L., Fl, MeOH	**[126]**
**Acylphloroglucinol**	Hyperforin	Expression modulation	*In vitro* murine splenic γδ T cells, and HaCaT cells; *In vivo* imiquimod induced mice model	Reduced epidermal thickness and decreased IMQ-induced pathological scores of cutaneous skin lesions in mice; TNFα levels Downregulated expression of inflammatory interleukins	AI	*Hypericum* spp., -, -	**[127]**

Compound Class	Compound	Measurement	Method	Outcome		Therapeutic Application	Species, Plant Part and Extract	Ref #
**Acylphloroglucinol**	Hyperpatulones E	α-glycosidase activity	*In vitro* model	Decreased α-glucosidase activity	IC_{50}= 37.69±2.05µM vs IC_{50}=156.18±6.12µM acarbose control	ADb	*Hypericum patulum* Thunb., L, EtOH	**[128]**
**Acylphloroglucinol**	Hyperpatulones F	α-glycosidase activity	*In vitro* model	Decreased α-glucosidase activity	IC_{50}= 20.99±4.49µM vs IC_{50}=156.18±6.12µM acarbose control	ADb	*Hypericum patulum* Thunb., L, EtOH	**[128]**
**Acylphloroglucinol**	Hyperpatulones G	α-glycosidase activity	*In vitro* model	Decreased α-glucosidase activity	IC_{50}= 14.06±4.44µM vs IC_{50}=156.18±6.12µM acarbose control	ADb	*Hypericum patulum* Thunb., L, EtOH	**[128]**
**Acylphloroglucinol**	Hypermonone A	Cell viability	*In vitro* SK-N-SH cells	Increased cell viability<br>Decreased induced cell toxicity		CP	*Hypericum beanii* N. Robson, AP, MeOH	**[129]**
**Acylphloroglucinol**	Uliginosin B	Antimicrobial activity	*In vitro Enterococcus faecalis, Staphylococcus aureus, MRSA*	Active against all tested strains (selective activity)		AM	*Hypericum* spp., -, -	**[130]**

Compound Class	Compound	Measurement	Method	Outcome		Therapeutic Application	Species, Plant Part and Extract	Ref #
**Acylphloroglucinol**	Japonicin A	Antimicrobial activity	*In vitro Enterococcus faecalis, Staphylococcus aureus, MRSA*	Active against all tested strains (selective activity)		AM	*Hypericum* spp., -, -	**[130]**
**Acylphloroglucinol**	Hyperbrasilol B	Antimicrobial activity	*In vitro Enterococcus faecalis, Staphylococcus aureus, MRSA*	Active against all tested strains (selective activity)		AM	*Hypericum* spp., -, -	**[130]**
**Acylphloroglucinol**	Spihyperglucinol A	Cell viability	*In vitro* LPS stimulated RAW 264.7 cells	Decreased NO production, cell damage Increased cell viability	IC_{50}= 8.7±1.18µM vs IC_{50}=9.76±1.13µM dexamethasone control	CP	*Hypericum longistylum* Oliv., AP, S, EtOH	**[131]**
**Acylphloroglucinol**	Spihyperglucinol B	Cell viability	*In vitro* LPS stimulated RAW 264.7 cells	Decreased NO production, cell damage Increased cell viability	IC_{50}= 9.23±1.26µM vs IC_{50}=9.76±1.13µM dexamethasone control	CP	*Hypericum longistylum* Oliv., AP, S, EtOH	**[131]**
**Acylphloroglucinol**	Hybeanone A	AChE activity	*In vitro* model	Decreased AChE activity		CP	*Hypericum beanii* N. Robson, AP, EtOH	**[132]**

Compound Class	Compound	Measurement	Method	Outcome		Therapeutic Application	Species, Plant Part and Extract	Ref #
**Acylphloroglucinol**	Hybeanone B	AChE activity	*In vitro* model	Decreased AChE activity		CP	*Hypericum beanii* N. Robson, AP, EtOH	**[132]**
**Acylphloroglucinol (analogue)**	Sarothralen C	Bacterial susceptibility	*In vitro Escherichia coli, Staphylococcus aureus, Salmonella typhimurium, Enterococcus faecalis*	Selective antimicrobial activity against bacterial species	MICs= 21.4μM (E.coli), 85.6μM (*S. aureus*), 5.4μM (*S. thyphimurium*), 10.7μM(*E. faecalis*) vs Cefotaxime control MICs= 0.4μM (*E. coli, E. faecalis*), 3.3μM (*S. aureus, S. typhimurium*)	AM	*Hypericum japonicum* Thunb., WP, MeOH	[61]
**Acylphloroglucinol (analogue)**	Sarothralin / Japonicin C	Bacterial susceptibility	*In vitro Escherichia coli, Staphylococcus aureus, Salmonella typhimurium, Enterococcus faecalis*	Selective antimicrobial activity against bacterial species	MICs= 1.9μM (*E.coli, S. aureus, E. faecalis*), 0.9μM (*S. thyphimurium*) vs Cefotaxime control MICs= 0.4μM (*E. coli, E. faecalis*). 3.3μM (*S. aureus, S. typhimurium*)	AM	*Hypericum japonicum* Thunb., WP, MeOH	[61]
**Acylphloroglucinol (analogue)**	Sarothralen A	Bacterial susceptibility	*In vitro Escherichia coli, Staphylococcus aureus, Salmonella typhimurium, Enterococcus faecalis*	Selective antimicrobial activity against bacterial species	MICs= 0.9μM (*E.coli*). 1.8μM (*S. aureus, E. faecalis, S. thyphimurium*) vs Cefotaxime control MICs= 0.4μM (*E. coli, E. faecalis*), 3.3μM (*S. aureus, S. typhimurium*)	AM	*Hypericum japonicum* Thunb., WP, MeOH	[61]

Compound Class	Compound	Measurement	Method	Outcome		Therapeutic Application	Species, Plant Part and Extract	Ref #
**Acylphloroglucinol derivative**	(+) Elodeoidol C	Oxidative stress	*In vitro* RAW264.7 cells	**Decreased** oxidative stress induced inflammatory damage	IC_{50}= 10.39±0.49 µM vs IC_{50}=2.90±0.98 N-monomethyl-L-arginine control	CP<br>AI	*Hypericum elodeoides* Choisy, AP, EtOH	[133]
**Acylphloroglucinol derivative**	(-) Elodeoidol C	Oxidative stress	*In vitro* RAW264.7 cells	**Decreased** oxidative stress induced inflammatory damage	IC_{50}= 21.41±2.41 µM vs IC_{50}=2.90±0.98 N-monomethyl-L-arginine control	CP<br>AI	*Hypericum elodeoides* Choisy, AP, EtOH	[133]
**Acylphloroglucinol derivative**	(+) Elodeoidol G	Oxidative stress	*In vitro* RAW264.7 cells	**Decreased** oxidative stress induced inflammatory damage	IC_{50}= 20.56±1.91 µM vs IC_{50}=2.90±0.98 N-monomethyl-L-arginine control	CP<br>AI	*Hypericum elodeoides* Choisy, AP, EtOH	[133]
**Acylphloroglucinol derivative**	(-) Elodeoidol G	Oxidative stress	*In vitro* RAW264.7 cells	**Decreased** oxidative stress induced inflammatory damage	IC_{50}= 34.25±2.32 µM vs IC_{50}=2.90±0.98 N-monomethyl-L-arginine control	CP<br>AI	*Hypericum elodeoides* Choisy, AP, EtOH	[133]
**Acylphloroglucinol derivative**	(+) Elodeoidol H	Oxidative stress, Bacterial susceptibility	*In vitro* RAW264.7 cells, *Fusobacterium nucleatum* subsp. *Polymorphun, Streptococcus mutans, Streptococcus sanguis*	**Decreased** oxidative stress induced inflammatory damage<br>**Decreased** bacterial growth	IC_{50}= 21.33±1.73 µM vs IC_{50}=2.90±0.98 N-monomethyl-L-arginine control<br>MIC= 25µg/mL vs MIC=3.91 - 0.98µg/mL cetylpyridinium chloride control	CP<br>AI<br>AM	*Hypericum elodeoides* Choisy, AP, EtOH	[133]

Compound Class	Compound	Measurement	Method	Outcome		Therapeutic Application	Species, Plant Part and Extract	Ref #
**Acylphloroglucinol derivative**	(-) Elodeoidol H	Oxidative stress, Bacterial susceptibility	*In vitro* RAW264.7 cells, *Fusobacterium nucleatum* subsp. *Polymorphun, Streptococcus mutans*, *Streptococcus sanguis*	**Decreased** oxidative stress induced inflammatory damage **Decreased** bacterial growth	IC_{50}= 28.96±1.19 μM vs IC_{50}=2.90±0.98 N-monomethyl-L-arginine control MIC= 25μg/mL vs MIC=3.91 - 0.98μg/mL cetylpyridinium chloride control	CP AI AM	*Hypericum elodeoides* Choisy, AP, EtOH	[133]
**Acylphloroglucinol derivative**	(+) Elodeoidol E	Bacterial susceptibility	*In vitro* *Fusobacterium nucleatum* subsp. *Polymorphun, Streptococcus mutans*, *Streptococcus sanguis*	**Decreased** bacterial growth	MIC= 25 - 6.25μg/mL vs MIC=3.91 - 0.98μg/mL cetylpyridinium chloride control	AM	*Hypericum elodeoides* Choisy, AP, EtOH	[133]
**Acylphloroglucinol derivative**	(-) Elodeoidol E	Bacterial susceptibility	*In vitro* *Fusobacterium nucleatum* subsp. *Polymorphun, Streptococcus mutans*, *Streptococcus sanguis*	**Decreased** bacterial growth	MIC= >25μg/mL vs MIC=3.91 - 0.98μg/mL cetylpyridinium chloride control	AM	*Hypericum elodeoides* Choisy, AP, EtOH	[133]

**Compound Class**	**Compound**	**Measurement**	**Method**	**Outcome**		**Therapeutic Application**	**Species, Plant Part and Extract**	**Ref #**
**Acylphloroglucinol derivative**	(+) Elodeoidol I	Bacterial susceptibility	*In vitro* *Fusobacterium nucleatum* subsp. *Polymorphun, Streptococcus mutans, Streptococcus sanguis*	**Decreased** bacterial growth	MIC= >25μg/mL vs MIC=3.91 - 0.98μg/mL cetylpyridinium chloride control	AM	*Hypericum elodeoides* Choisy, AP, EtOH	[133]
**Acylphloroglucinol derivative**	(-) Elodeoidol I	Bacterial susceptibility	*In vitro* *Fusobacterium nucleatum* subsp. *Polymorphun, Streptococcus mutans, Streptococcus sanguis*	**Decreased** bacterial growth	MIC= >25μg/mL vs MIC=3.91 - 0.98μg/mL cetylpyridinium chloride control	AM	*Hypericum elodeoides* Choisy, AP, EtOH	[133]
**Anthraquinone**	Hypericin	Cell viability, Oxidative stress	*In vitro*	**Increased** cell viability **Decreased** NO LPS induced production	**Decreased** COX2, iNOS, TNFα, IL-1 β, IL-6 gene expression level	CP	*Hypericum hookerianum* Wight & Arn., -, -	[134]
**Anthraquinone**	Hypericin	Bacterial susceptibility	*In vitro* *S. aureus*	**Increased** antimicrobial activity against *S. aureus* when combined with carvacrol	Hypericin MIC **decreased** when combined with carvacrol Hypericin disk diffusion radius **increased** when combined with carvacrol	AM	*Hypericum perforatum* L., WP, EtOH	[135]

Compound Class	Compound	Measurement	Method	Outcome		Therapeutic Application	Species, Plant Part and Extract	Ref #
**Anthraquinone**	Emodin	Cell viability, Oxidative stress	*In vitro* A375, MDA-MB-231, SiHa, SHSY-5Y cancer cell lines; LPS stimulated RAW264,7, THP-1 and BV-2 inflammatory model	**Decreased** cell viability (selective cytotoxicity) **Decreased** oxidative stress induced inflammatory damage	IC_{50}= 84.41±0.03 - 8.56±0.32 µM vs IC_{50}=15.74±0.13 - 5.14±0.16 µM Cisplatin control IC_{50}= 17.06±0.80 - 12.39±1.0 µM vs IC_{50}=19.37±0.72 - 15.20±1.10 µM Indomethacin control	AC AI	*Hypericum sampsonii* Hance, WP, EtOH	[136]
**Anthraquinone**	3-ethyl-1,8-dihydroxy-6-methoxyanthracene-9,10-dione	Cell viability, Oxidative stress	*In vitro* A375, MDA-MB-231, SiHa, SHSY-5Y cancer cell lines; LPS stimulated RAW264,7, THP-1 and BV-2 inflammatory model	**Decreased** cell viability (selective cytotoxicity) **Decreased** oxidative stress induced inflammatory damage	IC_{50}= 106.30±0.27 - 32.21±1.77 µM vs IC_{50}=15.74±0.13 - 5.14±0.16 µM Cisplatin control IC_{50}= 17.9±0.51 - 14.11±0.53 µM vs IC_{50}=19.37±0.72 - 15.20±1.10 µM Indomethacin control	AC AI	*Hypericum sampsonii* Hance, WP, EtOH	[136]
**Anthraquinone**	Hypericin	Cell viability	*In vivo* Thyroid cancer mouse model FRO cells	**Decreased** tumour growth	**Increased** intracellular ROS **Increased** cell death in combination with power dependent laser application	PDT	*Hypericum* spp., -, -	[137]

Compound Class	Compound	Measurement	Method	Outcome		Therapeutic Application	Species, Plant Part and Extract	Ref #
**Anthraquinone**	Hypericin	Cell viability	*In vitro*<br>HT-29 cells	Increased intracellular hypericin concentration might be correlated with anti-carcinogenic effect of HYP-mediated PDT		PDT	*Hypericum* spp., -	[138]
**Anthraquinone**	Hypericin	Cell Viability	*In vitro*<br>HUVEC cells	**Decreased** apoptosis	**Increased** Bcl-2 expression, cell viability<br>**Decreased** Bax, p53 expression, MAPKs activation, AGEs formation, ROS generation	CP	*Hypericum* spp., -, -	[139]
**Anthraquinone**	Hypericin	Cell viability CYP1A2 and CYP2D6 expression	*In vitro*<br>WRL-68, HepG2	**Increased** CYP1A2, CYP2D6 (HepG2) expression<br>**Decreased** CYP1A2 (HepaRG), CYP2D6 (HepaRG, WRL-68) expression		Int	*Hypericum* spp., -, -	[102]
**Anthraquinone**	Hypericin	Cell viability	*In vitro*<br>MCF-7 cells	**Decreased** cell viability	LD_{50}= 5 μg/mL vs 20 μg/mL Cysplatin control 24h<br>LD_{50}= 0.5 μg/mL vs 7.5 μg/mL Cysplatin control 48h<br>**Decreased** MDF-7 cell growth rate, Bcl2 expression<br>**Increased** p3 expression, apoptosis	AC	*Hypericum* spp., -, -	[20]

Compound Class	Compound	Measurement	Method	Outcome		Therapeutic Application	Species, Plant Part and Extract	Ref #
**Anthraquinone**	Hypericin	Stress-induced depressive behaviours	*In vivo* mouse model	**Increased** body weight, independent behavior, pleasant stimulus response, **Decreased** stress hormone levels		AD	*Hypericum* spp., -, -	[16]
**Anthraquinone**	Hypericin	Cell viability, apoptosis	*In vitro* RINm5F cells	**Increased** apoptosis **Decreased** cell viability (photoactivated); cell proliferation (photoactivated)	IC_{50}= 105.97 nM **Increased** cleaved casp-3, cleaved casp-9, Bac-t-Bcl-2 ratio **Decreased** phospho-JNK and phospho-ERK	AC	*Hypericum* spp., -, -	[21]
**Anthraquinone**	Hypericin	*Leishmania (Viannia) panamensis* infected macrophages Wound healing	*In vitro* U-937 promonocytes, Detroit 551 fibroblast cells *In vivo* mouse model	**Increased** wound healing (*in vitro* and *in vivo*) **Decreased** (Photoactivation dependent) intracellular parasite load	Antileishmanial activity: IC_{50}=2.5 ± 0.14 µM (no photoactivation). IC_{50}= 1.2 ± 0.007 µM (photoactivated) vs IC_{50}=0.61±0.005 µM AmB control **Increased** wound healing growth factors production (EGF, PDGF, FGF)	S, AP	*Hypericum* spp., -, -	[140]

Compound Class	Compound	Measurement	Method	Outcome		Therapeutic Application	Species, Plant Part and Extract	Ref #
**Anthraquinone**	Hypericin	Cell viability	*In vitro* A2780, A2780cis, HL-60, cBCRP cells	**Decreased** cisplatin and mitoxantrone induced metabolic inhibition (A2780, A2780cis, HL-60); cisplatin induced A2780/A2780cis cell death; mitoxantrone induced HL-60 cell death; **Increased** mitoxantrone induced cBCRP cell death	**Increased** MRP1 expression (A2780/A2780cis)	CP	*Hypericum* spp., -, -	[141]
**Anthraquinone**	Hypericin	Photodynamic therapy	*In vitro*	Potent photosensitizer after topical application and excitation by laser light of wavelength 405 nm		PDT	*Hypericum* spp., -, -	[142]
**Anthraquinone**	Hypericin	Induced nociceptive behaviour	*In vivo* mouse model	**Decreased** NO induced painful behaviour	**Decreased** NO induced p-CREB expression, p-STAT1 upregulation, NF-κB activation, PKC expression	AD, AN	*Hypericum* spp., -, -	[143]
**Anthraquinone**	Hypericin	Cell viability	*In vitro* SCC cells	**Decreased** cell viability	**Increased** ROS formation	AC	*Hypericum* spp., -, -	[23]

Compound Class	Compound	Measurement	Method	Outcome		Therapeutic Application	Species, Plant Part and Extract	Ref #
**Anthraquinone**	Hypericin	GST-pi activity	*In vitro*	**Decreased** GST-pi activity		CP	*Hypericum* spp., -, -	[144]
**Anthraquinone**	Hypericin	Action potential and voltage-gated Na^+, I_A and I_K currents	*In vitro*	**Increased** Action potential duration **Inhibited** transient I_A and delayed I_K K currents		AD	*Hypericum* spp., -, -	[145]
**Anthraquinone**	Hypericin	Glutamate release	*In vitro*	**Decreased** glutamate release	**Decreased** vesicular exocytosis, presynaptic N- and P/Q-type voltage-dependent Ca^{2+} channel activity, MAPK activity	AD	*Hypericum* spp., -, -	[146]
**Anthraquinone**	Hypericin	Cell viability	*In vitro* A431 cells	**Decreased** cell viability (light dependent) **Increased** apoptosis		AC	*Hypericum* spp., -, -	[25]

Compound Class	Compound	Measurement	Method	Outcome		Therapeutic Application	Species, Plant Part and Extract	Ref #
**Anthraquinone**	Hypericin	Cell division, DNA damage	*In vitro* hamster lung fibroblast model (V79 cells)	**Decreased** doxorubicin and methanesulfonate induced DNA damage	Combination of hypericin+methanesulfonate decreased DNA damage up to 59.99% (concentration dependent) Combination of hypericin+doxorubicin decreased DNA damage up to 60.38% (concentration dependent)	CP AC	*Hypericum* spp., -, -	[147]
**Anthraquinone**	Hypericin	Pancreatic lipase activity	*In vitro* pancreatic lipase model	**Decreased** pancreatic lipase activity	IC_{50}= 0.97±0.09 µM vs IC_{50}=2.90±0.98 Isoginkgetin control vs IC_{50}=0.75 nM Orlistat control	Ad.In	*Hypericum* spp., -, -	[148]
**Anthraquinone**	Hypericin	SARS-CoV-2 activity	*In vitro* Vero-E6 infected cells	**Decreased** SARS-CoV-2 replication		AV	*Hypericum* spp., -, -	**[149]**
**Anthraquinone**	Hypericin	Depressive behaviours	*In* vivo mouse depression model *In vitro* C2C12 cells	Decreased fatigue, oxidative stress Increased swimming time in forced swimming test	Decreased expression o TNF-α, IL-1β, IL-6 and INF-γ	AD	*Hypericum* spp., -, -	**[150]**
**Anthraquinone**	Hypericin	*In vivo* mouse depression model			Decreased IL-6, IL-1β, TNF-α expression	AD	*Hypericum* spp., -, -	**[151]**

**Compound Class**	**Compound**	**Measurement**	**Method**	**Outcome**		**Therapeutic Application**	**Species, Plant Part and Extract**	**Ref #**
**Anthraquinone**	Pseudohypericin	Pancreatic lipase activity	*In vitro* pancreatic lipase model	**Decreased** pancreatic lipase activity	IC_{50}= 0.94±0.11 µM vs IC_{50}=2.90±0.98 Isoginkgetin control vs IC_{50}=0.75 nM Orlistat control	Ad.In	*Hypericum* spp., -, -	[148]
**Anthraquinone**	Protohypericin	Pancreatic lipase activity	*In vitro* pancreatic lipase model	**Decreased** pancreatic lipase activity	IC_{50}= 2.80±0.31 µM vs IC_{50}=2.90±0.98 Isoginkgetin control vs IC_{50}=0.75 nM Orlistat control	Ad.In	*Hypericum* spp., -, -	[148]
**Anthraquinone**	Emodin	Pancreatic lipase activity	*In vitro* pancreatic lipase model	**Decreased** pancreatic lipase activity	IC_{50}= 2.18±0.09 µM vs IC_{50}=2.90±0.98 Isoginkgetin control vs IC_{50}=0.75 nM Orlistat control	Ad.In	*Hypericum* spp., -, -	[148]
**Benzophenone**	Sampsonione O	Cell viability	*In vitro* SK-N-SH cells	**Increased** cell viability	Survival rate: 80.90% vs 62.10 donepezil control vs 63.20 % PHPB control	CP	*Hypericum acmosepalum* N.Robson, AP, EtOH	[33]
**Benzophenone**	Cariphenone A	Cell viability	*In vitro* U-251, HT-29, OVCAR-3 cells	**Decreased** cell viability (selective toxicity)	Cell viability% (HT-29): 47.0±1.8% vs 30.0±4.3% Irinotecan control<br>Cell viability% (OVCAR-3): 68.0±1.4% vs 58.0±7.9% Paclitaxel control<br>Cell viability% (U-251): 46.0±2.0% vs 40.0±8.0% Termozolomide control	AC	*Hypericum carinatum* Griseb., AP, n-Hexane	[70]

Compound Class	Compound	Measurement	Method	Outcome		Therapeutic Application	Species, Plant Part and Extract	Ref #
**Benzophenone**	Cariphenone B	Cell viability	*In vitro* U-251, HT-29, OVCAR-3 cells	**Decreased** cell viability (selective toxicity)	Cell viability% (HT-29): 63.0±3.1% vs 30.0±4.3% Irinotecan control Cell viability% (OVCAR-3): 73.0±3.1% vs 58.0±7.9% Paclitaxel control Cell viability% (U-251): 47.0±1.8% vs 40.0±8.0% Termozolomide control	AC	*Hypericum carinatum* Griseb., AP, n-Hexane	[70]
**Benzophenone**	Elegaphenone	Cell viability	*In vitro* HD-MY-Z, K-562, KE-37 cells	**Decreased** cell viability (selective activity) **Increased** apoptosis	IC_{50} = 16.9±1.6μM - 13.9±1.2μM vs IC_{50} = 2.1±0.11μM - 0.6±0.01μM Daunorubicine control	AC	*Hypericum elegans* Stephan ex Willd., AP, DCM	[152]
**Benzophenone**	7-epiclusianone	Cell viability	*In vitro* HD-MY-Z, K-562, KE-37 cells	**Decreased** cell viability (selective activity) **Increased** apoptosis	IC_{50} = 13.6±1.5μM - 9.8±1.4μM vs IC_{50} = 2.1±0.11μM - 0.6±0.01μM Daunorubicine control	AC	*Hypericum elegans* Stephan ex Willd., AP, DCM	[152]
**Benzophenone**	2,2',5,6'-Tetrahydroxybenzophenone	Protozoal susceptibility	*In vitro Plasmodium falciparum*	Active against all tested strains	IC_{50}=55.12±0.93μg/mL - 13.41±0.16μg/mL vs IC_{50}=0.27±0.04μg/mL - 0.14±0.05μg/mL quinine control	Mal	*Hypericum lanceolatum* Lam., Sb, MeOH	[153]
**Benzophenone**	Hyperewalone B	Cell viability, oxidative stress	*In vitro* BV-2 cell lines	**Decreased** LPS induced NO production	IC_{50}= 0.61±0.12 μM vs IC_{50}=4.00±0.23μM Quercetin control	CP	*Hypericum przewalskii* Maxim., AP, MeOH	[154]

Compound Class	Compound	Measurement	Method	Outcome		Therapeutic Application	Species, Plant Part and Extract	Ref #
**Benzophenone**	Hyperprzeone A	Cell viability	*In vitro* SH-SY5Y, MDA-MB-231, SiHa, SMMC-7721, NCI-H460 and A375 cell lines	**Decreased** cell viability (selective cytotoxicity)	IC_{50}= 124.41±4.16 - 25.54±0.63µM vs IC_{50}=12.49±1.12 - 0.34±0.02µM Dexamethasone control	AC	*Hypericum przewalskii* Maxim., WP, EtOH	[155]
**Benzophenone**	Sampsonin A	RXRα transcription Cell viability	*In vitro* HeLa, 293T cells	**Decreased** cell viability, RXRα transcription		AC	*Hypericum sampsonii* Hance, AP, EtOH	[156]
**Benzophenone**	Sampsonin B	RXRα transcription Cell viability	*In vitro* HeLa, 293T cells	**Decreased** cell viability, RXRα transcription		AC	*Hypericum sampsonii* Hance, AP, EtOH	[156]
**Benzophenone**	Garcimangosone D	Cell viability, Oxidative stress	*In vitro* A375, MDA-MB-231, SiHa, SHSY-5Y cancer cell lines; LPS stimulated RAW264,7, THP-1 and BV-2 inflammatory model	**Decreased** cell viability (selective cytotoxicity) **Decreased** oxidative stress induced inflammatory damage	IC_{50}= 107.73±0.25 - 24.67±0.11 µM vs IC_{50}=15.74±0.13 - 5.14±0.16 µM Cisplatin control IC_{50}= 19.14±0.37 - 14.52±0.64 µM vs IC_{50}=19.37±0.72 - 15.20±1.10 µM Indomethacin control	AC AI	*Hypericum sampsonii* Hance, WP, EtOH	[136]
**Benzophenone**	*Hypericum*one A	Cell viability, Oxidative stress	*In vitro* RAW264.7 cells	**Decreased** oxidative stress induced inflammatory damage	**Decreased** NO production $IC_{50} \leq 40.32$ µM	CP AI	*Hypericum sampsonii* Hance, AP, MeOH	[157]

Compound Class	Compound	Measurement	Method	Outcome		Therapeutic Application	Species, Plant Part and Extract	Ref #
**Benzophenone**	Sampsine A	Oxidative stress	*In vitro* RAW264.7 cell lines	**Decreased** LPS-induced NO production	IC_{50}= 2.4±0.69µM *vs* IC_{50}=1.41±0.03µM cadamonin control	CP	*Hypericum sampsonii* Hance, AP, MeOH	[158]
**Benzophenone**	Sampsine B	Oxidative stress	*In vitro* RAW264.7 cell lines	**Decreased** LPS-induced NO production	IC_{50}= 2.29±0.12µM *vs* IC_{50}=1.41±0.03µM cadamonin control	CP	*Hypericum sampsonii* Hance, AP, MeOH	[158]
**Benzophenone**	Petiolin F	Oxidative stress	*In vitro* RAW264.7 cell lines	**Decreased** LPS-induced NO production	IC_{50}= 2.0±0.34µM *vs* IC_{50}=1.41±0.03µM cadamonin control	CP	*Hypericum sampsonii* Hance, AP, MeOH	[158]
**Benzophenone**	Cariphenone B	Cell proliferation	*In vitro* HaCaT, MRC5 and MSC	**Increased** HaCaT proliferation	0.1 µM (114% vs blank control)<br>0.01 µM (122.3% vs blank control)	S	*Hypericum* spp., AP, n-Hexane	[97]
**Benzopyrane**	6-isobutyryl-5,7-dimethoxy-2,2-dimethylbenzopyran	Protozoal susceptibility	*In vitro Trichomonas vaginalis*	**Decreased** *T. vaginalis* cell viability (including metronidazole resistant strain)	IC_{50}=226.50µM	AP	*Hypericum polyanthemum* Klotzsch ex Reichardt, AP, Supercritical CO2	[85]
**Benzopyrane**	7-hydroxy-6-isobutyryl-5-methoxy-2,2-dimethyl-benzopyran	Protozoal susceptibility	*In vitro Trichomonas vaginalis*	**Decreased** *T. vaginalis* cell viability	IC_{50}=833.65µM	AP	*Hypericum polyanthemum* Klotzsch ex Reichardt, AP, Supercritical CO2	[85]

Compound Class	Compound	Measurement	Method	Outcome		Therapeutic Application	Species, Plant Part and Extract	Ref #
**Benzopyrane**	6-isobutyryl-5,7-dimethoxy-2,2-dimethylbenzopyran	Stress-induced depressive behaviours	*In vivo* mouse model	**Decreased** painful behaviours		AD	*Hypericum polyanthemum* Klotzsch ex Reichardt, AP, n-Hexane	[159]
**Benzopyrane**	7-hydroxy-6-isobutyryl-5-methoxy-2,2-dimethyl-benzopyran	Cell proliferation	*In vitro* HaCaT, MRC5 and MSC	**Increased** HaCaT proliferation	5 µM (114.3% vs blank control)	S	*Hypericum* spp., AP, n-Hexane	[97]
**Benzoylphloroglucinol**	Hyperscabrone K	Cell viability	*In vitro* HepG2 cells	**Increased** cell viability	Cell viability 54.5±1.4 % vs 54.8±2.0% Bicyclol control	CP	*Hypericum scabrum* L., AP, EtOH	[160]
**Benzoylphloroglucinol**	Hyperscabrone M	Cell viability	*In vitro* HepG2 cells	**Increased** cell viability	Cell viability 60.2±3.0 % vs 54.8±2.0% Bicyclol control	CP	*Hypericum scabrum* L., AP, EtOH	[160]
**BiFlavone**	Biapigenin	Radical scavenging	*In vitro* $HepG_2$ cells	Coadministration of compounds included in formulated nanoparticles showed free radicals scavenging activity		CP	*Hypericum perforatum* L., AP, MeOH	[161]
**BiFlavone**	Amentoflavone	Pancreatic lipase activity	*In vitro* pancreatic lipase model	**Decreased** pancreatic lipase activity	IC_{50}= 9.94±0.41 µM vs IC_{50}=2.90±0.98 Isoginkgetin control vs IC_{50}=0.75 nM Orlistat control	Ad.In	*Hypericum* spp., -, -	[148]

Compound Class	Compound	Measurement	Method	Outcome		Therapeutic Application	Species, Plant Part and Extract	Ref #
**BiFlavone**	I3,II8-Biapigenin	Pancreatic lipase activity	*In vitro*<br>pancreatic lipase model	**Decreased** pancreatic lipase activity	IC_{50}= 0.78±0.03 µM vs IC_{50}=2.90±0.98 Isoginkgetin control vs IC_{50}=0.75 nM Orlistat control	Ad.In	*Hypericum* spp., -, -	[148]
**Chromanone**	Aucherine A	Cell viability	*In vitro*<br>MDA-MB, EJ, SKW-3, HL-60, HL-60/DOX cell	**Decreased** cell viability	**Decreased:** Procaspase-9 activation, Bcl-xl expression<br>Moderate cytotoxicity vs etoposide control	AC	*Hypericum aucheri* Jaub. & Spach, AP, DCM (MeOH f)	[162]
**Chromanone**	Aucherine B	Cell viability	*In vitro*<br>MDA-MB, EJ, SKW-3, HL-60, HL-60/DOX cell	**Decreased** cell viability	**Decreased:** Procaspase-9 activation, Bcl-xl expression<br>Moderate cytotoxicity vs etoposide control	AC	*Hypericum aucheri* Jaub. & Spach, AP, DCM (MeOH f)	[162]
**Chromanone**	Aucherine C	Cell viability	*In vitro*<br>MDA-MB, EJ, SKW-3, HL-60, HL-60/DOX cell	**Decreased** cell viability	**Decreased:** Procaspase-9 activation. Bcl-xl expression<br>Moderate cytotoxicity vs etoposide control	AC	*Hypericum aucheri* Jaub. & Spach, AP, DCM (MeOH f)	[162]
**Chromone**	(S)-(+)-5,7-dihydroxy-2-(1-methylpropyl) chromone	RXRα transcription<br>Cell viability	*In vitro*<br>293T, HeLa cells	**Induced** apoptosis<br>**Inhibited** HeLa cells proliferation (selectively), RXRα transcription	**Increased**: caspase-8 activation, PARP cleavage	AC	*Hypericum elodeoides* Choisy, WP, EtOH	[163]
**Flavanol**	(-) epicatechin	Cell viability, Oxidative stress	*In vitro*<br>PC12, BV-2 cells	**Increased** cell viability<br>**Decreased** NO LPS induced production	Cell viability: 47.61 - 83.26% vs 64.92 - 89.34 % captopril control<br>IC_{50}= 3.37 ± 0.13 µM vs IC_{50}= 1.07 ± 0.04 µM Quercetin control vs IC_{50}= 2.6 ± 0.06 µM Fluoxetine control	AI	*Hypericum elatoides* R.Keller, AP, MeOH	[164]

Compound Class	Compound	Measurement	Method	Outcome		Therapeutic Application	Species, Plant Part and Extract	Ref #
**Flavanone**	Calycinigin A	Cell viability, Oxidative stress	*In vitro* HeLa cells	**Decreased** cell viability; ROS	Cell viability IC_{50}=9.7±1.8μM vs IC_{50}=0.05±0.01μM Podophyllotoxin control Antioxidant activity (Trolox equivalents)= 2.3±0.2	AC CP	*Hypericum calycinum* L., S, EtOH	[165]
**Flavanone**	Exiguaflavanone J	Cell viability, Oxidative stress	*In vitro* HeLa cells	**Decreased** cell viability; ROS	Cell viability IC_{50}=11.6±0.9μM vs IC_{50}=0.05±0.01μM Podophyllotoxin control Antioxidant activity (Trolox equivalents)= 0.6±0.1	AC CP	*Hypericum calycinum* L., S, EtOH	[165]
**Flavanone**	Exiguaflavanone I	Cell viability, Oxidative stress	*In vitro* HeLa cells	**Decreased** cell viability; ROS	Cell viability IC_{50}=19.3±1.5μM vs IC_{50}=0.05±0.01μM Podophyllotoxin control Antioxidant activity (Trolox equivalents)= 0.6±0.1	AC CP	*Hypericum calycinum* L., S, EtOH	[165]
**Flavanone**	Exiguaflavanone C	Cell viability, Oxidative stress	*In vitro* HeLa cells	**Decreased** cell viability; ROS	Cell viability IC_{50}=40.7±2.4μM vs IC_{50}=0.05±0.01μM Podophyllotoxin control Antioxidant activity (Trolox equivalents)= 1.6±0.2	AC CP	*Hypericum calycinum* L., S, EtOH	[165]
**Flavanone**	Hyperelatone B	Cell viability, Oxidative stress	*In vitro* PC12, BV-2 cells	**Increased** cell viability	Cell viability: 64.38 - 91.98% vs 64.92 - 89.34 % captopril control	AI	*Hypericum elatoides* R.Keller, AP, MeOH	[164]

Compound Class	Compound	Measurement	Method	Outcome		Therapeutic Application	Species, Plant Part and Extract	Ref #
**Flavanone**	Hyperelatone C	Cell viability, Oxidative stress	*In vitro* PC12, BV-2 cells	**Increased** cell viability	Cell viability: 51.51 - 90.96% vs 64.92 - 89.34 % captopril control	AI	*Hypericum elatoides* R.Keller, AP, MeOH	[164]
**Flavanone**	Hyperelatone D	Cell viability, Oxidative stress	*In vitro* PC12, BV-2 cells	**Increased** cell viability **Decreased** NO LPS induced production	Cell viability: 48.43 - 84.74% vs 64.92 - 89.34 % captopril control IC_{50}= 5.83 ± 0.23 µM vs IC_{50}= 1.07 ± 0.04 µM Quercetin control vs IC_{50}= 2.6 ± 0.06 µM Fluoxetine control	AI	*Hypericum elatoides* R.Keller, AP, MeOH	[164]
**Flavanone**	5,7,3′,5′-tetrahydroxyflavanone-7-O-glucoside	HIV-1 reverse transcriptase associated functions	*In vitro* Vero-76, A549, HepG2, HeLa, TZM-bl, T-lymphoid Jurkat cells	**Decreased** viral RNAse H and RDDP activity **Decreased** HIV-1 infected cells viability, HIV-1 replication	RNAse H IC_{50}=33±3µM vs IC_{50}=8.1±2.2µM RDS1643 control vs IC_{50}=12±3µM K-49 control RDDP IC_{50}=80±3µM vs IC_{50}=0.013±0.004µM Efavirenz control vs IC_{50}=11±2µM K-49 control	AV	*Hypericum hircinum* L., AP, EtOH	[166]
**Flavanone**	(S)-4',5-dihydroxy-7-methoxyflavanone	Lipoxygenase activity	*In vitro*	**Decreased** Lypoxygenase activity	IC_{50}= 31.8 ± 0.10 µM vs IC_{50}= 22.0 ± 0.04 µM Baicalein control	AI	*Hypericum oblongifolium* Choisy, WP, MeOH (Chl f)	[167]
**Flavanone**	5,4′-dihydroxy-3′-methoxy-(6:7)-2,2-dimethylpyranoflavone	Cell viability	*In vitro* DLD-1 cells	**Decreased** cell viability	IC_{50}= 6.28µM	AC	*Hypericum nokoense* Ohwi, AP, MeOH	**[168]**

Compound Class	Compound	Measurement	Method	Outcome		Therapeutic Application	Species, Plant Part and Extract	Ref #
**Flavanonol**	taxifolin-7-O-α-L-rhamnopyranoside	Bacterial susceptibility	*In vitro MRSA*	Active against all tested strains (selective activity)	MIC = 64-8µg/mL vs MIC = 128-16µg/mL Ampicillin control vs MIC = 512-32µg/mL Ceftazidime control vs MIC=0.96µg/mL Vancomycin control	AM	*Hypericum japonicum* Thunb., AP, -	[169]
**Flavonoid**	Quercetin	Antimicrobial activity	*In vitro* antitrypanosomal model	**Decreased** trypanosomal viability	IC_{50}= 7.52µM IC_{90}= 9.76µM	AP	*Hypericum afrum* Lam., AP, EtOH	[170]
**Flavonoid**	Myricetin	Antimicrobial activity	*In vitro* antitrypanosomal model	**Decreased** trypanosomal viability	IC_{50}= 5.71µM IC_{90}= 7.97µM	AP	*Hypericum afrum* Lam., AP, EtOH	[170]
**Flavonoid**	Astragalin	α-glucosidase activity	*In vitro* model	**Decreased** α-glucosidase activity		ADb	*Hypericum attenuatum* Fisch. ex Choisy, AP, EtOH	[171]
**Flavonoid**	Guaijaverin	α-glucosidase activity	*In vitro* model	**Decreased** α-glucosidase activity		ADb	*Hypericum attenuatum* Fisch. ex Choisy, AP, EtOH	[171]
**Flavonoid**	Quercetin	α-glucosidase activity	*In vitro* model	**Decreased** α-glucosidase activity		ADb	*Hypericum attenuatum* Fisch. ex Choisy, AP, EtOH	[171]

Compound Class	Compound	Measurement	Method	Outcome		Therapeutic Application	Species, Plant Part and Extract	Ref #
**Flavonoid**	Quercetin	Cell viability	*In vivo* hepatotoxicity rat model	**Decreased** induced liver injury, oxidative stress	**Decreased** MDA, ALT, AST, DBIL, TBIL, TBA and γ-GGT levels; PTGS2, CYP7A1, IL-1β, TNF-α expression **Increased** SOD and GSH-Px levels**;** BCL2and FXR expression	CP	*Hypericum japonicum* Thunb., -, -	[172]
**Flavonoid**	Folecitin	Oxidative stress	*In vivo* rat model	**Decreased** EtOH induced oxidative stress, p-JNK expression, NLRP3-inflammasome complexation **Decreased** caspase-3, BAX, BCL-2 and PARP-1 expression	**Decreased** neuroinflammatory and neurodegenerative protein markers	CP	*Hypericum oblongifolium* Choisy, L, MeOH	[173]
**Flavonoid**	Quercetin-3-O-α-L-rhamnoside	Glucose consumption	*In vitro* insulin-resistant HepG2 cell model	**Increased** glucose consumption in hyperglycemic induced HepG2 cells **Increased** PPARγ expression		ADb	*Hypericum patulum* Thunb., WP, EtOH	[174]
**Flavonoid**	Quercetin-3-O-(4-”methoxy)-α-L-rahmnopyranosyl	Glucose consumption	*In vitro* insulin-resistant HepG2 cell model	**Increased** glucose consumption in hyperglycemic induced HepG2 cells **Increased** PPARγ expression		ADb	*Hypericum patulum* Thunb., WP, EtOH	[174]

Compound Class	Compound	Measurement	Method	Outcome		Therapeutic Application	Species, Plant Part and Extract	Ref #
**Flavonoid**	Hyperoside (Quercetin-3-O-β-D-galactopyranoside)	Glucose consumption	*In vitro* insulin-resistant HepG2 cell model	**Increased** glucose consumption in hyperglycemic induced HepG2 cells<br>**Increased** PPARγ expression		ADb	*Hypericum patulum* Thunb., WP, EtOH	[174]
**Flavonoid**	Astilbin	Cell viability, Oxidative stress	*In vitro/In vivo* osteoarthritis model	**Increased** cell viability	**Decreased** NO, PGE2, TNFα, IL-6 production<br>**Decreased** iNOS, COX-2, MMP13, ADAMTS5 expression<br>**Increased** *in vivo* chondrocyte protection	AI CP	*Hypericum perforatum* L., -, -	[175]
**Flavonoid**	Hyperoside (Quercetin-3-O-β-D-galactopyranoside)	Oxidative stress	*In vitro/In vivo* induced liver injury model	**Decreased** oxidative stress and CCl4 induced liver damage	**Decreased** MDA levels, PHLPP2 expression<br>**Increased** SOD, Nrf2 translocation, HO-1 expression	AI, IM	*Hypericum perforatum* L., -, -	[176]
**Flavonoid**	Myricetin 3-O-a-L-rhamnopyranoside	Cell viability, oxidative stress	*In vitro* BV-2 cell lines	**Decreased** LPS induced NO production	IC_{50}= 4.90±0.60 μM vs IC_{50}=4.00±0.23μM Quercetin control	CP	*Hypericum przewalskii* Maxim., AP, MeOH	[154]
**Flavonoid**	Quercetin 3-O-a-L-rhamnopyranoside	Cell viability, oxidative stress	*In vitro* BV-2 cell lines	**Decreased** LPS induced NO production	IC_{50}= 1.05±0.03 μM vs IC_{50}=4.00±0.23μM Quercetin control	CP	*Hypericum przewalskii* Maxim., AP, MeOH	[154]
**Flavonoid**	Quercetin 3-O-glucopyranoside	Cell viability, oxidative stress	*In vitro* BV-2 cell lines	**Decreased** LPS induced NO production	IC_{50}= 1.26±0.04 μM vs IC_{50}=4.00±0.23μM Quercetin control	CP	*Hypericum przewalskii* Maxim., AP, MeOH	[154]

Compound Class	Compound	Measurement	Method	Outcome		Therapeutic Application	Species, Plant Part and Extract	Ref #
**Flavonoid**	Quercetin	Cell viability, Oxidative stress	*In vitro* A375, MDA-MB-231, SiHa, SHSY-5Y cancer cell lines; LPS stimulated RAW264,7, THP-1 and BV-2 inflammatory model	**Decreased** oxidative stress induced inflammatory damage	IC_{50}= 15.92±0.63 - 10.59±0.55 µM vs IC_{50}=19.37±0.72 - 15.20±1.10 µM Indomethacin control	AI	*Hypericum sampsonii* Hance, WP, EtOH	[136]
**Flavonoid**	Hyperoside (Quercetin-3-O-β-D-galactopyranoside)	Cell viability, Oxidative stress	*In vitro* A375, MDA-MB-231, SiHa, SHSY-5Y cancer cell lines; LPS stimulated RAW264,7, THP-1 and BV-2 inflammatory model	**Decreased** oxidative stress induced inflammatory damage	IC_{50}= 26.87±0.55 - 21.70±1.94 µM vs IC_{50}=19.37±0.72 - 15.20±1.10 µM Indomethacin control	AI	*Hypericum sampsonii* Hance, WP, EtOH	[136]
**Flavonoid**	Quercetin-3-*O*-arabinoside	Cell viability, Oxidative stress	*In vitro* A375, MDA-MB-231, SiHa, SHSY-5Y cancer cell lines; LPS stimulated RAW264,7, THP-1 and BV-2 inflammatory model	**Decreased** oxidative stress induced inflammatory damage	IC_{50}= 42.75±1.16 - 31.82±0.34 µM vs IC_{50}=19.37±0.72 - 15.20±1.10 µM Indomethacin control	AI	*Hypericum sampsonii* Hance, WP, EtOH	[136]

Compound Class	Compound	Measurement	Method	Outcome		Therapeutic Application	Species, Plant Part and Extract	Ref #
**Flavonoid**	Rutin (Quercetin 3-rutinoside)	Cell viability, Oxidative stress	*In vitro* A375, MDA-MB-231, SiHa, SHSY-5Y cancer cell lines; LPS stimulated RAW264,7, THP-1 and BV-2 inflammatory model	**Decreased** oxidative stress induced inflammatory damage	IC_{50}= 34.20±0.57 - 27.17±0.66 μM vs IC_{50}=19.37±0.72 - 15.20±1.10 μM Indomethacin control	AI	*Hypericum sampsonii* Hance, WP, EtOH	[136]
**Flavonoid**	Quercitrin	Cell viability, Oxidative stress	*In vitro* A375, MDA-MB-231, SiHa, SHSY-5Y cancer cell lines; LPS stimulated RAW264,7, THP-1 and BV-2 inflammatory model	**Decreased** oxidative stress induced inflammatory damage	IC_{50}= 38.71±1.06 - 30.66±2.29 μM vs IC_{50}=19.37±0.72 - 15.20±1.10 μM Indomethacin control	AI	*Hypericum sampsonii* Hance, WP, EtOH	[136]
**Flavonoid**	Kaempferol	Cell viability, Oxidative stress	*In vitro* A375, MDA-MB-231, SiHa, SHSY-5Y cancer cell lines; LPS stimulated RAW264,7, THP-1 and BV-2 inflammatory model	**Decreased** oxidative stress induced inflammatory damage	IC_{50}= 29.57±0.82 - 23.50±1.32 μM vs IC_{50}=19.37±0.72 - 15.20±1.10 μM Indomethacin control	AI	*Hypericum sampsonii* Hance, WP, EtOH	[136]

Compound Class	Compound	Measurement	Method	Outcome		Therapeutic Application	Species, Plant Part and Extract	Ref #
**Flavonoid**	3,8''-biapigenin	Cell viability, Oxidative stress	*In vitro* A375, MDA-MB-231, SiHa, SHSY-5Y cancer cell lines; LPS stimulated RAW264,7, THP-1 and BV-2 inflammatory model	**Decreased** cell viability (selective cytotoxicity) **Decreased** oxidative stress induced inflammatory damage	IC_{50}= 133.50±2.49 - 37.45±0.52 µM vs IC_{50}=15.74±0.13 - 5.14±0.16 µM Cisplatin control IC_{50}= 25.34±0.40 - 19.05±0.68 µM vs IC_{50}=19.37±0.72 - 15.20±1.10 µM Indomethacin control	AC AI	*Hypericum sampsonii* Hance, WP, EtOH	[136]
**Flavonoid**	Naringenin	Cell viability, Oxidative stress	*In vitro* A375, MDA-MB-231, SiHa, SHSY-5Y cancer cell lines; LPS stimulated RAW264,7, THP-1 and BV-2 inflammatory model	**Decreased** cell viability (selective cytotoxicity) **Decreased** oxidative stress induced inflammatory damage	IC_{50}= >200 - 41.70±3.26 µM vs IC_{50}=15.74±0.13 - 5.14±0.16 µM Cisplatin control IC_{50}= 31.16±0.71 - 25.51±0.89 µM vs IC_{50}=19.37±0.72 - 15.20±1.10 µM Indomethacin control	AC AI	*Hypericum sampsonii* Hance, WP, EtOH	[136]
**Flavonoid**	(+)-catechin	Cell viability, Oxidative stress	*In vitro* A375, MDA-MB-231, SiHa, SHSY-5Y cancer cell lines; LPS stimulated RAW264,7, THP-1 and BV-2 inflammatory model	**Decreased** oxidative stress induced inflammatory damage	IC_{50}= 33.20±0.61 - 25.31±0.40 µM vs IC_{50}=19.37±0.72 - 15.20±1.10 µM Indomethacin control	AI	*Hypericum sampsonii* Hance, WP, EtOH	[136]
**Flavonoid**	Myricetin	Pancreatic lipase activity	*In vitro* pancreatic lipase model	**Decreased** pancreatic lipase activity	IC_{50}= 6.94±1.40 µM vs IC_{50}=2.90±0.98 Isoginkgetin control vs IC_{50}=0.75 nM Orlistat control	Ad.In	*Hypericum* spp., -, -	[148]

Compound Class	Compound	Measurement	Method	Outcome		Therapeutic Application	Species, Plant Part and Extract	Ref #
**Flavonoid**	Luteolin	Pancreatic lipase activity	*In vitro* pancreatic lipase model	**Decreased** pancreatic lipase activity	IC_{50}= 3.43±0.24 µM vs IC_{50}=2.90±0.98 Isoginkgetin control vs IC_{50}=0.75 nM Orlistat control	Ad.In	*Hypericum* spp., -, -	[148]
**Flavonoid**	Apigenin	Pancreatic lipase activity	*In vitro* pancreatic lipase model	**Decreased** pancreatic lipase activity	IC_{50}= 2.99±0.26 µM vs IC_{50}=2.90±0.98 Isoginkgetin control vs IC_{50}=0.75 nM Orlistat control	Ad.In	*Hypericum* spp., -, -	[148]
**Flavonoid**	Procyanidin	Pancreatic lipase activity	*In vitro* pancreatic lipase model	**Decreased** pancreatic lipase activity	IC_{50}= 3.14±0.32 µM vs IC_{50}=2.90±0.98 Isoginkgetin control vs IC_{50}=0.75 nM Orlistat control	Ad.In	*Hypericum* spp., -, -	[148]
**Flavonoid**	Hyperoside (Quercetin-3-O-β-D-galactopyranoside)	Cell viability	*In vitro/ In vivo* T790M-+ Non small cells lung cancer model	**Decreased** cell proliferation **Increased** apoptosis **Decreased** xenograft growth *in vivo*	**Decreased** CCAT1 expression **Increased** FoxO1 expression	AC	*Hypericum* spp., -, -	[177]
**Flavonoid**	Quercetin	GABA levels, depressive behaviours	*In vivo* rat induced anxiety model	**Increased** GABA levels **Decreased** depressive behaviours, heme oxygenase 1 immunoreactivity		AD	*Hypericum* spp., -, -	**[178]**
**Flavonoid**	Quercetin 3-(6′′-O-caffeoyl)-β-3-D-galactoside	COX-2 Activity	*In vitro* COX-2 model	**Decreased** COX-2 activity	IC_{50}= 0.220±0.006µM vs IC_{50}=0.016±0.002µM celecoxib control	AI	*Hypericum monogynum* L., Fl, MeOH	**[179]**

Compound Class	Compound	Measurement	Method	Outcome		Therapeutic Application	Species, Plant Part and Extract	Ref #
**Flavonoid**	3,8''-biapigenin	COX-2 Activity	*In vitro* COX-2 model	**Decreased** COX-2 activity	IC_{50}= 1.655±0.098μM vs IC_{50}=0.016±0.002μM celecoxib control	AI	*Hypericum monogynum* L., Fl, MeOH	**[179]**
**Flavonoid**	Quercetin-3-O-α-L-rhamnoside	COX-2 Activity	*In vitro* COX-2 model	**Decreased** COX-2 activity	IC_{50}= 0.260±0.028μM vs IC_{50}=0.016±0.002μM celecoxib control	AI	*Hypericum monogynum* L., Fl, MeOH	**[179]**
**Flavonoid**	Taxifoline-3-O-rhamnoside	COX-2 Activity	*In vitro* COX-2 model	**Decreased** COX-2 activity	IC_{50}= 0.265±0.024μM vs IC_{50}=0.016±0.002μM celecoxib control	AI	*Hypericum monogynum* L., Fl, MeOH	**[179]**
**Flavonol**	Hyperoside (Quercetin-3-O-β-D-galactopyranoside)	Cell viability	*In vitro* LPS-induced HT22 cells	**Increased** cell viability; Il-1β, IL-6, IL-8, TNFα, ROS, MDA, Bax, caspase-3 activity; Increased CAT, SOD, GSH, Bcl-2, BDNF, TrkB, NGF expression	**Decreased** oxidative stress, LPS-induced inflammation, oxidative stress, apoptosis	CP	*Hypericum* spp., -, -	**[180]**
**Flavonol**	Quercetin	Cell viability	*In vitro* HUVEC cells<br>*In vivo* zebrafish model	**Decreased** angiogenesis; cell proliferation and survival<br>**Increased** apoptosis	**Decreased** VEGFR2 phosphorylation	AC	*Hypericum attenuatum* Fisch. ex Choisy, -, -	[181]

Compound Class	Compound	Measurement	Method	Outcome		Therapeutic Application	Species, Plant Part and Extract	Ref #
**Flavonol**	Hyperoside (Quercetin-3-O-β-D-galactopyranoside)	Stress-induced depressive behaviours	*In vivo* mouse model	**Decreased** immobility time in FST; crossing, rearing and grooming in open field test	**Increased** D2-like receptors activation	AD	*Hypericum caprifoliatum* Cham. & Schltdl., AP, MeOH	[182]
**Flavonol**	Quercetin	HIV-1 reverse transcriptase associated functions	*In vitro* Vero-76, A549, HepG2, HeLa, TZM-bl, T-lymphoid Jurkat cells	**Decreased** viral RNAse H and RDDP activity **Decreased** HIV-1 infected cells viability, HIV-1 replication	RNAse H IC_{50}=4.5±0.5μM vs IC_{50}=8.1±2.2μM RDS1643 control vs IC_{50}=12±3μM K-49 control RDDP IC_{50}=21±2μM vs IC_{50}=0.013±0.004μM Efavirenz control vs IC_{50}=11±2μM K-49 control	AV	*Hypericum hircinum* L., AP, EtOH	[166]
**Flavonol**	Quercetin 7-rhamnoside	Cell viability, Oxidative stress	*In vitro* L-02 cells	**Increased** cell viability **Decreased** oxidative stress	IC_{50}=118.75μM vs IC_{50}=313.69μM BHT control against H2O2 IC_{50}=128.47μM vs IC_{50}=172.18μM Trolox control ABTS scavenging Higher FeSO4 reduction capacity vs Trolox control **Increased** SOD, CAT, GSH **Decreased** MDA, ALT, AST, LDH, TG	CP	*Hypericum japonicum* Thunb., -, Water	[183]

Compound Class	Compound	Measurement	Method	Outcome		Therapeutic Application	Species, Plant Part and Extract	Ref #
**Flavonol**	Quercetin	Cell viability	*In vitro* L-02 cells	**Increased** cell viability **Decreased** apoptosis	**Decreased** ROS production, intracellular Ca2+ accumulation, GSH depletion	CP	*Hypericum japonicum* Thunb., WP, Water	[184]
**Flavonol**	Quercetin	Tyrosinase activity	*In vitro*	**Decreased** tyrosinase activity	IC_{50} = 14.29±0.3 μM vs IC_{50}=110.4±1.96 μM Arbutin control vs IC_{50}=8.0±0.5 μM Kojic acid control Inhibition 99.7±0.28 % vs 86.01±1.6% Arbutin control vs 99.8±0.5 % Kojic acid control	Cosm	*Hypericum laricifolium* Juss., -, MeOH	[185]
**Flavonol**	Miquelianin	Expression modulation	*In vitro* SH-SY5Y cells	**Regulated** gene expression, *vs* citalopram control	**Suppressed** FKBP5 mRNA induced increase expression, CREB induced decrease expression **Increased** GRIK4 mRNA expression, VEGF mRNA expression **Decreased** NET mRNA expression	AD	*Hypericum perforatum* L., -, -	[15]
**Flavonol**	Quercetin	Radical scavenging	*In vitro* $HepG_2$ cells	Coadministration of compounds included in formulated nanoparticles showed free radicals scavenging activity		CP	*Hypericum perforatum* L., AP, MeOH	[161]

Compound Class	Compound	Measurement	Method	Outcome		Therapeutic Application	Species, Plant Part and Extract	Ref #
**Flavonol**	Hyperoside (Quercetin-3-O-β-D-galactopyranoside)	Cell viability	*In vitro* A549 cells	**Increased** apoptosis **Decreased** cell viability	Increased LC3-II expression; ERK1/2 phosphorylation Decreased Akt, mTOR, p70S6K, 4E-BP1 phosphorylation	AC	*Hypericum* spp., -, -	[186]
**Flavonol**	Hyperoside (Quercetin-3-O-β-D-galactopyranoside)	Tumour progression	*In vitro* A431, A432, HS-4 cells	**Decreased** DMBA/TPA induced epidermal thickness **Decreased** tumour size in animals treated with hyperoside	**Increased**: Bcl-2 and Bcl-xl downregulation, Bax and Bad upregulation, Cytochrome C, caspase-9, caspase-3, PTEN, Beclin-1 and LC3I/II, Phosphorylated levels of AMPK and MAPK, PARP cleavage **Decreased**: Phosphorylated levels of PI3K, AURKA, AKT and mTOR	AC	*Hypericum* spp., -, -	[187]

Compound Class	Compound	Measurement	Method	Outcome		Therapeutic Application	Species, Plant Part and Extract	Ref #
**Flavonol**	Hyperoside (Quercetin-3-O-β-D-galactopyranoside)	Tumour progression	*In vitro* A431, A432, HS-4 cells	**Reduced** viability, migration, colony formation, apoptosis and autophagy of skin cancer cells *vs* no treatment	**Increased**: apoptosis, downregulation of Bcl-2 and Bcl-xl, upregulation of Bax, Bad, Cytochrome C and Apaf-1, caspase-9, caspase-3, PARP cleavage, PTEN, Beclin-1 and LC3II Reversion of DMBA/TPA induced changes in PI3K, AKT, mTOR and AMPK **Decreased**: cell migration, p38 phosphorylation	AC	*Hypericum* spp., -, -	[187]
**Flavonol**	Hyperoside (Quercetin-3-O-β-D-galactopyranoside)	Neuronal damage, autophagy	*In vivo* mouse model	Protected against epilepsy-induced neuronal damage in the hippocampal CA3 region. Enhanced antioxidant levels and reduced levels of autophagy related proteins *vs* kainic acid	**Decreased**: LCI/II, Autophagy-related proteins, Beclin1, PI3K, AKT, MAPK, Overactivation of microglia and astrocytes **Increased**: SOD1, SOD2, DAPI neurons	AEp	*Hypericum* spp., -, -	[188]

Compound Class	Compound	Measurement	Method	Outcome		Therapeutic Application	Species, Plant Part and Extract	Ref #
**Flavonol**	Hyperoside (Quercetin-3-O-β-D-galactopyranoside)	Cell viability, Oxidative stress	*In vivo* mouse epilepsy model	**Decreased** autophagy via PI3K/Akt and MAPK pathways; Beclin1 expression; overactivation of microglia and astrocytes<br>**Increased** SOD1 and SOD2 expression	Antioxidant *vs* sham group	CP	*Hypericum* spp., -, -	[188]
**Flavonol**	Rutin (Quercetin 3-rutinoside)	DNase I activity	*In vitro*	**Decreased** DNAse I activity	DNAse I Non-competitive inhibition IC_{50}= 108.90 ± 9.73 μM vs IC_{50}= 362.95 ± 44.37 μM	CP	*Hypericum* spp., L, S, Fl, Water	[189]
**Flavonol**	Hyperoside (Quercetin-3-O-β-D-galactopyranoside)	Cell viability	*In vitro*<br>A549 cells	**Decreased** cell viability<br>**Increased** apoptosis	**Increased** MMP dissipation, Cyp C, AIF, Casp-9, Casp-3, p38MAPK and JNK phosphorylation *vs* control	AC	*Hypericum* spp., -, -	[190]
**Flavonol**	Quercitrin	Hypericin cell permeation	*In vitro*<br>CaCo-2 cells	**Improved** permeation behaviour of hypericin		AC	*Hypericum* spp., -, -	[191]
**Flavonol**	Hyperoside (Quercetin-3-O-β-D-galactopyranoside)	Stress-induced learning and memory deficits	*In vivo* mouse model	**Reversed** depressive symptoms in forced swim test and sucrose preference test	**Increased** BDNF expression<br>**Decreased** escape latency and swimming distance *vs* fluoxetine control	AD	*Hypericum* spp., -, -	[192]

Compound Class	Compound	Measurement	Method	Outcome		Therapeutic Application	Species, Plant Part and Extract	Ref #
**Flavonol**	Hyperoside (Quercetin-3-O-β-D-galactopyranoside)	Cell viability	*In vitro* H1975 cells *In vivo* mouse model	**Decreased** cell viability, proliferation and migration **Decreased** tumour volume (*in vivo*)	**Increased** apoptosis, Bax, Bad, Bak, Cytochrome C, Apaf-1, cleaved Casp-9, Casp-3. PARP **Decreased** Bcl-2, Bcl-xl, NF-κB	AC	*Hypericum* spp., -, -	[193]
**Flavonol**	Hyperoside (Quercetin-3-O-β-D-galactopyranoside)	Cell viability, FLS proliferation	*In vitro* rheumatoid arhtritis FLS *In vivo* rheumatoid arhtritis mouse model	**Decreased** LPS induced rheumatoid arthritis FLSs proliferation and migration **Decreased** synovial hyperlasia, inflammatory cell infiltration, cartilage damage in collagen induced arthritis (*in vivo)*	**Decreased** LPS induced TNFα, IL-6, IL-1, MMP-9 expression; LPS induced NF-κB activation	AI	*Hypericum* spp., -, -	[194]
**Flavonol**	Hyperoside (Quercetin-3-O-β-D-galactopyranoside)	Expression modulation, Vascular inflammation	*In vitro* HUVEC cells *In vivo* mouse model	**Decreased** glucose induced vascular permeability, monocyte adhesion, CAMs expression, ROS formation, NF-κB activation	**Decreased** MCP-1, IL-8 expression, H2O2 formation, glucose induced p65 expression	ADb, AI	*Hypericum* spp., -, -	[195]

Compound Class	Compound	Measurement	Method	Outcome		Therapeutic Application	Species, Plant Part and Extract	Ref #
**Flavonol**	Quercetin	Mitochondrial dysfunction, oxidative stress, dopamine demise, programed cell death	*In vitro, Ex vivo*	**Increased** striatal nerve cells viability; ETC complex-I activity in damaged or normal dopaminergic neurons **Decreased** striatal dopamine loss and nigral GSH depletion	**Decreased** rotenone induced ROS formation, SOD activity, nigral catalase activity, NADPH-d induced activity	CP	*Hypericum* spp., -, -	[196]
**Flavonol**	Hyperoside (Quercetin-3-O-β-D-galactopyranoside)	Cell Viability	*In vitro* Aβ25–35-induced primary cultured cortical neurons	**Decreased** Aβ25–35-induced cytotoxicity and apoptosis	**Decreased** Aβ-induced mitochondrial dysfunction, ROS formation Cytochrome C release, Bad/BclXL interaction, casp-9, casp-3, PARP	Alz	*Hypericum* spp., -, -	[197]
**Flavonol**	Isoquercetin	Viral susceptibility	*In vitro* Vero, MDCK cells *In vivo* mouse model	**Decreased** viral replication and lung pathology	Viral replication inhibition: ED_{50}=1.2μM vs ED_{50}=1.4μM amantadine control vs ED_{50}=0.5μM oseltamivir control **Decreased** virus titers, IFN-γ, RANTES, iNOS expression (mice infected lungs)	AV	*Hypericum* spp., -, -	[198]
**Flavonolignan**	Cinchonain Ib	Cell viability, Oxidative stress	*In vitro* PC12, BV-2 cells	**Increased** cell viability	Cell viability: 49.70 - 84.10% vs 64.92 - 89.34 % captopril control	AI	*Hypericum elatoides* R.Keller, AP, MeOH	[164]

Compound Class	Compound	Measurement	Method	Outcome		Therapeutic Application	Species, Plant Part and Extract	Ref #
**Flavonolignan**	Cinchonain Ic	Cell viability, Oxidative stress	*In vitro* PC12, BV-2 cells	**Increased** cell viability	Cell viability: 47.87 - 83.86% vs 64.92 - 89.34 % captopril control	AI	*Hypericum elatoides* R.Keller, AP, MeOH	[164]
**Hydroxycinnamic acid derivative**	8,8-bis(dihydroconiferyl) diferulate	Bacterial susceptibility	*In vitro E. coli* (ATCC 8739 and AG102), *E. aerogenes* (ATCC 13048 and EA-CM64), *K. pneumoniae* (KP55 and ATCC 11296), *P. stuartii* (ATCC 29916, and PS2636), and *S. aureus* (ATCC 25923 and MRSA3)	**Decreased** bacterial growth	MIC range 0.5 - 2 µg/mL *vs* Chloramphenicol MIC range 2 - 128 µg/mL	AM	*Hypericum roeperianum* Schimp. ex A.Rich., B, MeOH	[199]
**Hyperforin derived compound**	Hydroxypropyl-β-cyclodextrin-tetracapped hyperforin	Wound healing	*In vitro* HaCaT cells	**Improved** wound healing	**Increased** intracellular Ca2+, ATP release	S	*Hypericum* spp., -, -	[200]

Compound Class	Compound	Measurement	Method	Outcome		Therapeutic Application	Species, Plant Part and Extract	Ref #
**Phenol**	Gallic acid	Cell viability, Oxidative stress	*In vitro* A375, MDA-MB-231, SiHa, SHSY-5Y cancer cell lines; LPS stimulated RAW264,7, THP-1 and BV-2 inflammatory model	**Decreased** cell viability (selective cytotoxicity) **Decreased** oxidative stress induced inflammatory damage	IC_{50}= >200 - 36.75±0.83 µM vs IC_{50}=15.74±0.13 - 5.14±0.16 µM Cisplatin control IC_{50}= 38.42±0.81 - 32.68±1.16 µM vs IC_{50}=19.37±0.72 - 15.20±1.10 µM Indomethacin control	AC AI	*Hypericum sampsonii* Hance, WP, EtOH	[136]
**Phenol**	Protocatechuic acid	Cell viability, Oxidative stress	*In vitro* A375, MDA-MB-231, SiHa, SHSY-5Y cancer cell lines; LPS stimulated RAW264,7, THP-1 and BV-2 inflammatory model	**Decreased** cell viability (selective cytotoxicity) **Decreased** oxidative stress induced inflammatory damage	IC_{50}= >200 - 53.73±1.26 µM vs IC_{50}=15.74±0.13 - 5.14±0.16 µM Cisplatin control IC_{50}= 30.25±0.93 - 25.91±1.50 µM vs IC_{50}=19.37±0.72 - 15.20±1.10 µM Indomethacin control	AC AI	*Hypericum sampsonii* Hance, WP, EtOH	[136]
**Phloroglucinol**	Hyperattenin L	Cell viability	*In vitro* HL-60, SMMC-7721, A-549, MCF-7, SW-480 cells	**Decreased** cell viability (selective toxicity)	IC_{50}= 15.45 - 3.86 µM vs 15.86 - 1.17 µM cis-platin control	AC	*Hypericum attenuatum* Fisch. ex Choisy, AP, EtOH	[201]
**Phloroglucinol**	Isouliginosin B	Cell viability	*In vitro* OVCAR-3, NCI-ADR/RES, UACC-62, MCF-7, 786-0,NCI-H460, PC-3, HT29 cells	**Decreased** cell proliferation (selective activity)	Mean TGI= 21.03µg/mL vs 0.88µg/mL Doxorubicine control	AC	*Hypericum brasiliense* Choisy, AP, n-Hexane	[47]

Compound Class	Compound	Measurement	Method	Outcome	Therapeutic Application	Species, Plant Part and Extract	Ref #
**Phloroglucinol**	Hyperelodione D	Cell viability, Expression modulation	*In vitro* HeLa and HepG2 cell lines, RXRα expression model	**Decreased** cell viability, RXRα activity	AC, IM	*Hypericum elodeoides* Choisy, WP, EtOH	[163]
**Phloroglucinol**	Hyperjaponol I	Cell viability	*In vitro* HT22 cells; DPPH free radical scavenging activity model	**Decreased** oxidative stress	CP	*Hypericum japonicum* Thunb., WP, EtOH	[62]
**Phloroglucinol**	Hyperjaponol J	Cell viability	*In vitro* HT22 cells; DPPH free radical scavenging activity model	**Decreased** induced ferroptosis	CP	*Hypericum japonicum* Thunb., WP, EtOH	[62]
**Phloroglucinol**	Hyperjaponol K	Cell viability	*In vitro* HT22 cells; DPPH free radical scavenging activity model	**Decreased** induced ferroptosis	CP	*Hypericum japonicum* Thunb., WP, EtOH	[62]
**Phloroglucinol**	3-geranyl-1+(2'-ethylyl-phloroglucinol	Cell viability	*In vitro* HT22 cells; DPPH free radical scavenging activity model	**Decreased** oxidative stress	CP	*Hypericum japonicum* Thunb., WP, EtOH	[62]
**Phloroglucinol**	Hyperjaponical C	Cell viability	*In vitro* HT22 cells; DPPH free radical scavenging activity model	**Decreased** oxidative stress	CP	*Hypericum japonicum* Thunb., WP, EtOH	[62]

Compound Class	Compound	Measurement	Method	Outcome		Therapeutic Application	Species, Plant Part and Extract	Ref #
**Phloroglucinol**	Saroaspidin B	Cell viability	*In vitro* HT22 cells; DPPH free radical scavenging activity model	**Decreased** oxidative stress		CP	*Hypericum japonicum* Thunb., WP, EtOH	[62]
**Phloroglucinol**	Saroaspidin C	Cell viability	*In vitro* HT22 cells; DPPH free radical scavenging activity model	**Decreased** induced ferroptosis		CP	*Hypericum japonicum* Thunb., WP, EtOH	[62]
**Phloroglucinol**	Sarothralin G	Cell viability	*In vitro* HT22 cells; DPPH free radical scavenging activity model	**Decreased** oxidative stress		CP	*Hypericum japonicum* Thunb., WP, EtOH	[62]
**Phloroglucinol**	Longistylione A	PTP1B Activity	*In vitro*	**Decreased** PTP1B activity	IC_{50}= 18.87±0.95 µM vs IC_{50}= 2.6±0.4 µM control	ADb	*Hypericum longistylum* Oliv., AP, MeOH	[202]
**Phloroglucinol**	Longistylione B	PTP1B Activity	*In vitro*	**Decreased** PTP1B activity	IC_{50}= 16.76±0.80 µM vs IC_{50}= 2.6±0.4 µM control	ADb	*Hypericum longistylum* Oliv., AP, MeOH	[202]
**Phloroglucinol**	Longistylione C	PTP1B Activity	*In vitro*	**Decreased** PTP1B activity	IC_{50}= 24.56±1.28 µM vs IC_{50}= 2.6±0.4 µM control	ADb	*Hypericum longistylum* Oliv., AP, MeOH	[202]

Compound Class	Compound	Measurement	Method	Outcome		Therapeutic Application	Species, Plant Part and Extract	Ref #
**Phloroglucinol**	Longistylione D	PTP1B Activity	*In vitro*	**Decreased** PTP1B activity	IC_{50}= 15.96±0.81 µM vs IC_{50}= 2.6±0.4 µM control	ADb	*Hypericum longistylum* Oliv., AP, MeOH	[202]
**Phloroglucinol**	hyperpolyphyllirin /hyperibine J	Cell viability	*In vitro* K-562, SKW-3, BV-173 cells	**Decreased** cell viability (selective activity)	IC_{50} = 21.3±1.4 - 5.3±1.5 µM	AC	*Hypericum maculatum* Crantz, AP, DCM	[203]
**Phloroglucinol**	Methoxyhyperpolyphyllirin	Cell viability	*In vitro* K-562, SKW-3, BV-173 cells	**Decreased** cell viability (selective activity)	IC_{50} = 23.5±3.5 - 4.7±1.0 µM	AC	*Hypericum maculatum* Crantz, AP, DCM	[203]
**Phloroglucinol**	Methoxyhyperibine J	Cell viability	*In vitro* K-562, SKW-3, BV-173 cells	**Decreased** cell viability (selective activity)	IC_{50} = 68.8±2.0 - 17.8±8.1 µM	AC	*Hypericum maculatum* Crantz, AP, DCM	[203]
**Phloroglucinol**	Maculatoquione A	Cell viability	*In vitro* K-562, SKW-3, BV-173 cells	**Decreased** cell viability (selective activity)	IC_{50} = 77.05±5.4 - 17.9±5.3 µM	AC	*Hypericum maculatum* Crantz, AP, DCM	[203]
**Phloroglucinol**	Maculatoquione B	Cell viability	*In vitro* K-562, SKW-3, BV-173 cells	**Decreased** cell viability (selective activity)	IC_{50} = 30.1±4.2 - 12.3±1.6 µM	AC	*Hypericum maculatum* Crantz, AP, DCM	[203]
**Phloroglucinol**	Maculatoquione C	Cell viability	*In vitro* K-562, SKW-3, BV-173 cells	**Decreased** cell viability (selective activity)	IC_{50} = 35.9±2.8 - 16.0±1.6 µM	AC	*Hypericum maculatum* Crantz, AP, DCM	[203]

Compound Class	Compound	Measurement	Method	Outcome		Therapeutic Application	Species, Plant Part and Extract	Ref #
**Phloroglucinol**	Maculatoquione D	Cell viability	*In vitro* K-562, SKW-3, BV-173 cells	**Decreased** cell viability (selective activity)	IC_{50} = 28.1±4.0 - 16.7±2.0 µM	AC	*Hypericum maculatum* Crantz, AP, DCM	[203]
**Phloroglucinol**	Erectquione A	Cell viability	*In vitro* K-562, SKW-3, BV-173 cells	**Decreased** cell viability (selective activity)	IC_{50} = 46.3±3.6 - 22.4±2.0 µM	AC	*Hypericum maculatum* Crantz, AP, DCM	[203]
**Phloroglucinol**	Hyperinakin	Cell viability	*In vitro* RAW264.7 cells	**Increased** cell viability **Decreased** NO production	IC_{50}=20µM *vs* IC_{50}=10µM Rapamycin control	AI	*Hypericum nakamurai* (Masam.) N.Robson, L, S, DCM/EtOH	[204]
**Phloroglucinol**	Revolutin	Oxidative stress, Aortic tension	*Ex vivo* aortic model, *in vitro* ROS scavenging model	**Decreased** induced vasoconstriction **Increased** NO production	**Decreased** aortic tension	Vd	*Hypericum revolutum* Vahl, AP, MeOH (Chl f)	[205]
**Phloroglucinol**	Hyperforatin F	Cell viability, Oxidative stress	*In vitro* A375, MDA-MB-231, SiHa, SHSY-5Y cancer cell lines; LPS stimulated RAW264,7, THP-1 and BV-2 inflammatory model	**Decreased** cell viability (selective cytotoxicity) **Decreased** oxidative stress induced inflammatory damage	IC_{50}= 65.92 ± 0.80 - 7.52 ± 0.24 µM vs IC_{50}=15.74 ± 0.13 - 5.14 ± 0.16 µM Cisplatin control IC_{50}= 18.05±0.46 - 13.05±0.42 µM vs IC_{50}=19.37±0.72 - 15.20±1.10 µM Indomethacin control	AC AI	*Hypericum sampsonii* Hance, WP, EtOH	[136]
**Phloroglucinol**	Isoaustrobrasilol B	Protozoal susceptibility	*In vitro Trichomonas vaginalis*	**Decreased** Trophozoites viability	**Decreased** parasitic modulation of human immune response	AP	*Hypericum* spp., AP, n-Hexane	[206]

Compound Class	Compound	Measurement	Method	Outcome		Therapeutic Application	Species, Plant Part and Extract	Ref #
**Phloroglucinol**	(-)-erectumol I	Cell viability	*In vitro* HeLa cells	**Increased** apoptosis (selective cytotoxicity)<br>**Decreased** Hsp 105 expression		AC	*Hypericum erectum* Thunb., WP, MeOH	**[207]**
**Phloroglucinol**	(-)-erectumol II	Cell viability	*In vitro* HeLa cells	**Increased** apoptosis (selective cytotoxicity)<br>**Decreased** Hsp 105 expression		AC	*Hypericum erectum* Thunb., WP, MeOH	**[207]**
**Phloroglucinol derivative**	Sampsonione J	Cell viability, Oxidative stress	*In vitro* RAW264.7 cells	**Decreased** oxidative stress induced inflammatory damage	**Decreased** NO production<br>$IC_{50} \leq 40.32$ µM	CP<br>AI	*Hypericum sampsonii* Hance, AP, MeOH	[157]
**Phloroglucinol derivative**	Otogirinin A	Cell viability, Oxidative stress	*In vitro* RAW264.7 cells	**Decreased** oxidative stress induced inflammatory damage	**Decreased** NO production, TNF-α production, iNOS expression, MAPK/JNK and IκBα phosphorylation<br>**Increased** arginase 1 and KLF4 expression<br>$IC_{50} \leq 40.32$ µM	CP<br>AI	*Hypericum sampsonii* Hance, AP, MeOH	[157]
**Pyranoxanthones**	Jacarelhyperol A	Cell viability, Tumour growth	*In vitro* MBA-MB-231, T47D, LOVO, A549, HepG2, K562, HL-60, THP-1 cells<br>*In vivo* mouse xenograph	**Decreased** cell viability (selective cytotoxicity)<br>**Increased** apoptosis<br>**Decreased** tumour growth *in vivo*	Cytotoxicity IC_{50} = 33.24±2.1 - 6.52±0.36µM vs IC_{50} = 0.68±0.02 - 0.11±0.01 µM Doxorubicin control<br>**Decreased** Bcl-Xl, Bcl-2, and Mcl-1 activity;<br>**Increased** cleaved Casp-9, cleaved Casp-3, cleaved PARP;	AC	*Hypericum japonicum* Thunb., -, -	[208]

Compound Class	Compound	Measurement	Method	Outcome		Therapeutic Application	Species, Plant Part and Extract	Ref #
**Sterol**	4,4-dimethyl cholesterol	Lipoxygenase activity	*In vitro*	**Decreased** Lypoxygenase activity	IC_{50}= 68.5 ± 0.10 µM vs IC_{50}= 22.0 ± 0.04 µM Baicalein control	AI	*Hypericum oblongifolium* Choisy, WP, MeOH (Chl f)	[167]
**Sterol**	Erectasteroid D	Lipoxygenase activity	*In vitro*	**Decreased** Lypoxygenase activity	IC_{50}= 31.0 ± 0.10 µM vs IC_{50}= 22.0 ± 0.04 µM Baicalein control	AI	*Hypericum oblongifolium* Choisy, WP, MeOH (Chl f)	[167]
**Sterol**	β-sitosterol-3-O-β-D-glucopyranoside	Lipoxygenase activity	*In vitro*	**Decreased** Lypoxygenase activity	IC_{50}= 39.3 ± 0.10 µM vs IC_{50}= 22.0 ± 0.04 µM Baicalein control	AI	*Hypericum oblongifolium* Choisy, WP, MeOH (Chl f)	[167]
**Stigmastane sterol**	β-sitosterol	Oxidative stress, Aortic tension	*Ex vivo* aortic model, *in vitro* ROS scavenging model	**Decreased** induced vasoconstriction **Increased** NO production	**Decreased** aortic tension	Vd	*Hypericum revolutum* Vahl, AP, MeOH (Chl f)	[205]
**Terpene**	Hypatulin A	Bacterial susceptibility	*In vitro Bacillus subtilis*	Active against *B. subtilis*	MIC=16 µg/mL	AM	*Hypericum patulum* Thunb., L, MeOH	[209]
**Terpene**	Dihydrovomifoliol -O-b-D-glucopyranoside	Cell viability, oxidative stress	*In vitro* BV-2 cell lines	**Decreased** LPS induced NO production	IC_{50}= 1.28±0.15 µM vs IC_{50}=4.00±0.23µM Quercetin control	CP	*Hypericum przewalskii* Maxim., AP, MeOH	[154]
**Terpene**	Hyperevolutin C	Oxidative stress, Aortic tension	*Ex vivo* aortic model, *in vitro* ROS scavenging model	**Decreased** induced vasoconstriction **Increased** NO production	**Decreased** aortic tension	Vd	*Hypericum revolutum* Vahl, AP, MeOH (Chl f)	[205]

Compound Class	Compound	Measurement	Method	Outcome		Therapeutic Application	Species, Plant Part and Extract	Ref #
**Terpene derivative**	Hyperterpenoid A	Cell viability	*In vitro* SK-N-SH cells	**Increased** cell viability<br>**Decreased** induced cell toxicity		CP	*Hypericum beanii* N. Robson, AP, MeOH	**[129]**
**Terpene derivative**	Hyperterpenoid B	Cell viability	*In vitro* SK-N-SH cells	**Increased** cell viability<br>**Decreased** induced cell toxicity		CP	*Hypericum beanii* N. Robson, AP, MeOH	**[129]**
**Terpene derivative**	Hyperdioxane A	Cell viability	*In vitro* MT-4 cells HIV model	**Decreased** HIV activity		AV	*Hypericum ascyron* L., R, MeOH	[210]
**Terpene derivative**	Hyperdioxane B	Cell viability	*In vitro* LPS-stimulated microglial cells	**Decreased** IL-1β production		CP	*Hypericum ascyron* L., R, MeOH	[210]
**Triterpene**	Acetyloleanolic acid	Cell viability	*In vitro* HL-60, SMMC-7721, A-549, MCF-7, SW480 cells	**Decreased** cell viability (selective cytotoxicity)	IC_{50} = 33.3±0.7µM - 14.8±2.8µM vs IC_{50} = 15.1±2.9µM - 1.1±0.1µM cisplatin control vs IC_{50}= <0.008 µM paclitaxel control	AC	*Hypericum androsaemum* L., AP, MeOH	[211]

Compound Class	Compound	Measurement	Method	Outcome		Therapeutic Application	Species, Plant Part and Extract	Ref #
**Triterpene**	Betulinic acid	HIV-1 reverse transcriptase associated functions	*In vitro* Vero-76, A549, HepG2, HeLa, TZM-bl, T-lymphoid Jurkat cells	**Decreased** viral RNAse H and RDDP activity (normal and mutants) **Decreased** HIV-1 infected cells viability, HIV-1 replication	RNAse H IC_{50}=2.0±0.2µM vs IC_{50}=8.1±2.2µM RDS1643 control vs IC_{50}=12±3µM K-49 control RDDP IC_{50}=0.5±0.1µM vs IC_{50}=0.013±0.004µM Efavirenz control vs IC_{50}=11±2µM K-49 control	AV	*Hypericum hircinum* L., AP, EtOH	[166]
**Triterpene**	Betulinic acid	Protozoal susceptibility	*In vitro Plasmodium falciparum*	Active against all tested strains	IC_{50}=5.60±0.13µg/mL - 4.50±1.35µg/mL vs IC_{50}=0.27±0.04µg/mL - 0.14±0.05µg/mL quinine control	Mal	*Hypericum lanceolatum* Lam., Sb, MeOH	[153]
**Triterpene**	3-oxo-20(30)-taraxastene-28,13β-olide	Lipoxygenase activity	*In vitro*	**Decreased** Lypoxygenase activity	IC_{50}= 48.7 ± 0.10 µM vs IC_{50}= 22.0 ± 0.04 µM Baicalein control	AI	*Hypericum oblongifolium* Choisy, WP, MeOH (Chl f)	[167]
**Triterpene**	Lupeol	Lipoxygenase activity	*In vitro*	**Decreased** Lypoxygenase activity	IC_{50}= 61.5 ± 0.10 µM vs IC_{50}= 22.0 ± 0.04 µM Baicalein control	AI	*Hypericum oblongifolium* Choisy, WP, MeOH (Chl f)	[167]
**Triterpene**	Taraxerol	Lipoxygenase activity	*In vitro*	**Decreased** Lypoxygenase activity	IC_{50}= 83.0 ± 0.10 µM vs IC_{50}= 22.0 ± 0.04 µM Baicalein control	AI	*Hypericum oblongifolium* Choisy, WP, MeOH (Chl f)	[167]

Compound Class	Compound	Measurement	Method	Outcome		Therapeutic Application	Species, Plant Part and Extract	Ref #
**Triterpene**	Oleanolic acid	Lipoxygenase activity	*In vitro*	**Decreased** Lypoxygenase activity	IC_{50}= 68.5 ± 0.10 µM vs IC_{50}= 22.0 ± 0.04 µM Baicalein control	AI	*Hypericum oblongifolium* Choisy, WP, MeOH (Chl f)	[167]
**Triterpene**	Trichadonic acid	Cell viability	*In vitro* CCRF-CEM, CEM/ADR5000, U87.MG, U87.MGΔEGFR, HCT116($p53^{+/+}$), HCT116($p53^{-/-}$), MDA-MB-231-pcDNA3 and MDA-MB-231-BCRP cancer cell lines	**Increased** apoptosis, ROS formation, caspase activation **Decreased** cell viability (selective cytotoxicity)	IC_{50}= 47.34±0.81 - 14.44±0.53 µM vs IC_{50}=122.96±10.94 - 0.02±0.00 µM Doxorubicin control	AC	*Hypericum roeperianum* Schimp. ex A.Rich., B, MeOH	[212]
**Triterpene**	Triterhyper A	Cell proliferation	*Ex vivo* murine splenocytes model	**Decreased** anti-CD3/anti-CD28 and LPS induced cell proliferation			*Hypericum longistylum* Oliv., AP, EtOH	**[213]**

Compound Class	Compound	Measurement	Method	Outcome		Therapeutic Application	Species, Plant Part and Extract	Ref #
**Triterpene**	Betulin 3-acetate	Cell proliferation	*Ex vivo* murine splenocytes model	**Decreased** anti-CD3/anti-CD28 and LPS induced cell proliferation		IM	*Hypericum longistylum* Oliv., AP, EtOH	**[213]**
**Triterpene**	Lupeol	Cell proliferation	*Ex vivo* murine splenocytes model	**Decreased** anti-CD3/anti-CD28 and LPS induced cell proliferation		IM	*Hypericum longistylum* Oliv., AP, EtOH	**[213]**
**Unspecified**	p27SJ/p38SJ	Oxidative stress	*In vitro* Human primary cortical neurons	**Decreased** Tat induced oxidative stress, mithocondrial permeability	**Increased** Cdk5 and MAPK hyperphosphorylation **Decreased** Tat induced MEF2 hyperphosphorylation	CP	*Hypericum* spp., -, -	[214]
**Unspecified**	1,4-O-diferuloylsecoisolariciresinol	Cell viability	*In vitro* HL-60, SMMC-7721, A-549, MCF-7, SW480 cells	**Decreased** cell viability (selective cytotoxicity)	IC_{50} = 15.3±1.1µM - 4.5±0.1µM vs IC_{50} = 15.1±2.9µM - 1.1±0.1µM cisplatin control vs IC_{50}= <0.008 µM paclitaxel control	AC	*Hypericum androsaemum* L., AP, MeOH	[211]

Compound Class	Compound	Measurement	Method	Outcome		Therapeutic Application	Species, Plant Part and Extract	Ref #
**Unspecified**	Tenuiside A	Cell viability, Oxidative stress	*In vitro*<br>PC12, BV-2 cells	**Increased** cell viability<br>**Decreased** NO LPS induced production	Cell viability: 52.09 - 85.12% vs 64.92 - 89.34 % captopril control<br>IC_{50}= 1.39 ± 0.03 µM vs IC_{50}= 1.07 ± 0.04 µM Quercetin control vs IC_{50}= 2.6 ± 0.06 µM Fluoxetine control	AI	*Hypericum elatoides* R.Keller, AP, MeOH	[164]
**Unspecified**	(Z)-3-hexenyl-β-D-glucopyranoside 18	Cell viability, Oxidative stress	*In vitro*<br>PC12, BV-2 cells	**Decreased** NO LPS induced production	IC_{50}= 2.95 ± 0.07 µM vs IC_{50}= 1.07 ± 0.04 µM Quercetin control vs IC_{50}= 2.6 ± 0.06 µM Fluoxetine control	AI	*Hypericum elatoides* R.Keller, AP, MeOH	[164]
**Unspecified**	4,6-dihydroxy-2-methoxyphenyl-1-O-β-D-glucopyranoside	Bacterial susceptibility	*In vitro*<br>*Helicobacter pylori*	Antimicrobial activity against all tested strains	MIC = 58.2µg/mL - 7.3µg/mL vs MIC = 0.042µg/mL - 0.037µg/mL Clarithromycin control vs MIC = 0.18µg/mL - 0.002µg/mL Amoxicillin control	AM	*Hypericum erectum* Thunb., WP, MeOH	[215]
**Unspecified**	4-hydroxy-2,6-dimethoxyphenyl-1-O-α-L-rhamnopyranosyl (1–6)-β-D-glucopyranoside	Bacterial susceptibility	*In vitro*<br>*Helicobacter pylori*	Selective antimicrobial activity	MIC= 27.3µg/mL vs MIC=0.037µg/mL Clarithromycin control vs MIC=0.18µg/mL Amoxicillin control	AM	*Hypericum erectum* Thunb., WP, MeOH	[215]
**Unspecified**	(+) Japonone A	Viral susceptibility	*In vitro*<br>iSLK.219 rKSHV.219-infected cells	Active against Kaposi's sarcoma associated herpesvirus	EC_{50}=166.00 µM; Selectivity index higher than 3.01	AV	*Hypericum japonicum* Thunb., AP, EtOH	[216]

Compound Class	Compound	Measurement	Method	Outcome		Therapeutic Application	Species, Plant Part and Extract	Ref #
**Unspecified**	HLT0 **UNIDENTIFIED**	Protozoal susceptibility	*In vitro Plasmodium falciparum*	Active against all tested strains	IC_{50}=5.89±0.20µg/mL - 4.26±0.15µg/mL vs IC_{50}=0.27±0.04µg/mL - 0.14±0.05µg/mL quinine control	Mal	*Hypericum lanceolatum* Lam., Sb, MeOH	[153]
**Unspecified**	4,4-dimethylergosta-8,14,24(28)-triene-3β,12β,17α-triol	Lipoxygenase activity	*In vitro*	**Decreased** Lypoxygenase activity	IC_{50}= 71.0 ± 0.10 µM vs IC_{50}= 22.0 ± 0.04 µM Baicalein control	AI	*Hypericum oblongifolium* Choisy, WP, MeOH (Chl f)	[167]
**Unspecified**	Unnamed compound	Antimicrobial activity	*In vitro*	Unidentified compound	Unidentified compound	AM	*Hypericum olympicum* L. cf. uniflorum, AP, n-Hexane	[217]
**Unspecified**	Eleocharin A	NO production	*In vitro* DLD-1 cells, RAW264.4 cells, IMR-32 cells	Decreased NO production<br>Decreased DLD-1 cell viability<br>Decreased IMR-1 cell viability	IC_{50}= 10.52µM<br>IC_{50}= 5.98µM<br>IC_{50}= 4.79µM	AC, CP	Hypericum nokoense Ohwi, AP, MeOH	**[168]**
**Xanthone**	Ananixanthone	Cell viability	*In vitro* HUVEC cells	**Decreased** induced cell damage		CP	*Hypericum acmosepalum* N.Robson, AP, EtOH	[218]
**Xanthone**	Osajaxanthone	Cell viability	*In vitro* HUVEC cells	**Decreased** induced cell damage		CP	*Hypericum acmosepalum* N.Robson, AP, EtOH	[218]

Compound Class	Compound	Measurement	Method	Outcome		Therapeutic Application	Species, Plant Part and Extract	Ref #
**Xanthone**	Mangiferin	Stress-induced depressive behaviours MAO activity	*In vivo* mouse model	**Decreased** immobility time in FST	FST time of immobility: 159.5±18.2 - 80.5±42.4 (single dose dependent) vs 108±17.2 Imipramine control **Decreased** MAO activity	AD	*Hypericum aucheri* Jaub. & Spach, AP, EtOH	[219]
**Xanthone**	Hyperelatone G	Cell viability, Oxidative stress	*In vitro* PC12, BV-2 cells	**Increased** cell viability **Decreased** NO LPS induced production	Cell viability: 50.72 - 80.20% vs 64.92 - 89.34 % captopril control IC_{50}= 3.84 ± 0.15 µM vs IC_{50}= 1.07 ± 0.04 µM Quercetin control vs IC_{50}= 2.6 ± 0.06 µM Fluoxetine control	AI	*Hypericum elatoides* R.Keller, AP, MeOH	[164]
**Xanthone**	Hyperelatone H	Cell viability, Oxidative stress	*In vitro* PC12, BV-2 cells	**Increased** cell viability **Decreased** NO LPS induced production	Cell viability: 63.17 - 90.08% vs 64.92 - 89.34 % captopril control IC_{50}= 0.75 ± 0.02 µM vs IC_{50}= 1.07 ± 0.04 µM Quercetin control vs IC_{50}= 2.6 ± 0.06 µM Fluoxetine control	AI	*Hypericum elatoides* R.Keller, AP, MeOH	[164]
**Xanthone**	1,3,6-trihydroxy-7-O-(3-methylbut-2-enyl) xanthone	RXRα transcription Cell viability	*In vitro* 293T, HeLa cells	**Induced** apoptosis **Inhibited** HeLa cells proliferation (selectively), RXRα transcription	**Increased**: caspase-8 activation, PARP cleavage	AC	*Hypericum elodeoides* Choisy, WP, EtOH	[163]
**Xanthone**	Hyperfaberol A	Cell viability	*In vitro* ECA-109 cells	Cytotoxicity against ECA-109	IC_{50}= 12.8µM vs IC_{50}= 1.2 µM Taxol control	AC	*Hypericum faberi* R.Keller, WP, MeOH	[52]

Compound Class	Compound	Measurement	Method	Outcome		Therapeutic Application	Species, Plant Part and Extract	Ref #
**Xanthone**	Isojacareubin	Bacterial susceptibility	*In vitro* MRSA	Active against all tested strains	MIC_{50}=8µM *vs* MIC_{50}=64µM Ampicillin control *vs* MIC_{50}=512µM Ceftazidime control *vs* MIC_{50}=16µM Levofloxacin control	AM	*Hypericum japonicum* Thunb., AP, EtOH	[220]
**Xanthone**	Kellerine A	Cell viability	*In vitro* HeLa cells	**Decreased** cell viability	IC_{50}=2.5±0.1 µM	AC	*Hypericum kelleri* Bald., AP, Cyclohexane	[221]
**Xanthone**	Kellerine B	Cell viability	*In vitro* HeLa cells	**Decreased** cell viability	IC_{50}=5.9±0.9 µM	AC	*Hypericum kelleri* Bald., AP, Cyclohexane	[221]
**Xanthone**	5-Hydroxy-3-methoxyxanthone	Protozoal susceptibility	*In vitro Plasmodium falciparum*	Active against all tested strains	IC_{50}=3.26±0.08µg/mL - 1.43±0.48µg/mL vs IC_{50}=0.27±0.04µg/mL - 0.14±0.05µg/mL quinine control	Mal	*Hypericum lanceolatum* Lam., Sb, MeOH	[153]
**Xanthone**	3-Hydroxy-5-methoxyxanthone	Protozoal susceptibility	*In vitro Plasmodium falciparum*	Active against all tested strains	IC_{50}=34.09±0.12µg/mL - 33.84±0.20µg/mL vs IC_{50}=0.27±0.04µg/mL - 0.14±0.05µg/mL quinine control	Mal	*Hypericum lanceolatum* Lam., Sb, MeOH	[153]
**Xanthone**	5-O-methyl-2-deprenylrheediaxanthone B	MHC inhibition	*In vitro*	**Decreased** MHC expression	Decreased MICA (24%). HLA-E (40%). HLA-DR (25%) expression vs control	IM	*Hypericum perforatum* L., R, Cyclohexane	[222]

Compound Class	Compound	Measurement	Method	Outcome		Therapeutic Application	Species, Plant Part and Extract	Ref #
**Xanthone**	Biyouxanthone D	Bacterial susceptibility	*In vitro Cryptococcus neoformans* and dermatophytes	Active against all tested strains	MIC (C. neoformans)= 32-16μg/mL vs 4-1μg/mL fluconazol control MIC (Dermatophytes)= 32-16μg/mL vs 16-1μg/mL fluconazol control	AF	*Hypericum perforatum* subsp. *veronense* (Schrank) H.Lindb, In vitro R, Chloroform	[223]
**Xanthone**	2,3,4-trimethoxy xanthone	Oxidative stress, Aortic tension	*Ex vivo* aortic model, *in vitro* ROS scavenging model	**Decreased** induced vasoconstriction**,** ROS production	**Decreased** aortic tension**,** oxidative stress	CP	*Hypericum revolutum* Vahl, AP, MeOH (Chl f)	[205]
**Xanthone**	2-hydroxy-5-methoxyxanthone	Cell viability	*In vitro* CCRF-CEM, CEM/ADR5000, U87.MG, U87.MGΔEGFR, HCT116(p53$^{+/+}$), HCT116(p53$^{-/-}$), MDA-MB-231-pcDNA3 and MDA-MB-231-BCRP cancer cell lines	**Decreased** cell viability (selective cytotoxicity)	IC_{50}= >165.29 - 16.80±0.96 µM vs IC_{50}=122.96±10.94 - 0.02±0.00 µM Doxorubicin control	AC	*Hypericum roeperianum* Schimp. ex A.Rich., B, MeOH	[212]

Compound Class	Compound	Measurement	Method	Outcome		Therapeutic Application	Species, Plant Part and Extract	Ref #
**Xanthone**	Norathyriol	Cell viability	*In vitro* CCRF-CEM, CEM/ADR5000, U87.MG, U87.MGΔEGFR, HCT116(p53$^{+/+}$), HCT116(p53$^{-/-}$), MDA-MB-231-pcDNA3 and MDA-MB-231-BCRP cancer cell lines	**Decreased** cell viability (selective cytotoxicity)	IC_{50}= >153.85 - 19.94±2.12 µM vs IC_{50}=122.96±10.94 - 0.02±0.00 µM Doxorubicin control	AC	*Hypericum roeperianum* Schimp. ex A.Rich., B, MeOH	[212]
**Xanthone**	1,3,5,6-tetrahydroxyxanthone	Cell viability	*In vitro* CCRF-CEM, CEM/ADR5000, U87.MG, U87.MGΔEGFR, HCT116(p53$^{+/+}$), HCT116(p53$^{-/-}$), MDA-MB-231-pcDNA3 and MDA-MB-231-BCRP cancer cell lines	**Decreased** cell viability (selective cytotoxicity)	IC_{50}= 150.02±7.03 - 38.46±4.07 µM vs IC_{50}=122.96±10.94 - 0.02±0.00 µM Doxorubicin control	AC	*Hypericum roeperianum* Schimp. ex A.Rich., B, MeOH	[212]
**Xanthone**	1,4,6,7-tetrahydroxyxanthone	Bacterial susceptibility	*In vitro E. coli* ATCC8739, *K. pneumoniae* KP55, *Enterobacter cloacae* ATCC13048	**Decreased** bacterial growth	MIC range 2 - 64 µg/mL	AM	*Hypericum roeperianum* Schimp. ex A.Rich., B, MeOH	[224]

Compound Class	Compound	Measurement	Method	Outcome		Therapeutic Application	Species, Plant Part and Extract	Ref #
**Xanthone**	1,5-dihydroxy-6-methoxyxanthone	Bacterial susceptibility	*In vitro E. coli* ATCC8739, *K. pneumoniae* KP55, *Enterobacter cloacae* ATCC13048	**Decreased** bacterial growth	MIC range 8 - 128 µg/mL	AM	*Hypericum roeperianum* Schimp. ex A.Rich., B, MeOH	[224]
**Xanthone**	2-methoxy-9H-xanthen-9-one	Cell viability, Oxidative stress	*In vitro* A375, MDA-MB-231, SiHa, SHSY-5Y cancer cell lines; LPS stimulated RAW264,7, THP-1 and BV-2 inflammatory model	**Decreased** oxidative stress induced inflammatory damage	IC_{50}= 37.64±1.32 - 31.76±2.50 µM vs IC_{50}=19.37±0.72 - 15.20±1.10 µM Indomethacin control	AI	*Hypericum sampsonii* Hance, WP, EtOH	[136]
**Xanthone**	1-hydroxy-7-methoxy- 9H-xanthen-9-one	Cell viability, Oxidative stress	*In vitro* A375, MDA-MB-231, SiHa, SHSY-5Y cancer cell lines; LPS stimulated RAW264,7, THP-1 and BV-2 inflammatory model	**Decreased** oxidative stress induced inflammatory damage	IC_{50}= 28.03±1.24 - 24.32±1.09 µM vs IC_{50}=19.37±0.72 - 15.20±1.10 µM Indomethacin control	AI	*Hypericum sampsonii* Hance, WP, EtOH	[136]

Compound Class	Compound	Measurement	Method	Outcome		Therapeutic Application	Species, Plant Part and Extract	Ref #
**Xanthone**	1,7-dihydroxy-9H-xanthen-9-one	Cell viability, Oxidative stress	*In vitro* A375, MDA-MB-231, SiHa, SHSY-5Y cancer cell lines; LPS stimulated RAW264,7, THP-1 and BV-2 inflammatory model	**Decreased** oxidative stress induced inflammatory damage	IC_{50}= 35.89±0.90 - 29.07±0.87 µM vs IC_{50}=19.37±0.72 - 15.20±1.10 µM Indomethacin control	AI	*Hypericum sampsonii* Hance, WP, EtOH	[136]
**Xanthone**	5-O-methyl-2-deprenylrheediax anthone B	Cell viability, Oxidative stress	*In vitro* A375, MDA-MB-231, SiHa, SHSY-5Y cancer cell lines; LPS stimulated RAW264,7, THP-1 and BV-2 inflammatory model	**Decreased** cell viability (selective cytotoxicity) **Decreased** oxidative stress induced inflammatory damage	IC_{50}= >200 - 32.20±0.63 µM vs IC_{50}=15.74±0.13 - 5.14±0.16 µM Cisplatin control IC_{50}= 22.03±0.72 - 18.92±1.53 µM vs IC_{50}=19.37±0.72 - 15.20±1.10 µM Indomethacin control	AC AI	*Hypericum sampsonii* Hance, WP, EtOH	[136]
**Xanthone**	5'-demethoxycadensin G	Cell viability, Oxidative stress	*In vitro* A375, MDA-MB-231, SiHa, SHSY-5Y cancer cell lines; LPS stimulated RAW264,7, THP-1 and BV-2 inflammatory model	**Decreased** cell viability (selective cytotoxicity) **Decreased** oxidative stress induced inflammatory damage	IC_{50}=158.90±0.59 - 36.52±0.62 µM vs IC_{50}=15.74±0.13 - 5.14±0.16 µM Cisplatin control IC_{50}= 30.01±0.64 - 22.32±0.73 µM vs IC_{50}=19.37±0.72 - 15.20±1.10 µM Indomethacin control	AC AI	*Hypericum sampsonii* Hance, WP, EtOH	[136]

Compound Class	Compound	Measurement	Method	Outcome		Therapeutic Application	Species, Plant Part and Extract	Ref #
**Xanthone**	Jacareubin	Cell viability, Oxidative stress	*In vitro* A375, MDA-MB-231, SiHa, SHSY-5Y cancer cell lines; LPS stimulated RAW264,7, THP-1 and BV-2 inflammatory model	**Decreased** cell viability (selective cytotoxicity) **Decreased** oxidative stress induced inflammatory damage	IC_{50}= >200 - 36.64±0.67 µM vs IC_{50}=15.74±0.13 - 5.14±0.16 µM Cisplatin control IC_{50}= 26.65±1.03 - 20.71±1.58 µM vs IC_{50}=19.37±0.72 - 15.20±1.10 µM Indomethacin control	AC AI	*Hypericum sampsonii* Hance, WP, EtOH	[136]
**Xanthone**	2-hydroxy-3-methoxyxanthone	Cell viability	*In vitro* SMMC-7721, Huh-7, HepG2, SK-HEP-1, PLC/PRF/5, LO2 cells	Selective cytotoxicity against HepG2 and LO2	IC_{50}= 10.19±0.12 µM (HepG2). 14.47±0.95 µM (LO2) vs IC_{50}=20.62±1.03 µM - 4.47±0.27 µM cisplatin control vs IC_{50}= 0.18± 0.03 - <0.01 µM taxol control	AC	*Hypericum stellatum* N.Robson, AP, EtOH	[225]
**Xanthone**	1,3,8-trihydroxyxanthone	Cell viability	*In vitro* SMMC-7721, Huh-7, HepG2, SK-HEP-1, PLC/PRF/5, LO2 cells	Selective cytotoxicity against SMMC-7721 Weak cytotoxicity against LO2	IC_{50}= 15.20±0.27 µM (SMMC-7721). >40 µM (LO2) vs IC_{50}=20.62±1.03 µM - 4.47±0.27 µM cisplatin control vs IC_{50}= 0.18± 0.03 - <0.01 µM taxol control	AC	*Hypericum stellatum* N.Robson, AP, EtOH	[225]
**Xanthone**	1,3,7-trihydroxyxanthone	Cell viability	*In vitro* SMMC-7721, Huh-7, HepG2, SK-HEP-1, PLC/PRF/5, LO2 cells	Selective cytotoxicity against HepG2 Weak cytotoxicity against LO2	IC_{50}= 22.60±1.43 µM (HepG2). >40 µM (LO2) vs IC_{50}=20.62±1.03 µM - 4.47±0.27 µM cisplatin control vs IC_{50}= 0.18± 0.03 - <0.01 µM taxol control	AC	*Hypericum stellatum* N.Robson, AP, EtOH	[225]

Compound Class	Compound	Measurement	Method	Outcome		Therapeutic Application	Species, Plant Part and Extract	Ref #
**Xanthone**	Isojacareubin	Cell viability	*In vitro* SMMC-7721, Huh-7, HepG2, SK-HEP-1, PLC/PRF/5, LO2 cells	**Decreased** cell viability (selective activity)	IC_{50}=11.83±0.56 µM - 1.41±0.03 µM IC_{50}=20.62±1.03 µM - 4.47±0.27 µM cisplatin control vs IC_{50}= 0.18± 0.03 - <0.01 µM taxol control	AC	*Hypericum stellatum* N.Robson, AP, EtOH	[225]
**Xanthone**	1,3,7-trihydroxy-6-methoxyxanthone	Cell viability	*In vitro* SMMC-7721, Huh-7, HepG2, SK-HEP-1, PLC/PRF/5, LO2 cells	Selective cytotoxicity SMMC-7721, SK-HEP-1 and LO2	IC_{50}=37.09±0.97 µM - 12.09±0.14 µM IC_{50}=20.62±1.03 µM - 4.47±0.27 µM cisplatin control vs IC_{50}= 0.18± 0.03 - <0.01 µM taxol control	AC	*Hypericum stellatum* N.Robson, AP, EtOH	[225]
**Xanthone**	Hypxanthones A	Cell viability	*In vitro* SMMC-7721, Huh-7, HepG2, SK-HEP-1, PLC/PRF/5, LO2 cells	Selective cytotoxicity against SMMC-7721 Weak cytotoxicity against LO2	IC_{50}= 27.56±0.68 µM (SMMC-7721). >40 µM (LO2) vs IC_{50}=20.62±1.03 µM - 4.47±0.27 µM cisplatin control vs IC_{50}= 0.18± 0.03 - <0.01 µM taxol control	AC	*Hypericum stellatum* N.Robson, AP, EtOH	[225]
**Xanthone**	Hypxanthones B	Cell viability	*In vitro* SMMC-7721, Huh-7, HepG2, SK-HEP-1, PLC/PRF/5, LO2 cells	Selective cytotoxicity against SMMC-7721, Huh-7, HepG2, SK-HEP-1, PLC/PRF/5, LO2 Weak cytotoxicity against LO2	IC_{50}=30.76±0.38µM - 8.26±0.57 µM IC_{50}=20.62±1.03 µM - 4.47±0.27 µM cisplatin control vs IC_{50}= 0.18± 0.03 - <0.01 µM taxol control	AC	*Hypericum stellatum* N.Robson, AP, EtOH	[225]

Compound Class	Compound	Measurement	Method	Outcome		Therapeutic Application	Species, Plant Part and Extract	Ref #
**Xanthone**	Calycinoxanthon D	Cell viability	*In vitro* SMMC-7721, Huh-7, HepG2, SK-HEP-1, PLC/PRF/5, LO2 cells	Selective cytotoxicity against all tested cells	IC_{50}=31.11±2.67µM - 6.27±0.16 µM IC_{50}=20.62±1.03 µM - 4.47±0.27 µM cisplatin control vs IC_{50}= 0.18± 0.03 - <0.01 µM taxol control	AC	*Hypericum stellatum* N.Robson, AP, EtOH	[225]
**Xanthone**	Wilsonxanthone B	Glucose transporter 4 activity	*In vitro* L6 cell model	**Increased** GLUT4 translocation	vs insulin positive control	ADb	*Hypericum wilsonii N.* Robson, AP, EtOH	**[123]**
**Xanthone**	Isojacareubin	NO production	*In vitro* DLD-1 cells, RAW264.4 cells, IMR-32 cells	**Decreased** NO production<br>**Decreased** DLD-1 cell viability<br>**Decreased** IMR-1 cell viability	IC_{50}= 6.03µM<br>IC_{50}= 4.16µM<br>IC_{50}= 5.24µM	AC, CP	*Hypericum nokoense* Ohwi, AP, MeOH	**[168]**
**Xanthone**	Euxhanthone	NO production	*In vitro* RAW264.4 cells	**Decreased** NO production	IC_{50}= 7.67µM	CP	*Hypericum nokoense* Ohwi, AP, MeOH	**[168]**

Compound Class	Compound	Measurement	Method	Outcome		Therapeutic Application	Species, Plant Part and Extract	Ref #
**Xanthone derived compound**	3′-hydroxymethyl-2′-(4″-hydroxy-3″,5″-dimethoxyphenyl)-5′,6′:5,6-(6,8-dihydroxyxanthone)-1′,4′-dioxane	Cell viability	*In vitro* CCRF-CEM, CEM/ADR5000, U87.MG, U87.MGΔEGFR, HCT116($p53^{+/+}$), HCT116($p53^{-/-}$), MDA-MB-231-pcDNA3 and MDA-MB-231-BCRP cancer cell lines	**Decreased** cell viability (selective cytotoxicity)	IC_{50}= >91.74 - 12.72±0.75 µM vs IC_{50}=122.96±10.94 - 0.02±0.00 µM Doxorubicin control	AC	*Hypericum roeperianum* Schimp. ex A.Rich., B, MeOH	[212]
**Xanthone derived compound**	3′-hydroxymethyl-2′-(4″-hydroxy-3″,5″-dimethoxyphenyl)-5′,6′:5,6-(xanthone)-1′,4′-dioxane	Cell viability	*In vitro* CCRF-CEM, CEM/ADR5000, U87.MG, U87.MGΔEGFR, HCT116($p53^{+/+}$), HCT116($p53^{-/-}$), MDA-MB-231-pcDNA3 and MDA-MB-231-BCRP cancer cell lines	**Decreased** cell viability (selective cytotoxicity)	IC_{50}= >85.47 - 16.31±2.12 µM vs IC_{50}=122.96±10.94 - 0.02±0.00 µM Doxorubicin control	AC	*Hypericum roeperianum* Schimp. ex A.Rich., B, MeOH	[212]

Compound Class	Compound	Measurement	Method	Outcome		Therapeutic Application	Species, Plant Part and Extract	Ref #
**Xanthone derived compound**	1101	Stress-induced depressive behaviours	*In vivo* mouse model	**Increased** swimming period in mice (FST) **Decreased** immobility period in mice (FST) **Increased** activity time in mice (TST) **Decreased** immobility period in mice (TST)	No significant difference between tested compound and venlafaxine positive control	AD	*Hypericum* spp., -, -	[226]
**Xanthone derived compound**	1105	Stress-induced depressive behaviours	*In vivo* mouse model	**Increased** swimming period in mice (FST) **Decreased** immobility period in mice (FST) **Increased** activity time in mice (TST) **Decreased** immobility period in mice (TST)	No significant difference between tested compound and venlafaxine positive control	AD	*Hypericum* spp., -, -	[226]
**β-diketone**	2,6,9-trimethyl-8-decene-3,5-dione	Acetylcholinesterase activity	*In vitro*	**Decreased** AChE activity	IC_{50}= 1.51 µM vs IC_{50}= 0.13 µM Physostigmine control	AD	*Hypericum perforatum* L., AP, Diethyl ether	[227]

Table S1. Abbreviations: AP – aerial part; R – roots; S – stems; L – leaves; B – bark; Fl – flowers; Fr – fruits; Sb – stem bark; Tw – twigs; WP – whole plant; SH – skin healing; CP – cell protection; AC – anticancer; AP – antiparasitic; AD – antidepressant; AM – antimicrobial; AV – antiviral; ADb – antidiabetic; AI – anti-inflammatory; Ad.In – Adipogenesis inhibition; IM – immunomodulatory; Alz – Alzheimer; Mal – malaria; Cosm – cosmetic; AF – antifungal; AN – analgesia; Vd – vasodilation; AEp – anti epilepsy; PDT – photodynamic therapy; Int – interactions; Sp.At – spinocerebellar ataxia; EtOH – ethanol; MeOH – methanol; DCM – dichloromethane; AChE - acetylcholinesterase
